# Separation of low and high grade colon and rectum carcinoma by eukaryotic translation initiation factors 1, 5 and 6

**DOI:** 10.18632/oncotarget.20642

**Published:** 2017-09-05

**Authors:** Nicole Golob-Schwarzl, Caroline Schweiger, Carina Koller, Stefanie Krassnig, Margit Gogg-Kamerer, Nadine Gantenbein, Anna M. Toeglhofer, Christina Wodlej, Helmut Bergler, Brigitte Pertschy, Stefan Uranitsch, Magdalena Holter, Amin El-Heliebi, Julia Fuchs, Andreas Punschart, Philipp Stiegler, Marlen Keil, Jens Hoffmann, David Henderson, Hans Lehrach, Christoph Reinhard, Christian Regenbrecht, Rudolf Schicho, Peter Fickert, Sigurd Lax, Johannes Haybaeck

**Affiliations:** ^1^ Institute of Pathology, Medical University of Graz, Graz, Austria; ^2^ Center for Biomarker Research in Medicine, Graz, Austria; ^3^ Institute of Molecular Biosciences, Karl-Franzens-University of Graz, Graz, Austria; ^4^ Department of Surgery, Hospital Brothers of Charity Graz, Graz, Austria; ^5^ Institute of Medical Informatics, Statistics and Documentation, Medical University of Graz, Graz, Austria; ^6^ Institute of Cell Biology, Histology and Embryology, Medical University Graz, Graz, Austria; ^7^ Department of Surgery, Medical University of Graz, Graz, Austria; ^8^ Experimental Pharmacology & Oncology Berlin GmbH-Berlin-Buch, Berlin, Germany; ^9^ Bayer AG, Berlin, Germany; ^10^ Max Planck Institute for Molecular Genetics, Berlin, Germany; ^11^ Eli Lilly & Company, Indianapolis, USA; ^12^ Cpo – cellular phenomics & oncology Berlin-Buch GmbH, Berlin, Germany; ^13^ Institute of Experimental and Clinical Pharmacology, Medical University of Graz, Graz, Austria; ^14^ Division of Gastroenterology and Hepatology, Medical University of Graz, Graz, Austria; ^15^ Department of Pathology, Hospital Graz South-West, Austria; ^16^ Department of Pathology, Otto-von-Guericke-University Magdeburg, Magdeburg, Germany

**Keywords:** colorectal carcinoma, liver metastases, eukaryotic translation initiation factors, PI3K/AKT/mTOR pathway

## Abstract

Colorectal cancer (CRC) is the third most common cause of cancer related death worldwide. Furthermore, with more than 1.2 million cases registered per year, it constitutes the third most frequent diagnosed cancer entity worldwide. Deregulation of protein synthesis has received considerable attention as a major step in cancer development and progression. Eukaryotic translation initiation factors (eIFs) are involved in the regulation of protein synthesis and are functionally linked to the phosphatidylinositol-3-kinase (PI3K)/AKT/mammalian target of rapamycin (mTOR) signaling pathway.

The identification of factors accounting for colorectal carcinoma (CRC) development is a major gap in the field. Besides the importance of eIF3 subunits and the eIF4 complex, eIF1, eIF5 and eIF6 were found to be altered in primary and metastatic CRC. We observed significant difference in the expression profile between low and high grade CRC. eIF1, eIF5 and eIF6 are involved in translational control in CRC. Our findings also indicate a probable clinical impact when separating them into low and high grade colon and rectum carcinoma.

eIF and mTOR expression were analysed on protein and mRNA level in primary low and high grade colon carcinoma (CC) and rectum carcinoma (RC) samples in comparison to non-neoplastic tissue without any disease-related pathology. To assess the therapeutic potential of targeting eIF1, eIF5 and eIF6 siRNA knockdown in HCT116 and HT29 cells was performed. We evaluated the eIF knockdown efficacy on protein and mRNA level and investigated proliferation, apoptosis, invasion, as well as colony forming and polysome associated fractions.

These results indicate that eIFs, in particular eIF1, eIF5 and eIF6 play a major role in translational control in colon and rectum cancer.

## INTRODUCTION

Colorectal cancer (CRC) is the third most common cause of cancer related death and with more than one million cases annually the third most frequently diagnosed cancer entity worldwide [1–3]. Major risk factors include high fat intake, alcohol, red meat, obesity, smoking, age and physical inactivity [4, 5]. Approximately 20% of CRC patients have liver metastases at the time of diagnosis and 60% of patients develop liver metastases during the course of disease [6–8]. Current clinical management strategies include surgery, chemotherapy, radiation and palliative care, but they are not as effective as previously expected [4]. Various drugs have been reported to be effective against primary or metastatic CRC, but still the efficacy of current medications needs to be further improved. Although biologically similar, it needs to be taken into account, that carcinomas of the colon (CC) and rectum (RC) are treated differently with respect to surgery and radiotherapy [9].

**Figure 1:**
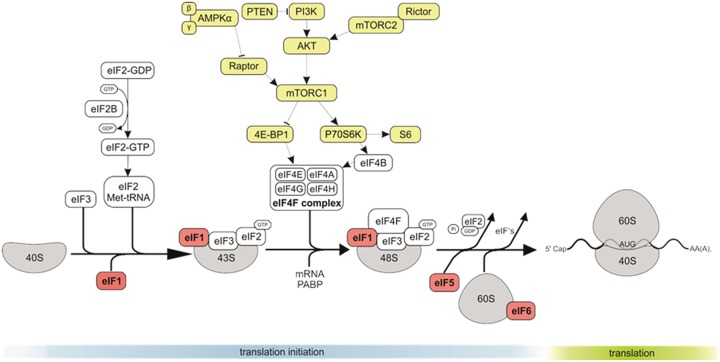
The role of eIF1, eIF5 and eIF6 in CRC The eukaryotic translation initiation starts with the separation of 40S and 60S ribosomal subunits, and formation of an 80S ribosomal initiation complex. Formation of a 43S preinitiation complex comprising a 40S subunit, eIF1, eIF1A, eIF3, eIF2–GTP–Met-tRNA^Met^_i_ and probably eIF5. The next step is mRNA activation, followed by attachment of the 43S complex to the activated mRNA region. Scanning of the 5′ UTR by 43S complex. Recognition of the initiation codon and 48S initiation complex formation. The next step is joining of 60S subunits to 48S complexes and concomitant displacement of eIF2–GDP and other factors (eIF1, eIF3, eIF4B, eIF4F and eIF5). GTP hydrolysis by eIF5B and release of eIF1A and GDP-bound eIF5B. Termination follows elongation and leads to recycling.

Deregulation of protein synthesis has received considerable attention as a major step in cancer development and progression [10]. Protein synthesis is regulated at multiple stages, including translation of mRNA into proteins (Figure 1). Studies suggest that ribosomal protein synthesis plays a direct role during tumor initiation. Translation can be divided into 4 stages, namely initiation, elongation, termination, and ribosomal recycling, of which initiation is the rate-limiting step of protein synthesis in eukaryotes. The process of translation initiation starts with the formation of a 43S pre-initiation complex composed of the 40S small ribosomal subunit, methionine tRNAi and a group of eukaryotic inititation factors (eIFs) (Figure 1). Subsequently, this 43S pre-initiation complex binds to the 5’ end of mRNA and then to eIF4F. Besides the eIF4F complex, other eIFs such as eIF1, eIF5, eIF6 and the large eIF3 complex comprising multiple subunits are involved in the initial translation regulation [10]. eIF1 is an essential mediator of start codon recognition and acts as negative regulator. eIF5 contains an unusual amino acid hypusine, which is important for eukaryotic cell proliferation. Two isoforms of eIF5 with high sequence homology undergo hypusination at the same specific lysine residue [11]. The expression of eIF5 has been shown to be upregulated in many cancer entities [12] and is thought to play a role in the regulation of cell proliferation and apoptosis [13]. Nevertheless, the exact mechanistic role of eIF5 in tumorigenesis is unknown.

The interaction of eIF1 and eIF3C has been shown *in vitro* and is essential for the recruitment of eIF1 to the 40S ribosomal subunit by eIF3 during initiation of protein translation [14].

eIF6 is mostly in the cytoplasm (although a minor pool is essential for nucleolar maturation of 60S subunits), and has anti-association property, by blocking premature 60S joining to 40S (Figure 1) [15–20]. eIF6 was found to be overexpressed in some cancer types, particularly in metastatic CRC [21].

We investigated the expression of members of the eIF family, focusing on eIF1, eIF5, and eIF6, together with components of the mammalian target of rapamycin (mTOR) signaling cascade. We analyzed the expression levels in primary low and high grade CC and RC as well as their liver metastases and corresponding non-neoplastic colorectal mucosa tissues (NNT). Finally, we assessed the therapeutic potential of targeting eIFs by performing siRNA knockdown experiments for eIF1, eIF5 and eIF6 in two CRC cell lines (HCT116, HT29).

## RESULTS

### High expression of eIF1, eIF5 and eIF6 predicts poor prognosis of human CRC

**Figure 2:**
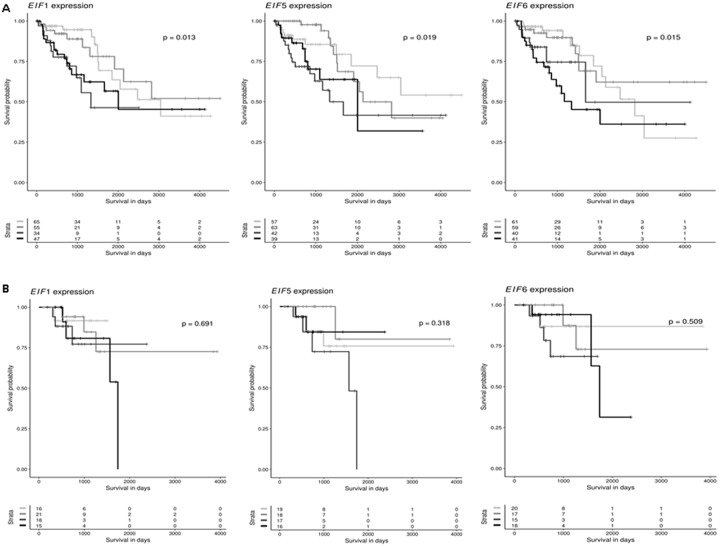
eIF1, eIF5 and eIF6 are clinically relevant candidates in CRC **(A)** Kaplan-Meier curves reflect the effect of eIF1, eIF5 and eIF6 expression on overall survival for CC. Cases are divided in eIF1, eIF5 and eIF6 low or high expressers according to whether expression is below or above median and survival is compared using the log-rank test. **(B)** Kaplan-Meier curves reflect the effect of eIF1, eIF5 and eIF6 expression on overall survival for RC. Cases are divided in eIF1, eIF5 and eIF6 low or high expressers according to whether expression is below or above median and survival is compared using the log-rank test.

The TCGA database was investigated to identify mTOR members and eIF genes that are significantly altered in CRC. Kaplan-Meier curves were drawn to assess a potential association of mTOR members and eIF expression and overall survival in CC and RC patients. The median mTOR and eIF mRNA expression in all CC and RC tissues was used as the cutoff point to divide all cases into low and high grade CC (n = 201) and RC (n = 70) groups. As shown in Figure 2A there was a significant difference in the survival between patients of low and high grade CC for eIF1 (p = 0.013), eIF5 (p = 0.019) and eIF6 (p = 0.015). However, gene expression of eIF1, eIF5 and eIF6 had no significant influence on overall survival between low and high grade RC patients (Figure 2B and 2C).

Additionally, to eIF1,5 and 6 also other eIF subunits were investigated regarding their influence on overall survival. As shown in Supplementary Figure 5A – 5F, there was a significant difference in the survival between patients of low and high grade CC for eIF2S1 (p = 0.024), eIF3A (p = 0.011), eIF3B (p = 0.013), eIF3C (p = 0.013), eIF3D (p = 0.022) and eIF3H (p = 0.024) group. There were no significant differences in overall survival between RC low and high grade groups (Supplementary Figure 6A - 6E) for eIF2S1, eIF3A, eIF3B, eIF3C, eIF3D and eIF3H. In Supplementary Figure 7A – 7F, there was a significant difference in the survival between patients of low and high grade CC for eIF3I (p = 0.008), eIF3J (p = 0.026), eIF3K (p = 0.006), eIF3M (p = 0.018), eIF4B (p = 0.004) and eIF4E (p = 0.003) group. There were no significant differences in overall survival between low and high grade RC groups (Supplementary Figure 8A - 8E) for those genes. Besides for eIF3M (p = 0.018). In Supplementary Figure 9A – 9C, there was a significant difference in the survival between patients of low grade and high grade CC for eIF4G1 (p = 0.005), eIF4G2 (p = 0.011), and eIF4G3 (p = 0.011) group. There were no significant differences for eIF4G1 and eIF4G3, for eIF4G2 (p = 0.011) there was a significant difference in the survival between low and high grade RC groups (Supplementary Figure 10).

To also investigate the influence of upstream signaling, also mTOR cascade members and their influence on overall survival of CRC patients. As shown in Supplementary Figure 1A – 1F, there was a significant difference in the survival between patients of low and high grade CC for mTOR (p = < 0.001), PTEN (p = 0.016), p70S6K (p = 0.016), AKT1 (p = 0.020), AKT2 (p = 0.024) and AKT3 (p = 0.021) group, but there were no significant differences in overall survival between low and high grade RC groups (Supplementary Figure 2A - 2F) for mTOR, PTEN, p70S6K, AKT1, AKT2 and AKT3 group. As shown in Supplementary Figure 3A – 3E, there was a significant difference in the survival between patients of low and high grade CC for AMPK1 (p = < 0.010), AMPK (p = 0.011), Rictor (p = 0.017), Raptor (p = 0.016) and RPS6 (p = 0.019) group. There were no significant differences in overall survival between RC low and high grade groups (Supplementary Figure 4A - 4E) for the mentioned genes. To summarize the results from the overall survival analyses, eIF1, eIF5, eIF6 as well as other eIF subunits and PI3K/AKT/mTOR pathway members had a significant influence on the overall survival of CC and RC patients. This strengthens our hypothesis, that low and high grade tumors should be separated due to their molecular differences in eIF and mTOR signaling pathways.

### eIF expression is a marker in low and high grade CC and RC

**Figure 3:**
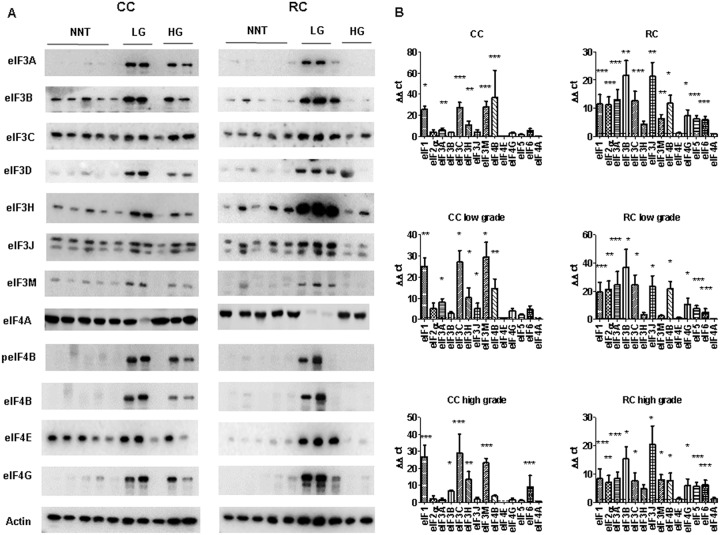
eIF expression levels in low and high grade CC and RC **(A)** Graph shows Western blot analysis of low grade (LG) and high grade (HG) CC and RC compared to non- NNT. Equal amounts of protein have been resolved on SDS PAGE and immunoblotted with various eIF subunits and β-actin (loading control) antibodies. **(B)** qRT-PCR of various eIF subunits from LG and HG CC and RC compared NNT. Three independent experiments were carried out. Bars represent mean ± SEM. ^*^p < 0.05, ^**^p < 0.01, ^***^p < 0.001. Statistical analysis: 2-way ANOVA with Bonferroni posttest.

**Figure 4:**
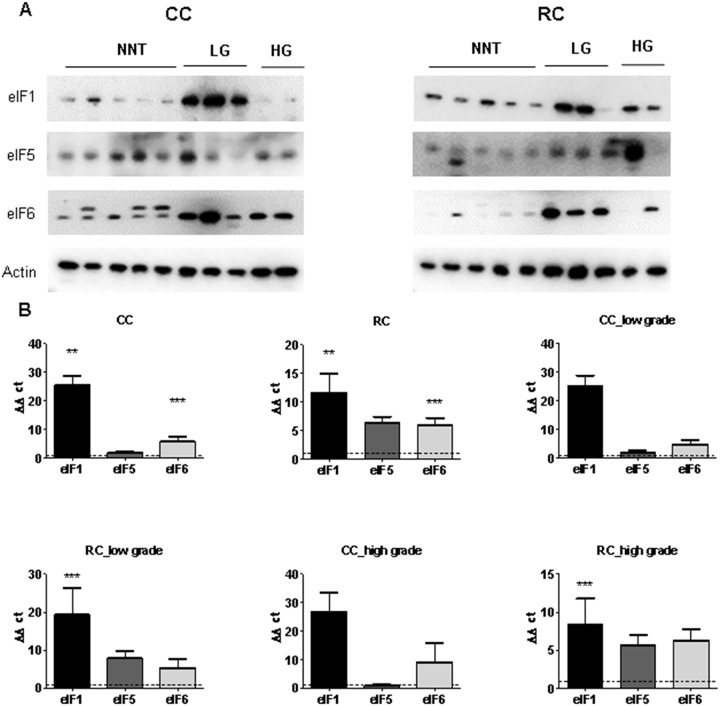
eIF1, eIF5 and eIF6 expression levels in low and high grade CC and RC **(A)** Western blot of eIF1, eIF5 and eIF6 from LG and HG CC and RC NNT. Equal amounts of protein from each pair were resolved on SDS PAGE and immunoblotted with antibodies directed against eIF1, eIF5, eIF6 and β-actin (loading control). **(B)** qRT-PCR of eIF1, eIF5 and eIF6 from LG and HG CC and RC compared to NNT. Three independent experiments were carried out. Bars represent mean ± SEM. ^***^p < 0.01, ^***^p < 0.001. Statistical analysis: 2-way ANOVA with Bonferroni posttest.

We first performed a basic characterization of eIFs on protein and mRNA level in CRC samples compared to NNT (Figure 3 and 4). For this purpose we separated CC and RC into low and high grade tumors and performed immunoblots and qRT-PCR.

peIF2α, eIF2α, eIF3A, eIF3B, eIF3C, eIF3H, eIF3J, eIF3M, eIF4A, peIF4B, eIF4B, eIF4E and eIF4G showed higher protein expression in CRC tumors in comparison to NNT (Figure 3A and Supplementary Figure 11).

To evaluate the gene expression at the mRNA level, we performed qRT–PCR and measured the transcripts of 13 eIFs by relative quantification normalized to β-actin. Our results show that transcripts for the eIFs *1, 2α, 3A, 3B, 3C, 3H, 3J, 3M, 4B, 4G, 5* and *6* showed a significant overexpression in CRC compared to NNT (Figure 3B, Supplementary Tables 1, 2 and 3). The mRNA expression of several eIF subunits differed between CC and RC. Interestingly, peIF2α, eIF2α and the eIF3 initiation factors A, B, C, H and I as well as peIF4B, eIF4G, and eIF6 displayed a higher protein expression relative to NNT and RC compared to CC (Figure 3A, Supplementary Figure 11 and Supplementary Figure 12). eIF1, eIF4B and eIF5 showed increased protein levels in CC and RC compared to NNT (Figure 3A, Figure 4A, Supplementary Figure 11 and Supplementary Figure 12).

**Table 1:** Clinical and pathological characteristics of 40 patients with colorectal carcinoma. For each biochemically assessed CC and RC patient, numbered from 1 to 40, the clinical and pathological characteristics, including sex, age, TNM, stage, histological type and the presence of positive lymph nodes are listed

Patient	Gender	Age	Localization	TNM	Stage	Histological type
1	M	66	Colon	pT1 N0 M0	I	well differentiated adenocarcinoma
2	W	62	Transverse Colon	pT2 pN0	I	well differentiated mucinous adenocarcinoma
3	W	65	Colon	pT3b pN0	IIA	moderatly differentiated mucinous adenocarcinoma
4	W	77	Ascending Colon	pT3b pN0	IIA	moderatly differentiated mucinous adenocarcinoma
5	W	89	Sigmoid Colon	pT3b pN0	IIA	moderatly differentiated adenocarcinoma
6	M	58	Sigmoid Colon	pT3a pN0	IIA	moderatly differentiated invasive adenocarcinoma
7	M	74	Ascending Colon	pT3 pN0	IIA	moderatly differentiated adenocarcinoma
8	M	76	Sigmoid Colon	pT3b pN0	IIA	moderatly differentiated adenocarcinoma
9	M	65	Transverse Colon	pT3a pN0	IIA	moderatly differentiated adenocarcinoma
10	M	79	Sigmoid Colon	pT3a pN0	IIA	moderatly differentiated adenocarcinoma
11	M	48	Sigmoid Colon	pT4a N0	IIB	well differentiated adenocarcinoma
12	W	75	Cecum	pT2 pN1a	IIIA	moderatly differentiated adenocarcinoma
13	M	71	Sigmoid Colon	pT4a N2a M0	IIIC	moderatly differentiated adenocarcinoma
14	M	82	Sigmoid Colon	pT4a L1 V1 N2b	IV	low differentiated adenocarcinoma
15	M	78	Sigmoid Colon	pT3 N2a M1a	IVA	moderatly differentiated adenocarcinoma
16	W	52	Sigmoid Colon	pT4a pN2a V1	IVA	moderatly differentiated adenocarcinoma
17	M	74	Sigmoid Colon	pT3 N1a M1a	IVA	moderatly differentiated adenocarcinoma
18	M	71	Rectum	pT2 N0 M0	I	moderatly differentiated adenocarcinoma
19	W	58	Rectum	pT2 N0 M0	I	moderatly differentiated adenocarcinoma
20	W	83	Rectum	pT2 pN0	I	moderatly differentiated adenocarcinoma
21	M	61	Rectum	pT2 N0 M0	I	moderatly differentiated adenocarcinoma
22	W	64	Rectum	pT3a pN0	IIA	moderatly differentiated adenocarcinoma
23	M	77	Rectum	pT3b pN0	IIA	moderatly differentiated adenocarcinoma
24	M	56	Rectum	pT3 N0 M0	IIA	moderatly differentiated adenocarcinoma
25	W	76	Rectum	pT3 N0 M0	IIA	moderatly differentiated adenocarcinoma
26	M	78	Rectum	pT3 N0 M0	IIA	moderatly differentiated adenocarcinoma
27	W	51	Rectum	pT3 N0 M0	IIA	moderatly differentiated adenocarcinoma
28	M	67	Rectum	pT3 N0 M0	IIA	moderatly differentiated adenocarcinoma
29	M	77	Rectum	pT3 N0 M0	IIA	moderatly differentiated adenocarcinoma
30	W	84	Rectum	pT4a NB1b M0	IIIA	moderatly differentiated adenocarcinoma
31	M	55	Rectum	pT3 N2b	IIIB	highly differentiated adenocarcinoma
32	M	60	Rectum	pT3 NB1b	IIIB	highly differentiated adenocarcinoma
33	W	62	Rectum	pT3 N2a M0	IIIB	highly differentiated adenocarcinoma
34	W	76	Rectum	pT3 N1a M0	IIIB	highly differentiated adenocarcinoma
35	W	47	Rectum	pT4a NB1b Mx	IIIC	highly differentiated adenocarcinoma
36	M	55	Rectum	pT3 N2b	IIIC	highly differentiated adenocarcinoma
37	M	73	Rectum	pT4b N1c M1a	IV	highly differentiated adenocarcinoma
38	W	58	Rectum	pT3 N2a M1b	IVB	highly differentiated adenocarcinoma
39	W	58	Rectum	pT3 N2a Mx	IVB	highly differentiated adenocarcinoma
40	M	62	Rectum	pT3 N1a M0	IIIB	highly differentiated adenocarcinoma

Furthermore, *eIF1, eIF2α, eIF3A, eIF3B, eIF3C, eIF3H, eIF3J, eIF3M, eIF4B, eIF4G* and *eIF6* showed a significantly higher mRNA expression level in CC compared to NNT (Figure 3B). For RC patients, we observed mRNA overexpression for the eIFs *1, 2α, 3A, 3B, 3C, 3H, 3J, 3M, 4B, 4G, 5* and *6* (Figure 3B and Figure 4B) compared to NNT.

As we noticed major differences in the protein expression between CC and RC patients, we decided to separate the results into low and high grade tumors. In low grade CC, we observed an overexpression of eIF1 and eIF4B on protein level and in high grade CC only an overexpression of eIF1 (Figure 3A, Figure 4A, Supplementary Figure 11 and Supplementary Figure 12). The eIFs *1, 2α, 3A, 3C, 3H, 3J, 3M, 4B, 4G* and *6* (Figure 3B and Figure 4B) showed a significant overexpression on mRNA level in low grade CC tumors. In contrast, the eIFs *1, 3B, 3C, 3H, 3M, 4B* and *6* (Figure 3B and Figure 4B) revealed a significantly higher expression in high grade CC tumors.

However, in low grade RC the protein expression levels of the eIFs 1, p2α, 2α, 3A, 3C, p4B, 4G, 5 and 6 were significantly increased relative to NNT compared to low grade CC (Figure 3A, Figure 4A, Supplementary Figure 11 and Supplementary Figure 12). Increased mRNA expression levels of *eIFs 1, 2α, 3A, 3B, 3C, 3H, 3J, 4B, 4G, 5* and *6* (Figure 3B, and Figure 4B) were observed in low grade RC. Increased protein expression levels of eIF1, eIF2α, eIF3A, eIF3B, eIF3C, eIF3I, eIF3H, peIF4B, eIF4B, eIF4E, eIF5 and eIF6 (Figure 3A, Figure 4A, Supplementary Figure 11 and Supplementary Figure 12) were observed in high grade RC. In high grade RC the eIFs *1, 2α, 3A, 3B, 3C, 3H, 3J, 3M, 4B, 4G, 5* and *6* (Figure 3B and Figure 4B) showed a significantly higher mRNA expression relative to NNT compared to high grade CC (Supplementary Tables 4 and 5).

### Silencing of eIF1, eIF5 and eIF6 in HCT116 cells and HT29 cells

Based on the results of the eIF basic characterization in CRC patients (Figure 3, Figure 4, Supplementary Figure 11 and Supplementary Figure 12), eIF1, eIF5 and eIF6 were identified as novel factors, which are significantly activated in CRC (Supplementary Tables 1 and 2) and might therefore represent potential targets for future therapeutic intervention.

**Figure 5:**
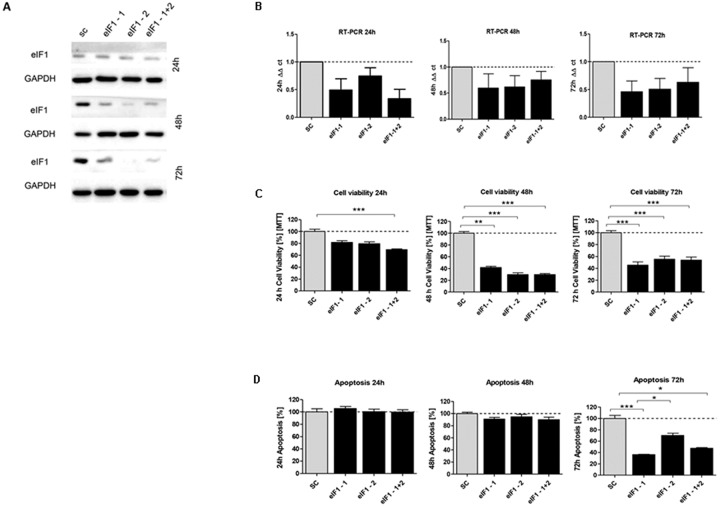
*In vitro* characterization of eIF1 knockdown effect in HCT116 cells **(A)** Protein expression of eIF1-siRNA knockdown after 24h, 48h and 72h compared to SC control **(B)** mRNA expression level of *eIF1* in HCT116 cells compared to the control group. **(C)** Cell viability in HCT116 cells transfected with eIF5 siRNA after 24h, 48h and 72h **(D)** Graphs show apoptosis rate after eIF1 knockdown compared to the SC after 24h, 48h and 72h. Three independent experiments were carried out. Bars represent mean ± SEM. ^*^p < 0.05, ^**^p < 0.01, ^***^p < 0.001. Statistical analysis: 2-way ANOVA with Bonferroni posttest.

**Figure 6:**
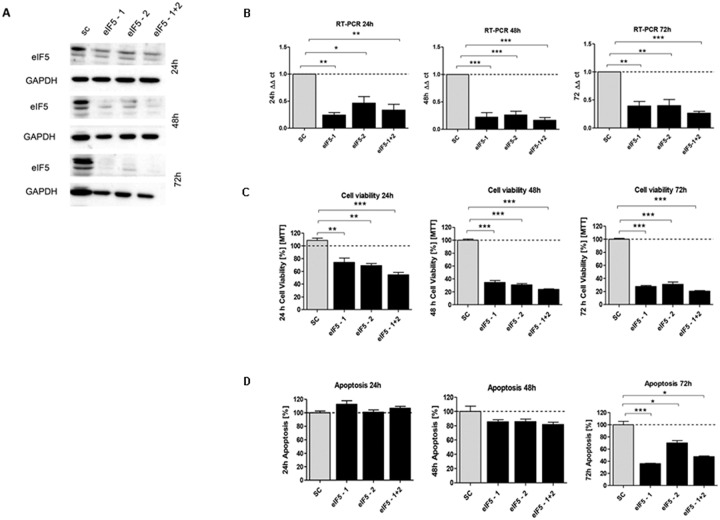
*In vitro* characterization of eIF5 knockdown effect in HCT116 cells **(A)** Protein expression of eIF5-siRNA knockdown after 24h, 48h and 72h compared to SC. **(B)** mRNA expression level of *eIF5* in HCT116 cells compared to SC. **(C)** Cell viability in HCT116 cells transfected with eIF5 siRNA after 24h, 48h and 72h. **(D)** Graphs show apoptosis rate after eIF5-siRNA knockdown compared to the SC after 24h, 48h and 72h. Three independent experiments were carried out. Bars represent mean ± SEM. ^*^p < 0.05, ^**^p < 0.01, ^***^p < 0.001. Statistical analysis: 2-way ANOVA with Bonferroni posttest.

**Figure 7:**
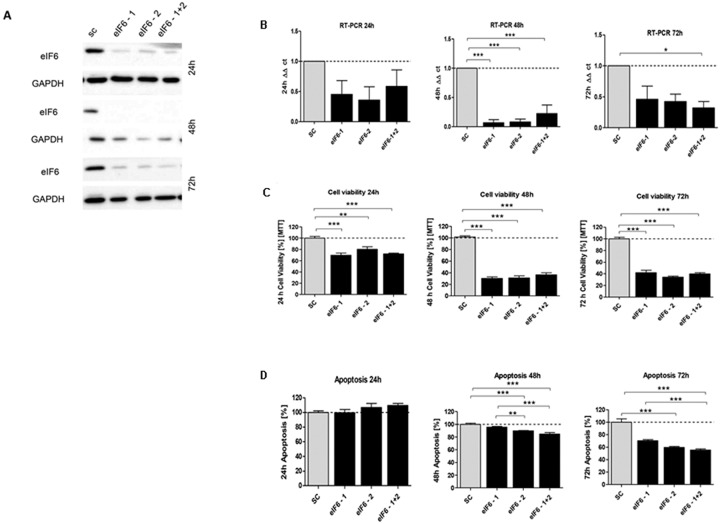
*In vitro* characterization of eIF6 knockdown effect in HCT116 cells **(A)** Protein expression of eIF6-siRNA knockdown after 24h, 48h and 72h compared to SC control. **(B)** mRNA expression level of *eIF6* in HCT116 cells compared to SC. **(C)** Cell viability in HCT116 cells transfected with eIF5 siRNA after 24h, 48h and 72h. **(D)** Graphs show apoptosis rate after eIF6 knockdown compared to the SC after 24h, 48h and 72h. Three independent experiments were carried out. Bars represent mean ± SEM. ^*^p < 0.05, ^**^p < 0.01, ^***^p < 0.001. Statistical analysis: 2-way ANOVA with Bonferroni posttest.

In order to investigate the effect of silencing of *eIF1*, *eIF5* and *eIF6*, HCT116 cells were transfected with corresponding siRNA constructs and the knockdown effect was assessed for three time points. A knockdown effect at protein level close to 90% was achieved for eIF1 (Figure 5A), eIF5 (Figure 6A) and eIF6 (Figure 7A) at all three time points. The transfection strongly reduced the proliferation of HCT116 cells which expressed eIF1, eIF5 and eIF6 specific siRNAs, but had no effect on MOCK control.

Upon transfecting HCT116 cells for 24h, 48h and 72h with the respective siRNAs, mRNA expression of *eIF1* (Figure 5B), *eIF5* (Figure 6B) and *eIF6* (Figure 7B) was reduced for all three initiation factors compared to cells transfected with scrambled RNA. The effect of *eIF1, eIF5* and *eIF6* gene knockdown on apoptosis was analyzed by YO-PRO®-1 staining. The apoptosis rate of cells transfected with *eIF1* (Figure 5D), *eIF5* (Figure 6D) and *eIF6* siRNAknockdown constructs (Figure 7D) was significantly decreased compared to negative control cells 72h after transfection.

Silencing of *eIF1* (Figure 5C), *eIF5* (Figure 6C) and *eIF6* (Figure 7C) led to a significant reduction of cell viability at all 3 time points (24h, 48h, 72h).

In addition, we evaluated the consequences of siRNA-mediated *eIF1*, *eIF5* and *eIF6* depletion in HT29 cells. The knockdown effect was assessed at three time points (24h, 48h and 72h). A knockdown effect at protein level close to 60% was achieved for eIF1 (Supplementary Figure 13A), eIF5 (Supplementary Figure 14A) and eIF6 (Supplementary Figure 15A) at all three time points.

The mRNA expression of *eIF1* (Supplementary Figure 13B), *eIF5* (Supplementary Figure 14B) and *eIF6* (Supplementary Figure 15B) was reduced for all three subunits compared to cells transfected with scrambled RNA. Silencing of *eIF1* (Supplementary Figure 13C), *eIF5* (Supplementary Figure 14C) and *eIF6* (Supplementary Figure 15C) led to a lower reduction of cell viability at all 3 time points (24h, 48h, 72h) in HT29 cells in comparison to HCT116 knockdown. The apoptosis rate of cells transfected with *eIF1* (Supplementary Figure 13D), *eIF5* (Supplementary Figure 14D) and *eIF6* siRNAknockdown constructs (Supplementary Figure 15D) displayed lower decrease only at the 72h time point.

Clonogenicity was evaluated by Giemsa staining. Colony formation was reduced 14 days after seeding in all transfected cells (Supplementary Figure 16A - 16C). The effect of *eIF1*, *eIF5A* and *eIF6* knockdown on CRC cell motility was investigated by assessing the transmigration competence of cells through filters coated with an extracellular matrix. The cells exhibited a reduced capability to transmigrate upon *eIF1*, *eIF5* and *eIF6* (Supplementary Figure 18D - 18F) knockdown compared to control cells.

### Knockdown of eIF1, eIF5 and eIF6 leads to reduced translation

The effects of *eIF1*, *eIF5* and *eIF6* knockdown on translation initiation were investigated by sucrose density gradient profiling. After sucrose density gradient centrifugation of cell lysates, polysomes, 80S ribosomes and free 40S and 60S subunits were detected by monitoring their A_254nm_ as described in the methods section.

Non-transfected HCT116 cells showed some free 40S and 60S subunits, a large 80S peak and low numbers of polysomes. After *eIF1* knockdown, increased levels of free 60S subunits and a marked decrease of the 80S peak were observed suggesting a defect in translation initiation. Furthermore, fewer polysomes were recorded in the *eIF1* knockdown profile, indicating reduced translation rates (Supplementary Figure 17A). *eIF5* knockdown also led to decreased levels of polysomes. In addition, the levels of free 40S and 60S ribosomal subunits relative to 80S ribosomes were increased, suggesting less efficient translation initiation (Supplementary Figure 17B). Similarly, *eIF6* knockdown resulted in a decrease in polysomes and an increase of the levels of free ribosomal subunits relative to 80S ribosomes (Supplementary Figure 17C). Additionally, we evaluated the sedimentation of the 40S subunit protein RPS6 by Western Blotting. Compared to the MOCK profile, RPS6 levels were reduced in the polysome fractions of eIF1- eIF5- and eIF6 silenced cells. This is in line with the reduced polysome levels observed in the recorded profiles and further confirms that knockdown of all three initiation factors resulted in a reduction of polysomes consistent with reduced initiation of translation.

In conclusion, knockdown of all three initiation factors resulted in a reduction of polysomes consistent with reduced initiation of translation.

### Effect of eIF1, eIF5 and eIF6 knockdown on apoptosis and proliferation

We evaluated the consequences of siRNA-mediated eIF1, eIF5 and eIF6 knockdown in HCT116 cells and HT29 cells, by measuring apoptosis and cell proliferation. We tested an alternative apoptotic response pathway by using western blotting and qRT-PCR to examine poly (ADP-ribose) polymerase (PARP) cleavage and cleaved caspase 3. The control cells showed a decrease at the time point 24h for all three siRNAs. Compared to control cells, eIF1, eIF5 and eIF6 knockdown resulted in increased PARP in HCT116 cells after 24h (Supplementary Figure 18A - 18C). The time points 48h and 72h displayed a decreased PARP in HCT116 cells for eIF1, eIF5 and eIF6 knockdown compared to the control cells (Supplementary Figure 18A - 18C). We next investigated the effect of eIF1, eIF5 and eIF6 depletion in HT29 cells. Compared to control cells, eIF1, eIF5 and eIF6 knockdown resulted in a less decreased PARP expression in HT29 cells for all three time points (Supplementary Figure 18D - 18F). We evaluated the consequences of siRNA-mediated eIF1, eIF5 and eIF6 knockdown in HCT116 cells on mRNA level (Supplementary Figure 19A – 19C). These results were also evaluated on mRNA level for the HT29 cells (Supplementary Figure 20A – 20C).

Compared to control cells after 24h, eIF1, eIF5 and eIF6 knockdown resulted in increased cleaved caspase 3 activity in HCT116 cells (Supplementary Figure 18A – 18C). The time points 48h and 72h displayed a decreased cleaved caspase 3 in HCT116 cells for eIF1, eIF5 and eIF6 knockdown compared to the control cells (Supplementary Figure 18A - 18C). These results were also evaluated on mRNA level (Supplementary Figure 21A – 21C).

The control cells and 24h eIF1, eIF5 and eIF6 knockdown constructs resulted in similar expression for cleaved caspase 3 in HT29 cells (Supplementary Figure 18D – 18F). After 48h eIF1, eIF5 and eIF6 knockdown resulted in decreased Cleaved Caspase 3 in HT29 cells compared to control cells (Supplementary Figure 18D – 18F). After 72h, eIF1 knockdown resulted in similar expression for Cleaved Caspase 3 in HT29 cells compared to control cells (Supplementary Figure 18D). The eIF5 knockdown resulted in decreased cleaved caspase 3 in HT29 cells compared to control cells after 72h (Supplementary Figure 18E). For eIF6, just eIF6-1+2 resulted in decreased cleaved caspase 3 in HT29 cells compared to control cells after 72h (Supplementary Figure 18F). These results were also evaluated on mRNA level (Supplementary Figure 22A – 22C).

We next investigated the effect of eIF1, eIF5 and eIF6 knockdown on cell proliferation by using western blotting and qRT-PCR to examine Ki67 as proliferation marker. Compared to control cells after 24h eIF1, eIF5 and eIF6 knockdown resulted in increased Ki67 in HCT116 cells. For the time points 48h and 72h we observed an increase in control cells compared to cells upon eIF1, eIF5 and eIF6 knockdown (Supplementary Figure 23A – 23C). Compared to control cells after 24h eIF1, eIF5 and eIF6 knockdown resulted in decreased Ki67 in HT29 cells (Supplementary Figure 23D – 23F). After 48h and 72h, eIF1 knockdown resulted in increased Ki67 in HT 29 cells compared to control cells (Supplementary Figure 23D). eIF5 and eIF6 knockdown did not result in changes compared to control cells for Ki67 in HT29 cells (Supplementary Figure 23E and Supplementary Figure 23F). These results were also evaluated on mRNA level for HCT116 cells (Supplementary Figure 24A – 24C) and HT29 cells (Supplementary Figure 25A – 25C).

### In situ detection of different eIFs by padlock probe approach

In order to localize and analyze the distribution of *eIF1*, *eIF5* and *eIF6* transcripts in CC and RC vs. NNT, we performed an mRNA-based *in situ* detection approach (Supplementary Figure 26 and Supplementary Figure 27). *In situ* detection allows for visualization of single mRNA transcripts (Supplementary Figure 26 and Supplementary Figure 27). *In situ* detection confirmed an overexpression at the mRNA level of *eIF1* and *eIF5* in colon carcinoma vs. NNT (p<0.001 and p<0.05) (Supplementary Figure 27A). In RC tissue, eIF5 was overexpressed compared to NNT (p<0.05) (Supplementary Figure 27B).

### PI3K/AKT/mTOR pathway member expression in CRC

We investigated the expression patterns of mTORC1 and mTORC2 members in CRC patient samples, because this pathway is preceded by eIFs.

**Figure 8:**
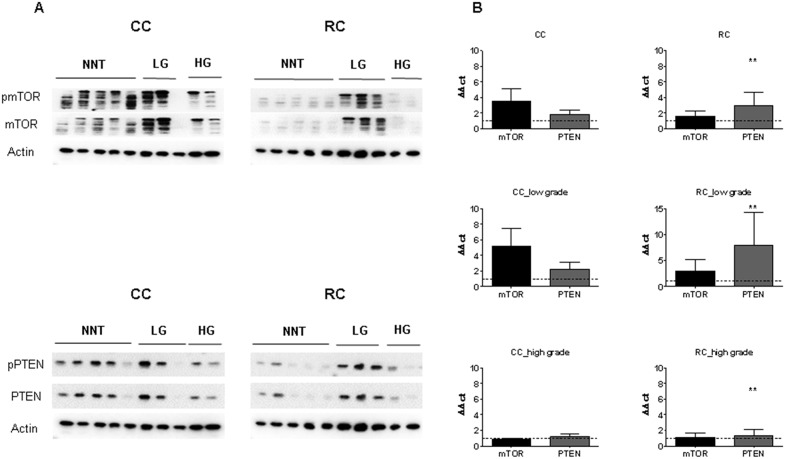
mTOR and PTEN expression in low and high grade CC and RC **(A)** Graph shows Western blot analysis of LG and HG CC and RC compared to NNT. Equal amounts of protein were resolved on SDS PAGE and immunoblotted with antibodies directed against pmTOR, mTOR, pPTEN, PTEN and β-actin (loading control) antibodies. **(B)** qRT-PCR analyses of mTOR and PTEN LG and HG CC and RC compared to NNT. Three independent experiments were carried out. Bars represent mean ± SEM. ^*^p < 0.05, ^**^p < 0.01, ^***^p < 0.001. Statistical analysis: 2-way ANOVA with Bonferroni posttest.

Immunoblot analyses of pRictor, Rictor, pAMPK, AMPK, pRaptor, Raptor, pmTOR, mTOR, pAKT, AKT, pPTEN, PTEN, pp70S6K, p70S6K, pRPS6, RPS6 and p4E-BP1, 4E-BP1 revealed a significantly higher expression of these proteins in CRC compared to the corresponding NNT (Figure 8A and Supplementary Figure 28). Increased mRNA expression levels of *mTOR* and *PTEN* were observed in CRC (Figure 8B). When we separated the samples into groups of CC and RC, however we observed that the overexpression of pmTOR, mTOR, pAKT, AKT, pp70S6K, p70S6K, p4E-BP1 and 4E-BP1 is restricted to the RC patients. The CC patient showed no significant change in the mTOR pathway expression at the protein level (Figure 8A and Supplementary Figure 28). Despite the differences in protein expression, increased mRNA expression levels of *mTOR* and *PTEN* (Figure 8B) were observed in both, CC and RC.

Finally, we investigated the influence of tumor grade and compared the protein expression levels after separation into low and high grade CC and RC. Protein expression levels of mTOR, pAKT, AKT, pPTEN, PTEN, pp70S6K, p70S6K, p4E-BP1 and 4E-BP1 were significantly increased in low grade RC compared to low and high grade CC (Figure 8A and Supplementary Figure 28). In comparison, pmTOR, mTOR, pAKT, AKT, pPTEN, PTEN, pp70S6K, p70S6K, p4E-BP1 and 4E-BP1 were increased in high grade RC at protein level (Figure 8A and Supplementary Figure 28). Increased mRNA expression levels of *mTOR* and *PTEN* (Figure 8B) were observed in low grade CC and RC, while there was no difference in the mRNA levels of *mTOR* and *PTEN* in high grade CC and RC compared to NNCRM (Figure 8B, Supplementary Table 4 and Supplementary Table 5).

The targets, mTORC1 and mTORC2, pRictor, Rictor, pAMPK, AMPK, pRaptor, Raptor, pRPS6 and RPS6 showed a decreased activation in low grade CC and RC and an increase in protein activation in high grade CC and RC (Supplementary Figure 28). We also analyzed the phosphorylation levels of pRictor, Rictor, pAMPK, AMPK, pRaptor, Raptor, pmTOR, mTOR, pAKT, AKT, pp70S6K, p70S6K, pRPS6, RPS6, p4E-BP1 and 4E-BP1 to assess the activity of mTORC1/C2 (Supplementary Figure 29A and 29B). The phosphorylation levels of pRictor/Rictor and pAKT/AKT resulted in an increased activity in high grade CC and RC. For low grade RC, the phosphorylation levels of pAMPK/AMPK, pRaptor/Raptor, pmTOR/mTOR, p4E-BP1/4E-BP1, pp70S6K/p70S6K and pRPS6/RPS6 resulted in increased activity in low grade tumors (Supplementary Figure 29B).

### PI3K/AKT/mTOR members and eIF expression in liver metastasis of primary colon and rectum carcinoma

As CRC tumors predominantly metastasize in the liver, expression profiles of mTOR members and eIFs in CC and RC metastases were analyzed by IHC, immunoblot and qRT-PCR compared to matched non-neoplastic liver tissue (NNLT).

**Figure 9:**
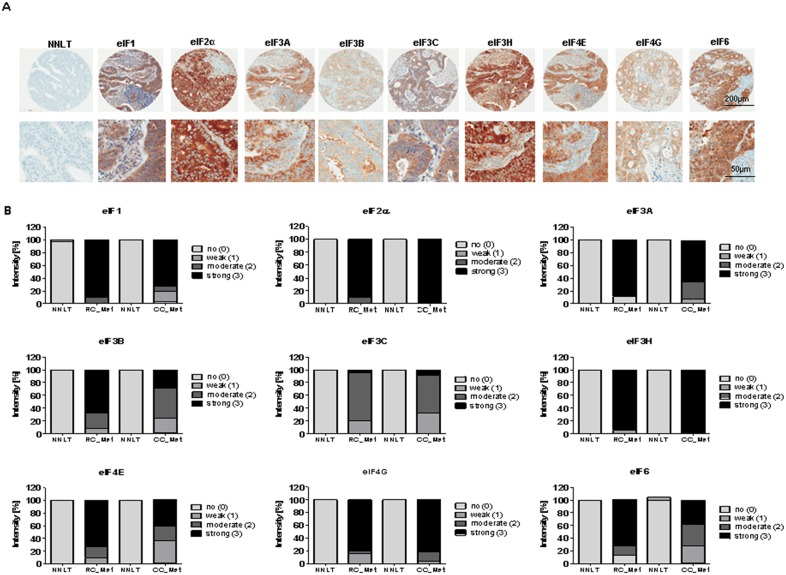
eIF expression in liver metastases of CC and RC compared to NNLT **(A)** Representative pictures of immunohistochemical stainings from liver metasteses of primary CRC. Scale bars: 200 μm and 50μm. **(B)** Densitometric analyses of immunohistochemical stainings from various eIF subunits in liver metasteses of primary CC (CC- Met) and RC (RC-Met) compared to non-neoplastic liver tissues (NNLT).

The IHC staining pattern of eIF subunits was found to be mainly cytoplasmic (Figure 9A). The subunits eIF1, 2α, 3H and 4G exhibited a stronger staining in the metastatic tissues compared to NNLT (Figure 9B). The staining intensities for eIF3A, eIF3B, eIF4E and eIF6 were increased compared to NNLT, but not as much as for the eIF subunits 1, 2α, 3H, 4G (Figure 9B). eIF3C staining was only weak to moderate in the metastases (Figure 9B). IHC staining intensity of the subunits eIF1, 2α, 3A, 3B, 3H, 4E, 4G and 6 was stronger in the RC metastases (RC-Met) and no staining was found in the NNLT (Figure 9B). Only for eIF3C was weak to moderate staining found in RC-Met (Figure 9B). For eIF5, we observed no changes in the staining intensity in metastasis samples compared to the NNLT. The IHC data displayed a stronger eIF expression in RC Met samples compared to CC-Met.

Regarding the PI3K/AKT/mTOR pathway in the metastases, we found no changes in the expression of pmTOR, mTOR, pAKT, AKT, pPTEN, PTEN, pp70S6K and 4E-BP1 at the protein level (Supplementary Figure 30A and 30B) in CC-Mets and RC-Mets. On mRNA level, *mTOR* and *PTEN* were significantly upregulated in CC- and RC-Met compared to NNLT (Supplementary Figure 30C).

Immunoblot analyses revealed peIF2α, eIF3B and eIF4E protein expression to be higher in metastatic CC and RC compared to NNLT (Supplementary Figure 30B). For eIF3K we only observed higher expression in RC-Met but no changes in CC-Met (Supplementary Figure 30B). To evaluate the gene expression at mRNA level, we performed qRT–PCR and measured the transcripts of 13 eIF subunits by relative quantification normalized to ß-actin. At the mRNA level, the eIF subunits *1, 2α, 3A, 3B, 3C, 3H, 3M, 4B, 4E, 4G, 5* and *6* showed a significant overexpression in CC- and RC-Met compared to NNLT (Supplementary Figure 30D).

We next investigated the effect on protein expression of primary CC and RC tumors compared to the respective liver metastasis for mTOR pathway members and eIF subunits. From the mTOR members only pmTOR resulted in increased protein level compared to low and high grade primary CC and RC (Supplementary Figure 31A and Supplementary Figure 31B). Next, we investigated the effect of eIFs on protein level in primary CC and RC compared to the respective liver metastasis. peIF2α and eIF3B displayed an increased protein level in liver metastases compared to low and high grade primary CC and RC (Supplementary Figure 32). We then evaluated the effect on protein expression in primary low and high grade RC compared to liver metastases, eIF3B and eIF3K resulted in an increase (Supplementary Figure 33).

## DISCUSSION

Recent studies on mRNA transcription and protein synthesis in cancer have demonstrated a key role for translational control in tumorigenesis [10]. Activation of mTOR signaling has been shown to be a hallmark of cancer and has been linked to cell growth and cell cycle progression [22–24].

In our study, we investigated the expression patterns of mTOR pathway components in CRC patients. We also separated the CRC patients into CC and RC and noticed remarkable differences in the expression patterns between these two groups. The final step was to sub-classify CC and RC patient samples into low and high grade tumors. mTOR and its upstream target AKT as well as the downstream targets p70S6K and 4E-BP1 displayed an overexpression only in RC, but not in CC. The tendency was the same in low and high grade RC tumors. Upstream and downstream targets of mTOR seem to be involved in cancer progression in low and high grade RC while in low grade and high grade CC the mTOR pathway members plays a less important role.

Previously, other groups found a significant increase of eIF3 subunits in CRC [25]. We also found that eIF3 subunits were differentially expressed at the protein and mRNA level in CC and RC. It is known from the literature, that eIF3A, eIF3B and eIF3M overexpression has been detected in the CC cell lines SW1116 [25–27]. eIF3C was found to be an oncogene and was shown to be increased in cancer cells [28], which is confirmed by our findings in CRC. eIF3H has been associated with CRC risk and was therefore suggested to act as CRC susceptibility gene [29]. We confirmed the CRC data from the literature and report differences in the expression pattern of eIF3 subunits in low and high grade CC and RC.

Previous studies have shown an overexpression of eIF4G and eIF4E in different cell lines, including CRC cell lines, and they have also been linked to carcinogenesis [30, 31]. This supports the results presented here showing that eIF4B displayed an overexpression in CRC and eIF4G is involved in RC formation. eIF1, eIF2, eIF3 and eIF5 have been reported as essential for translation initiation [32]. Previously, transient eIF2α expression was described as increased in normal cells, whereas constitutive overexpression supported tumor initiation and progression [1]. Knockdown of eIF3D in HCT116 cells attenuated proliferation and increased stress-driven apoptosis [4].

eIF4E is one of the most thoroughly investigated translation factors involved in cancer biology, especially in CRC. Together with eIF4A and eIF4G it forms the trimetric eIF4F complex [33]. eIF4E plays a major role in the regulation of tumor growth, invasion and metastasis [31, 34]. Compared to previous studies, we also showed that eIF2α, eIF4B, eIF4E and eIF4G were significantly overexpressed at protein and mRNA level in CRC. This implies the influence of the eIF4F complex in protein translation in CRC.

eIF6 expression limits cell growth and transformation [35]. It is known that eIF6 is part of a multi-protein complex connected with the RNA-induced silencing complex (RISC), which is the major complex regulating miRNA activity. Previous studies have reported eIF6 overexpression in ovarian serous carcinoma, leukemia, head and neck carcinoma, as well as CRC [18, 35–37]. We also saw a significant increase of eIF6 but only in low and high grade RC at protein and mRNA level. This finding suggests that eIF6 may play a central role in the translation initiation in RC.

eIF5 overexpression has been reported in different cancer types and is considered to be a predictive tumor marker [38]. eIF1 has been demonstrated to bind eIF5 and thereby potentially interferes with its GTPase activator protein function [39].

As the eIF 1, 5 and 6 turned out to be the novel promising candidates in targeting CRC we investigated them in more detail in knockdown experiments. After successful silencing of *eIF1*, *eIF5* and *eIF6,* both the proliferation rate and the clonogenicity of HCT116 cells were significantly reduced. Apoptosis significantly increased later during treatment (72h). The silencing of *eIF1*, *eIF5* and *eIF6* resulted in a reduction of polysomes, indicating reduced overall translation.

## MATERIALS AND METHODS

### Tissue microarrays (TMAs)

Tumor material was obtained with informed consent from 40 CRC patients with clinical and pathological data (Table 1) at the Medical University of Graz and the St. John of God Hospital Graz under approval from the ethics committee of the Medical University of Graz and the ethics committee of the St. John of God Hospital Graz (23-015 ex 10/11).

Kaplan-Meier curves were generated using the survival R package. The log rank test was applied to test for association of survival and gene expression. A p value <0.05 was considered as statistically significant. To identify the association between gene expressions, stratified by median, and survival, The Cancer Genome Atlas (TCGA) public dataset including 201 and 70 subjects suffering from colon adenocarcinoma and rectum carcinoma, respectively, was analyzed.

Tumor staging was reviewed by an experienced, board-certified pathologist (J. H.) using haematoxylin and eosin stained sections and relevant tumor areas were marked on the slide. Tissue cores of 1.2 mm in diameter were punched out from the chosen tumor area and embedded as an array in a fresh paraffin block. Tissue sections were cut at 4 μm and mounted on adhesive-coated glass slides compatible for immunohistochemical staining and analysis.

Seventeen patient-derived tumors from liver metastases of primary CC and RC were used to generate liver metastasis TMA (LM TMA). It was generated from 11 CC-Met (27% female; 73% male) and 6 RC-Met patients (100% male) and non-neoplastic liver tissue (NNLT) with a total of 185 spots.

Twenty-one samples from primary CC (16 low grade and 5 high grade primary CCs), 24 samples from primary RC (14 low grade and 10 high grade primary RCs), 16 samples of LM from primary CC and RC patients (9 CC-Met and 7 RC-Met), 19 samples of NNT (9 CC and 10 RC healthy tissues samples) and 14 NNLT (5 non-neoplastic liver tissue from CC-Met and 4 non-neoplastic liver tissues from RC-Met samples) served as healthy controls for immunoblot and RT-PCR.

Tumor type and grade were histologically diagnosed according to the current WHO classification (Hamilton and Aaltonen, 2000), the tumor stage according to UICC.

### Immunohistochemistry (IHC)

IHC was performed on a Ventana Immunostainer XT (Ventana Medical Systems, Tucson, USA), using an ultra-VIEW universal DAB Detection Kit (Ventana Medical Systems, Tucson, USA) and cell conditioning solution for 30 minutes using heat induced epitope retrieval (HIER). The primary antibodies were incubated for 30 minutes using different dilutions (Supplementary Table 6).

Two independent observers (N. GS., J. H.), blinded to the clinical data relating to the respective cases, used light microscopy for scoring. eIF expression was evaluated with respect to staining intensity (intensity score 0-3; 0 no staining, 1 weak, 2 moderate and 3 strong) and percentage of positive cells (proportion score; 0-100%).

### *In situ* detection using padlock probes

Tissues were deparaffinized, permeabilized with pepsin and subjected to in situ reactions. In situ detection of eIF1, eIF5 and eIF6 detected in situ using a multiplexed reaction.

All oligonucleotides were designed using CLC Main Workbench software (CLC Bio Workbench Version 7.6, Qiagen; Venlo, Netherlands). mRNA sequences were retrieved from the National Center for Biotechnology Information (NCBI) with the GenBank accession numbers NM_005801(eIF1), NM_001969 (eIF5) and NM_002212 (eIF6). The padlock probes were designed and ordered 5´ phosphorylated (Integrated DNA Technologies; Coralville, IA, USA). The primers were purchased from IDT DNA, detection oligos (Biomers; Ulm, Germany). Primer-, padlock probe- and detection oligo- sequences are shown in Supplementary Table 1.

Imaging was performed using a Zeiss Observer. Z1 inverted microscope (Carl Zeiss; Oberkochen, Germany) with a 20x objective and the ZEN 2.3 software (Carl Zeiss, blue edition, Version 2.3.64.0). Z-Stacks were projected into one layer by a maximum intensity projection with ZEN 2012 black software (Carl Zeiss, Version 8.1). For a better visualization, the brightness and contrast of images were adjusted with ZEN 2012 black software (Carl Zeiss). CellProfiler software (Version 2.1.1) was used for the quantification of signals. The modification includes a background correction, removing fluorescent signals which were detectable in at least two fluorescent channels simultaneously as this indicates unspecificity. Statistical analyses were performed using the GraphPad Prism software, version 6.01 (GraphPad Prism, Inc., La Jolla, USA). An unpaired t-test was applied to compare cancer vs. NNT in every group (colon and rectum). Results were considered statistically significant when p < 0.05.

### Protein extraction and immunoblot

All tumor tissue samples were acquired during surgery, immediately frozen in liquid nitrogen and stored at -80°C.

Frozen tissue samples were homogenized with a MagNA Lyser homogenizer (Roche Diagnostics, Risch-Rotkreuz, Switzerland) and lysed in NP-40 Lysis buffer (0.05 M Tris-HCl, 5 mM NaCl, 0.5% NP-40, 0.1 mM Pefabloc, 1 mM DTT, complete Mini, PhosSTOP). siRNA infected cells were scraped off into phosphate buffered saline (PBS) and lysed. The protein concentration was determined using Bradford protein assay (Biorad Protein Assay Dye Reagent, 500-0006; BioRad Laboratories GmbH, Munich, Germany). Equal amounts of 30 μg protein were loaded onto SDS-PAGE gels (30% Acrylamid/ Bisacrylamid solution; ROTH, Karlsruhe, Germany), subjected to electrophoresis in Mini-vertical electrophoresis units (Hoefer Inc, Richmond, USA) and blotted onto PVDF membranes (Immobilin-P Transfer Membrane; Millipore, Massachusetts, USA) using a Semi Dry Blotting Unit (SCIE-PLAS; Cambridge, England). The membranes were blocked in TBS tween (TBST) with 5% non-fat milk (AppliChem; Darmstadt, Germany) for 1h at room temperature. The primary antibodies (Supplementary Table 7) were diluted in TBST, 5% BSA and applied overnight at 4°C. The membranes were washed with TBST, followed by incubation with a horseradish peroxidase conjugated secondary antibody (anti-mouse 1:3000 and anti-rabbit 1:5000; GE Healthcare Life Sciences, Buckinghamshire, England). Proteins were visualized using a chemiluminescence ECL kit (GE Healthcare Life Sciences), followed by exposure on the Image Quant LAS 500 (GE Healthcare, Little Chalfont, UK). The signal was normalized using anti-β-actin antibody (mAb dilution 1:2000, Sigma-Aldrich, Missouri, USA). Three independent experiments were carried out.

### Quantitative real-time PCR

Total RNA was isolated from snap-frozen human primary CRC, metastases, NNT and NNLT using Trizol Reagent (Life Technologies; Woolston, UK), followed by extraction with phenol-chloroform. siRNA infected cells were washed three times with PBS, scraped off into PBS and lysed with Trizol Reagent. qRT-PCR was performed using the High-Capacity cDNA Reverse Transcription Kit (Applied Biosystems, FosterCity, USA) according to the manufacturer´s instructions and the GeneAmp 9700 Thermocycler (Applied Biosystems; Foster City, USA). Primers and dilutions used to determine the expression of different eIFs are shown in Supplementary Table 8. For the qRT-PCR the Power SYBR Green PCR Master Mix Kit (Applied Biosystems, Foster City, USA) was used in a 7900HT Fast Real-Time PCR System (Applied Biosystems, Foster City, USA). β-actin was used as housekeeping gene and the relative gene expression levels were calculated using the 2^ΔΔCT^ analysis method. Three independent experiments were carried out.

### Cell culture

The HCT116 cell line was obtained from the American Type Culture Collection (ATCC) and maintained in McCoy 5A medium supplemented with 10% fetal bovine serum (FBS) and penicillin/ streptomycin (100 μg/ ml), and incubated in a humidified atmosphere of 5% CO_2_ at 37°C. The HT29 cell line was kindly provided by Cpo – cellular phenomics & oncology Berlin-Buch GmbH and maintained in DMEM medium supplemented with 10% FBS and penicillin/ streptomycin (100 μg/ ml), and incubated in a humidified atmosphere of 5% CO_2_ at 37°C.

### siRNA transfection

We targeted the gene of interest by using small interfering RNAs (siRNAs) from QIAGEN (Hilden, Germany). For each gene of interest, two target sequences were used. For eIF1; 5´-GACCAGACATATCCTAG CTAA-3´and 5´-AAGCAATACCGTCATGTTTCA-3, for eIF5; 5´-AGGCGCTTAATCGGCCTCCAA-3´ and 5´-CA GCCAGAAGTGCAACATGTA-3´; for eIF6; 5´-CTGCT TTGCCAAGCTCACCAA-3´and 5´-CTGGTGCATCC CAAGACTTCA-3´.

Transfection experiments were performed using Metafectene^R^si^+^transfection reagent (Biontex, Munich, Germany) according to the manufacturer´s instructions. For the transfection, 1x SI buffer, Metafectene SI+ and siRNA were mixed into a drop. After an incubation of 15 min at room temperature 500 μl cells (80 000 cells/ well) were seeded onto a 24-well plate. Cells with transfection mix were cultured at 37°C in a humidified atmosphere of 5% CO_2_. Cells were collected after incubation for 24h, 48h and 72h. Three independent experiments were carried out.

### Proliferation assay

Transfected cells and control were seeded in 96-well plates (80 000 cells/ well) and cultivated under low serum conditions (1% FBS) for 24h, 48h and 72h. Viable cell number was determined on the basis of mitochondrial conversion of 3-(4,5-dimethylthiazol-2-yl)-2,5-diphenyltetrazolium bromide (MTT, Sigma Aldrich, Missouri, USA) to formazine. Cells were incubated with MTT for 2h at 37 °C, the medium supernatant was removed and cells were lysed with sodium dodecyl sulphate for 15min at room temperature. The MTT formazan crystals were dissolved with isopropanol/ HCl under shaking for 15 min at room temperature. Optical density was measured at 570 nm (Synergy^TM^4, BioTek, Winooski, USA). Each assay was executed in six-fold determination and three independent experiments were performed.

### Apoptosis

Apoptotic cells were detected using YO-PRO®-1 (Thermo Fisher Scientific, Massachusetts, USA) reagent. siRNA-transfected and control cells were seeded onto 96-well plates (80 000 cells/ well). After 24h, 48h and 72h, cells were incubated with YO-PRO®-1 for 15min at 37°C, the supernatant was removed, cells were washed with PBS and then measured at 485 nm to 535 nm. Each assay was performed in six-fold determination and three independent experiments were carried out.

### Invasion assay

For analysis of invasiveness of CRC cells, the CytoSelect TM 24-Well Cell Invasion Assay (Cell Biolabs, San Diego, USA) was performed according to the manufacturer's instructions. 1×10^5^ siRNA transfected cells and control cells were suspended in medium with 10% FBS, placed in the upper chamber and incubated for 48h at 37°C. The cells that had invaded to the lower surface of the filter inserts were stained with crystal violet. The optical density was measured at 560 nm (Synergy^TM^4, BioTek, Winooski, USA).

### Colony forming assay

HCT116 cells transfected with eIF1, eIF5 and eIF6 siRNA and scrambled siRNA as control were collected and seeded in six-well plates at a density of 500 cells/ well. The medium was changed every three days. After two weeks of culture, cells were washed three times with PBS and fixed in 4% paraformaldehyde (Sigma-Aldrich, Missouri, USA). Fixed cells were stained by adding freshly prepared diluted Giemsa solution (Sigma-Aldrich, Missouri, USA) for 20 min. Then the cells were rinsed with distilled water and colonies were analysed using a microscope (Nikon TMS – Inverted Microscope, Tokyo, Japan). Three independent experiments were carried out.

### Sucrose density gradient centrifugation

Sucrose density-gradient centrifugation was performed to analyze the cellular distribution of polysomes, 80S ribosomes and free 40S and 60S subunits. Cells were cultured in 100 mm dishes and transfected with siRNA and control for 24h, 48h and 72h. 15 minutes prior to lysis, cells were incubated with 100 μg/ ml cycloheximide (Sigma-Aldrich, Missouri, USA) to stall ribosomes on the mRNA strand. Lysis was performed on ice by washing cells in ice-cold PBS containing 100 μg/ ml cycloheximide followed by suspension in lysis buffer (20 mM HEPES pH 7.4, 15 mM MgCl_2_, 200mM KCl, 1% Triton X-100, 2mM DTT and 100 μg/ml cycloheximide), and nuclei were removed by centrifugation (14000g, 10 min, 4°C). The supernatant was layered onto 15%-40% sucrose gradients (50 mM NH4Cl, 50 mM Tris-acetate pH 7.0, 12 mM MgCl2, 100μg/ ml cycloheximide and freshly added 1mM DTT) and centrifuged in a SW41Ti rotor (Beckman, Villepinte, France) for 150 min at 160000 g, 4°C without breaking. Sucrose density gradient profiles were analysed via an ISCO density gradient analyser unit, which analyses and simultaneously blots ribosomal distribution measured by an UA-6 detector with 254 nm filter (Teledyne ISCO, Nebraska, USA).

All fractions were precipitated with trichloroacetic acid overnight at -20°C to concentrate proteins for gel electrophoresis.

### Statistical analysis

All experimental data are represented as means ± standard error of the mean (SEM) and were analyzed by descriptive statistics and Mann-Whitney-U-Test. Significance levels were set to p < 0.05. All statistical analyses and graphs were generated using GraphPad Prism 4.03 software (GraphPad software Inc., La Jolla, CA, USA).

## CONCLUSION

Our results emphasize the importance of separating CC and RC. We were able to characterize the differences in the expression pattern of mTOR members and eIFs between NNT and primary CC and RC as well as NNLT and liver metastasis derived from primary CC and RC. Additionally, low and high grade tumors should be differentiated not only because of their different prognosis, but also due to their distinct molecular profiles.

In the literature, CC and RC are frequently summarized under the term CRC, but our findings indicate a probable prognostic impact when separating low and high grade CC and RC.

## SUPPLEMENTARY MATERIALS FIGURES AND TABLES



## Abbreviations

AMPK: 5’ adenosine monophosphate-activated protein kinase
BSA: Bovine serum albumin
CC: Colon carcinoma
CC: Met Liver metastases of primary colon carcinoma
cDNA: copy Deoxyribo-nucleic acid
CRC: Colorectal carcinoma
Co2: Carbon dioxide
DMEM: Dulbecco modified eagle medium
DNA: Deoxyribo-nucleic acid
DTT: Dithiothreitol
4E-BP1: 4E-binding protein 1
eIF: Eukaryotic translation initiation factors
FBS: Fetal bovine serum
GAPDH: Glyceraldehyde 3-phosphate dehydrogenase
GTP: Guanosine triphosphate
HCl: Hydrochloric acid
HG: High grade
HIER: Heat induced epitope retrieval
IHC: Immunohistochemistry
LG: Low grade
LM: Liver metastasis
LM: TMA Liver metastasis tissue microarrays
mRNA: Messenger Ribonucleic acid
miRNA: Micro Ribonucleic acid
MTT: 3-(4,5-dimethylthiazol-2-yl)-2,5-diphenyltetrazolium bromide
mTOR: Mechanistic target of rapamycin
mTORC1: Mechanistic target of rapamycin complex 1
mTORC2: Mechanistic target of rapamycin complex 2
NaCl: Sodium chloride
NCBI: National Center for Biotechnology Information
NNCRM: Non-neoplastic colorectal mucosa
NNLT: Non-neoplastic liver tissue
NNT: Non-neoplastic colorectal mucosa tissue
PARP: Poly (ADP-ribose) polymerase
PBS: Phosphate buffered saline
PVDF: Polyvinylidene difluoride
pRT-PCR: Quantitative real-time polymerase chain reaction
RC: Rectum carcinoma
RC: Met Liver metastases of primary rectum carcinoma
RPL5: Ribosomal protein L5
RPS6: Ribosomal protein S6
RNA: Ribonucleic acid
SDS: Sodium dodecyl sulfate
SEM: Standard error of the mean
siRNA: Small interfering Ribonucleic acid
TBST: Tris buffered saline with Tween
TCGA: The Cancer Genome Atlas
TMA: Tissue microarrays
UICC: Union Internationale Contre le Cancer
WHO: World Health Organization
