# Correction: Separation of low and high grade colon and rectum carcinoma by eukaryotic translation initiation factors 1, 5 and 6

**DOI:** 10.18632/oncotarget.28115

**Published:** 2023-02-02

**Authors:** Nicole Golob-Schwarzl, Caroline Schweiger, Carina Koller, Stefanie Krassnig, Margit Gogg-Kamerer, Nadine Gantenbein, Anna M. Toeglhofer, Christina Wodlej, Helmut Bergler, Brigitte Pertschy, Stefan Uranitsch, Magdalena Holter, Amin El-Heliebi, Julia Fuchs, Andreas Punschart, Philipp Stiegler, Marlen Keil, Jens Hoffmann, David Henderson, Hans Lehrach, Christoph Reinhard, Christian Regenbrecht, Rudolf Schicho, Peter Fickert, Sigurd Lax, Johannes Haybaeck

**Affiliations:** ^1^Institute of Pathology, Medical University of Graz, Graz, Austria; ^2^Center for Biomarker Research in Medicine, Graz, Austria; ^3^Institute of Molecular Biosciences, Karl-Franzens-University of Graz, Graz, Austria; ^4^Department of Surgery, Hospital Brothers of Charity Graz, Graz, Austria; ^5^Institute of Medical Informatics, Statistics and Documentation, Medical University of Graz, Graz, Austria; ^6^Institute of Cell Biology, Histology and Embryology, Medical University Graz, Graz, Austria; ^7^Department of Surgery, Medical University of Graz, Graz, Austria; ^8^Experimental Pharmacology & Oncology Berlin GmbH-Berlin-Buch, Berlin, Germany; ^9^Bayer AG, Berlin, Germany; ^10^Max Planck Institute for Molecular Genetics, Berlin, Germany; ^11^Eli Lilly & Company, Indianapolis, USA; ^12^Cpo – cellular phenomics & oncology Berlin-Buch GmbH, Berlin, Germany; ^13^Institute of Experimental and Clinical Pharmacology, Medical University of Graz, Graz, Austria; ^14^Division of Gastroenterology and Hepatology, Medical University of Graz, Graz, Austria; ^15^Department of Pathology, Hospital Graz South-West, Austria; ^16^Department of Pathology, Otto-von-Guericke-University Magdeburg, Magdeburg, Germany

**This article has been corrected:** In Figure 4A, the image of the immunoblot for row ‘elF1’ in the CC column is an accidental duplicate of the ‘elF3B’ image in the ‘RC’ column of Figure 3A. The corrected Figure 4, produced using the original data, is shown below. The authors declare that these corrections do not change the results or conclusions of this paper.

Original article: Oncotarget. 2017; 8:101224–101243. 101224-101243. https://doi.org/10.18632/oncotarget.20642

**Figure 4:**
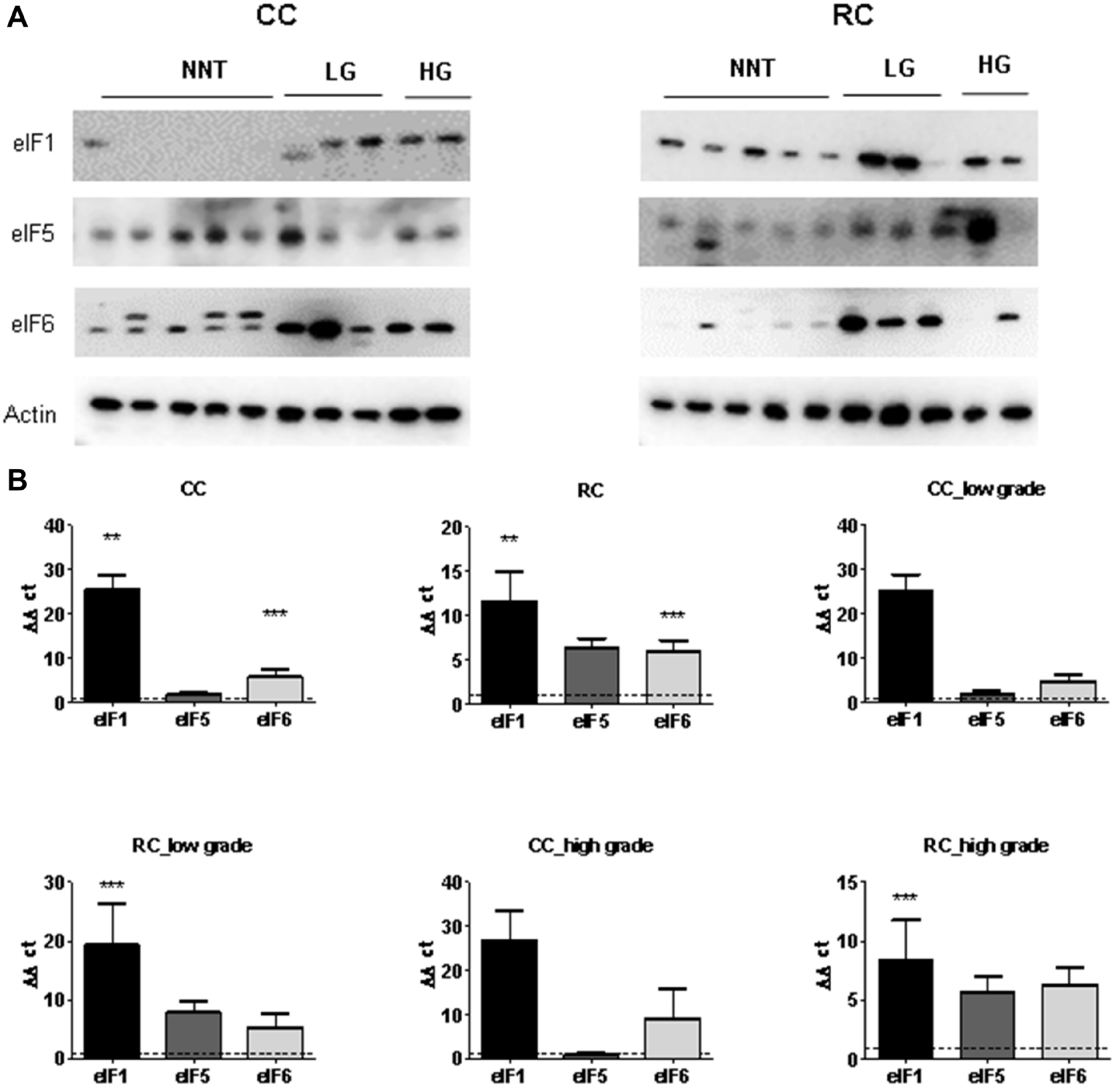
eIF1, eIF5 and eIF6 expression levels in low and high grade CC and RC. (**A**) Western blot of eIF1, eIF5 and eIF6 from LG and HG CC and RC NNT. Equal amounts of protein from each pair were resolved on SDS PAGE and immunoblotted with antibodies directed against eIF1, eIF5, eIF6 and β-actin (loading control). (**B**) qRT-PCR of eIF1, eIF5 and eIF6 from LG and HG CC and RC compared to NNT. Three independent experiments were carried out. Bars represent mean ± SEM. ^***^ *p* < 0.01, ^***^ *p* < 0.001. Statistical analysis: 2-way ANOVA with Bonferroni posttest.

