# Supplementary material for: Separation of low and high grade colon and rectum carcinoma by eukaryotic translation initiation factors 1, 5 and 6
Source: Oncotarget. 2017 Sep 5;8(60):101224–43. doi: 10.18632/oncotarget.20642 (PMC5731869; doi:10.18632/oncotarget.20642)
Supplement: Supplementary file 1 [file oncotarget-08-101224-s001.pdf]

Separation of low and high grade colon and rectum carcinoma by eukaryotic translation initiation factors 1, 5 and 6

SUPPLEMENTARY MATERIALS

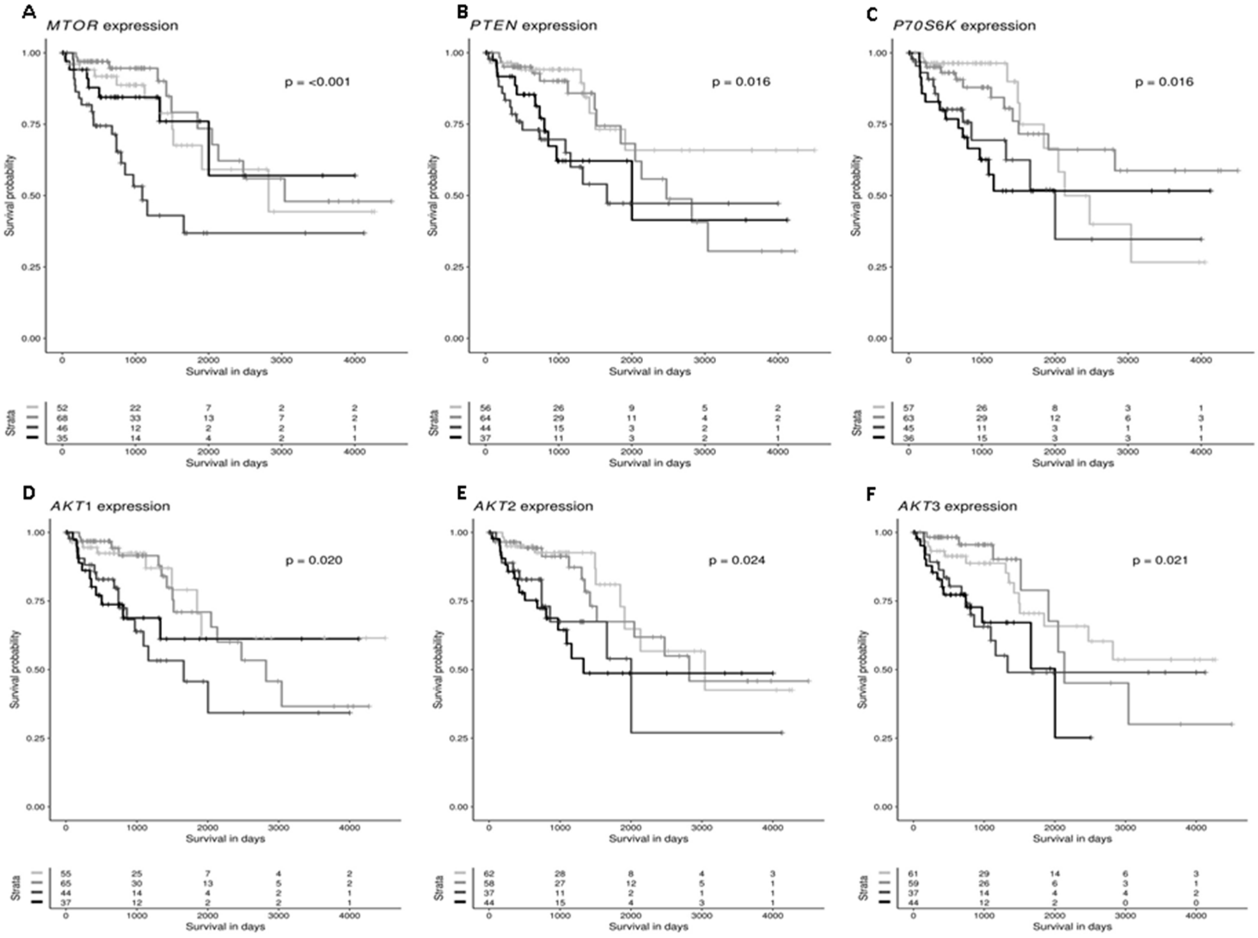

Supplementary Figure 1: mTOR, PTEN, p70S6K and AKT are clinically relevant candidates in colon carcinoma. Kaplan-Meier curves of mTOR, PTEN, p70S6K and AKT expression on overall survival for CC. Cases are divided in mTOR, PTEN, p70S6K and AKT low or high expressers according to whether expression is below or above median and survival are compared using the log-rank test. The light grey lines represent low tumor grade with low gene expression, medium grey lines display low tumor grade with high gene expression, the dark grey lines represent high tumor grade with low gene expression and the black lines high tumor grade with high gene expression. **(A)** mTOR dataset; **(B)** PTEN dataset; **(C)** p70S6K dataset; **(D)** AKT1 dataset; **(E)** AKT2 dataset and **(F)** AKT3 dataset.

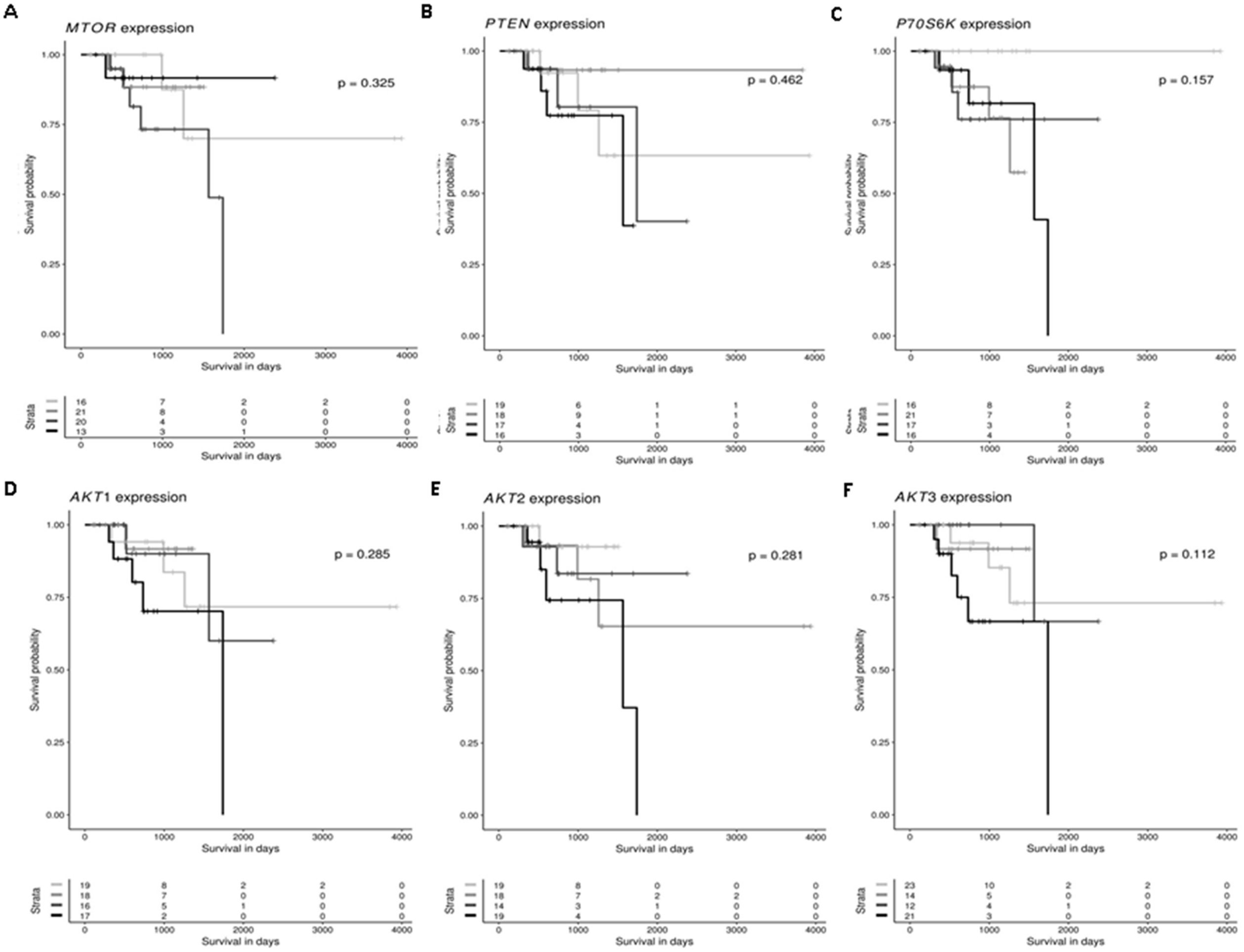

Supplementary Figure 2: mTOR, PTEN, p70S6K and AKT are clinically relevant candidates in rectum carcinoma. Kaplan-Meier curves of mTOR, PTEN, p70S6K and AKT expression on overall survival for RC. Cases are divided in mTOR, PTEN, p70S6K and AKT low or high expressers according to whether expression is below or above median and survival are compared using the log-rank test. The light grey lines represent low tumor grade with low gene expression, medium grey lines display low tumor grade with high gene expression, the dark grey lines represent high tumor grade with low gene expression and the black lines high tumor grade with high gene expression. **(A)** mTOR dataset; **(B)** PTEN dataset; **(C)** p70S6K dataset; **(D)** AKT1 dataset; **(E)** AKT2 dataset and **(F)** AKT3 dataset.

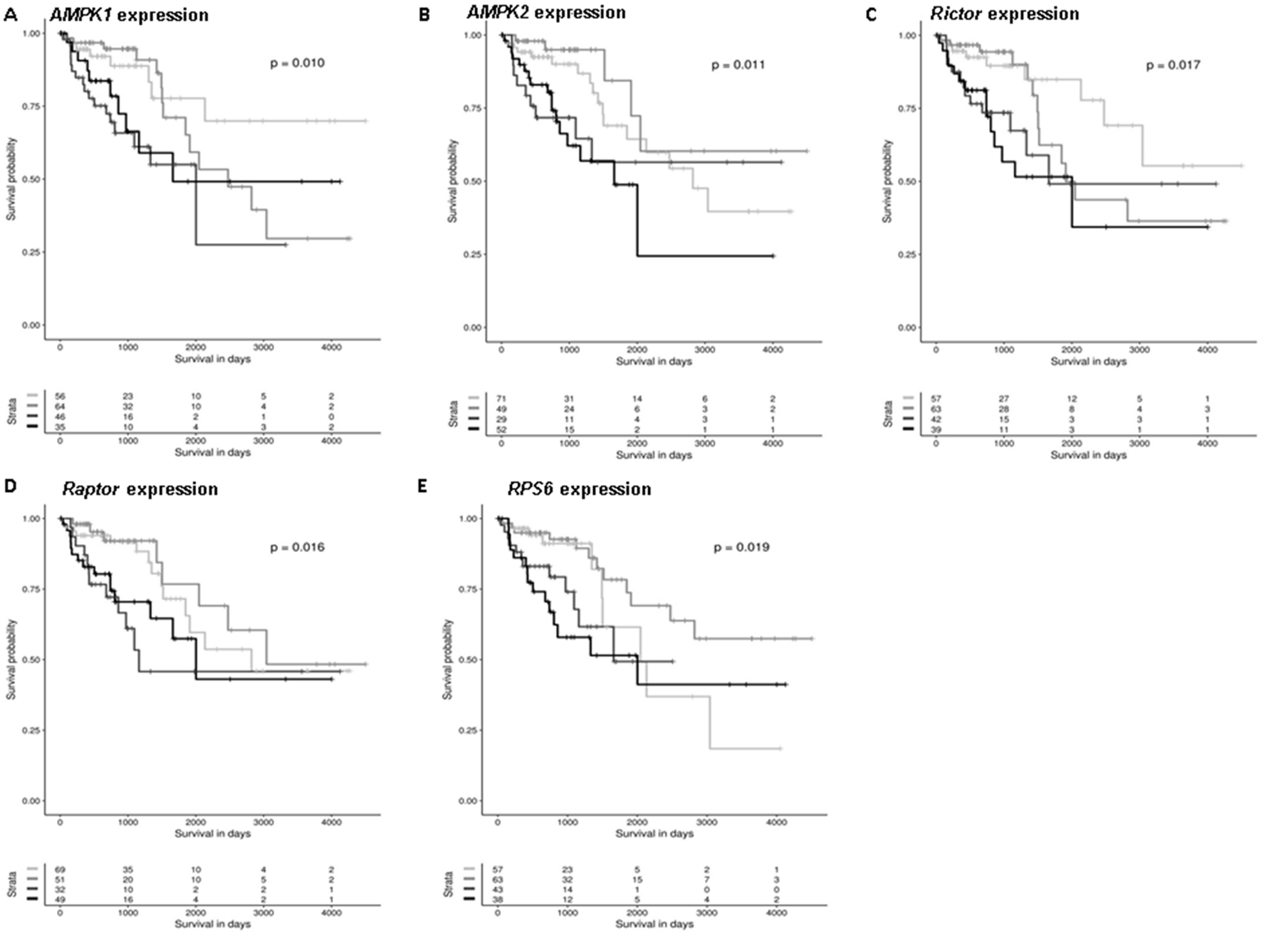

Supplementary Figure 3: AMPK1, AMPK2, Rictor, Raptor and RPS6 are clinically relevant candidates in colon carcinoma. Kaplan-Meier curves of AMPK1, AMPK2, Rictor, Raptor and RPS6 expression on overall survival for CC. Cases are divided PRKAA1, PRKAA2, Rictor, Raptor and RPS6 low or high expressers according to whether expression is below or above median and survival is compared using the log-rank test. The light grey lines represent low tumor grade with low gene expression, medium grey lines display low tumor grade with high gene expression, the dark grey lines represent high tumor grade with low gene expression and the black lines high tumor grade with high gene expression. **(A)** AMPK1 dataset; **(B)** AMPK2 dataset; **(C)** Rictor dataset; **(D)** Raptor dataset; and **(E)** RPS6 dataset.

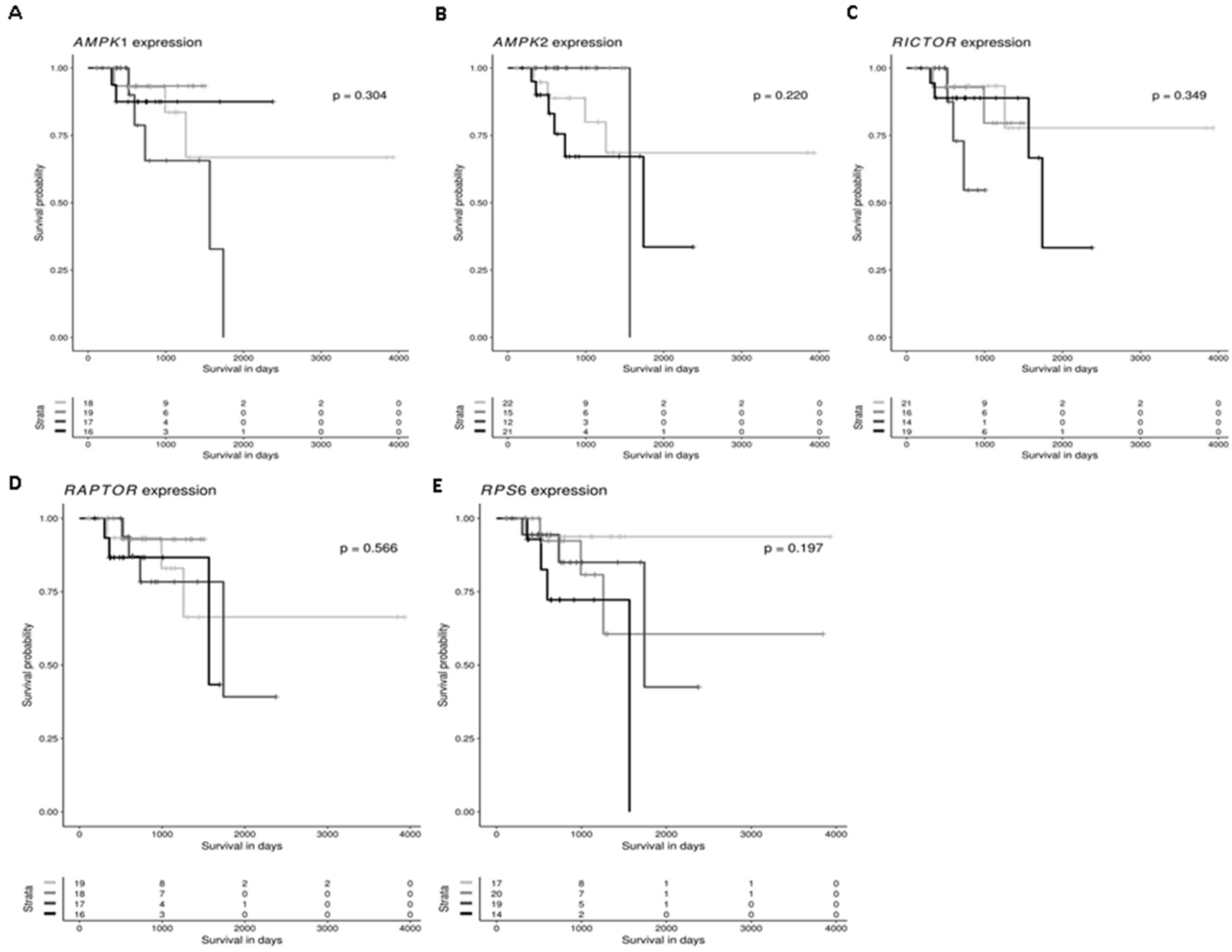

Supplementary Figure 4: AMPK1, AMPK2, Rictor, Raptor and RPS6 are clinically relevant candidates in rectum carcinoma. Kaplan-Meier curves of AMPK1, AMPK2, Rictor, Raptor and RPS6 expression on overall survival for RC. Cases are divided PRKAA1, PRKAA2, Rictor, Raptor and RPS6 low or high expressers according to whether expression is below or above median and survival is compared using the log-rank test. The light grey lines represent low tumor grade with low gene expression, medium grey lines display low tumor grade with high gene expression, the dark grey lines represent high tumor grade with low gene expression and the black lines high tumor grade with high gene expression. **(A)** AMPK1 dataset; **(B)** AMPK2 dataset; **(C)** Rictor dataset; **(D)** Raptor dataset; and **(E)** RPS6 dataset.

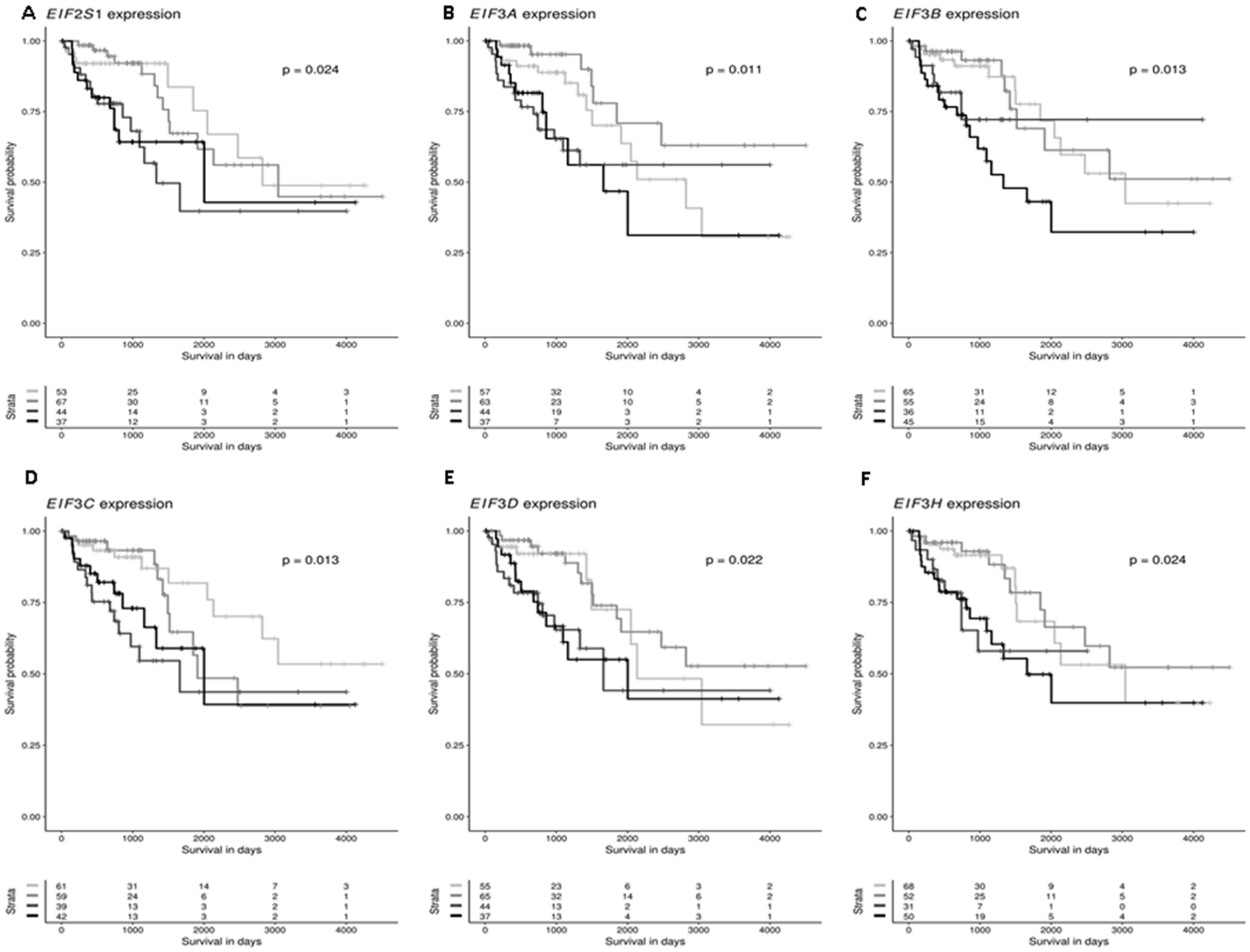

Supplementary Figure 5: eIF2S1, eIF3A, eIF3B, eIF3C, eIF3D and eIF3H are clinically relevant candidates in colon carcinoma. Kaplan-Meier curves of eIF2S1, eIF3A, eIF3B, eIF3C, eIF3D and eIF3H expression on overall survival for CC. Cases are divided in eIF2S1, eIF3A, eIF3B, eIF3C, eIF3D and eIF3Hlow or high expressers according to whether expression is below or above median and survival is compared using the log-rank test. The light grey lines represent low tumor grade with low gene expression, medium grey lines display low tumor grade with high gene expression, the dark grey lines represent high tumor grade with low gene expression and the black lines high tumor grade with high gene expression. **(A)** eIF2S1 dataset; **(B)** eIF3A dataset; **(C)** eIF3B dataset; **(D)** eIF3C dataset; **(E)** eIF3D dataset and **(F)** eIF3H dataset.

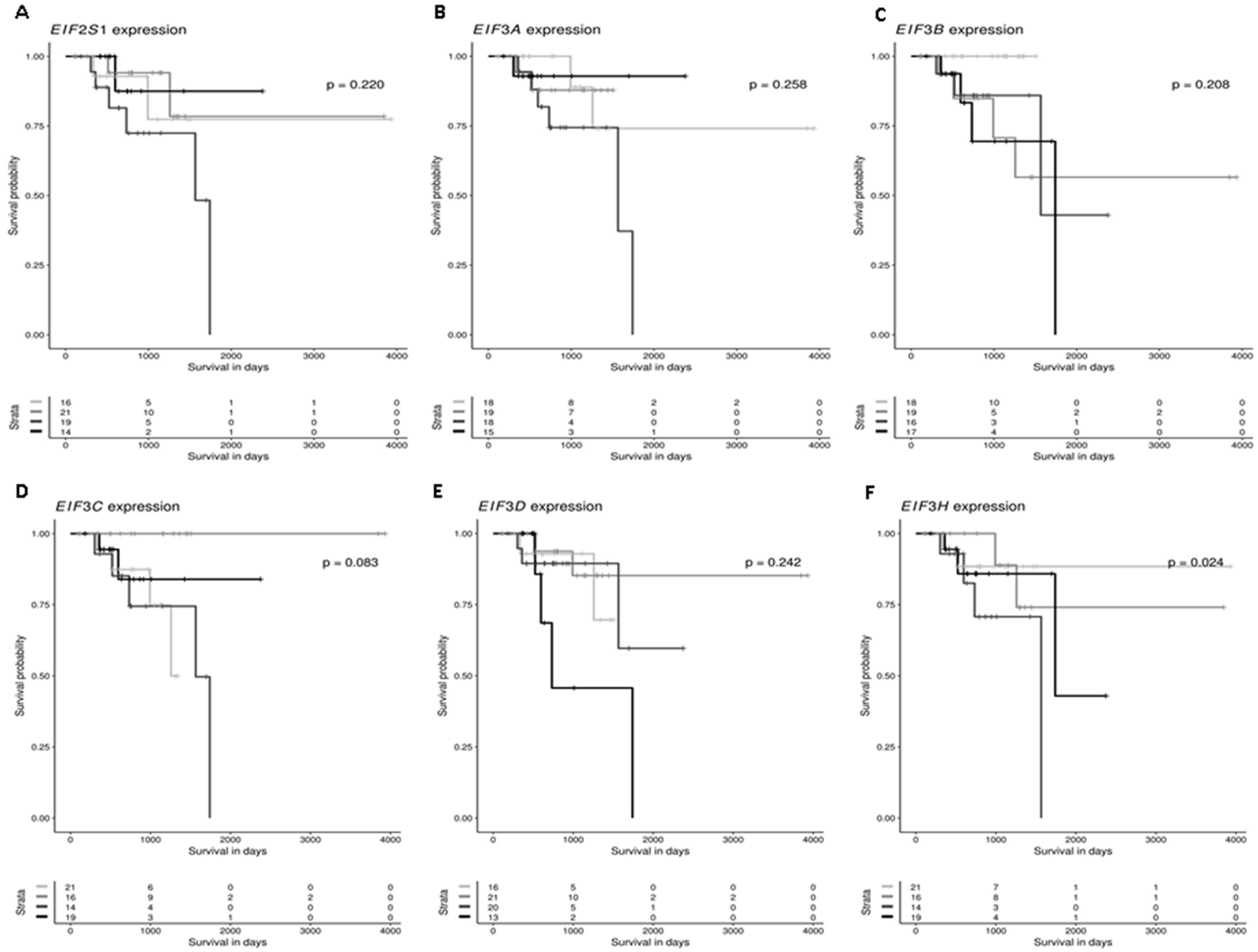

Supplementary Figure 6: eIF2S1, eIF3A, eIF3B, eIF3C, eIF3D and eIF3H are clinically relevant candidates in rectum carcinoma. Kaplan-Meier curves of eIF2S1, eIF3A, eIF3B, eIF3C, eIF3D and eIF3H expression on overall survival for RC. Cases are divided in eIF2S1, eIF3A, eIF3B, eIF3C, eIF3D and eIF3Hlow or high expressers according to whether expression is below or above median and survival is compared using the log-rank test. The light grey lines represent low tumor grade with low gene expression, medium grey lines display low tumor grade with high gene expression, the dark grey lines represent high tumor grade with low gene expression and the black lines high tumor grade with high gene expression. **(A)** eIF2S1 dataset; **(B)** eIF3A dataset; **(C)** eIF3B dataset; **(D)** eIF3C dataset; **(E)** eIF3D dataset and **(F)** eIF3H dataset.

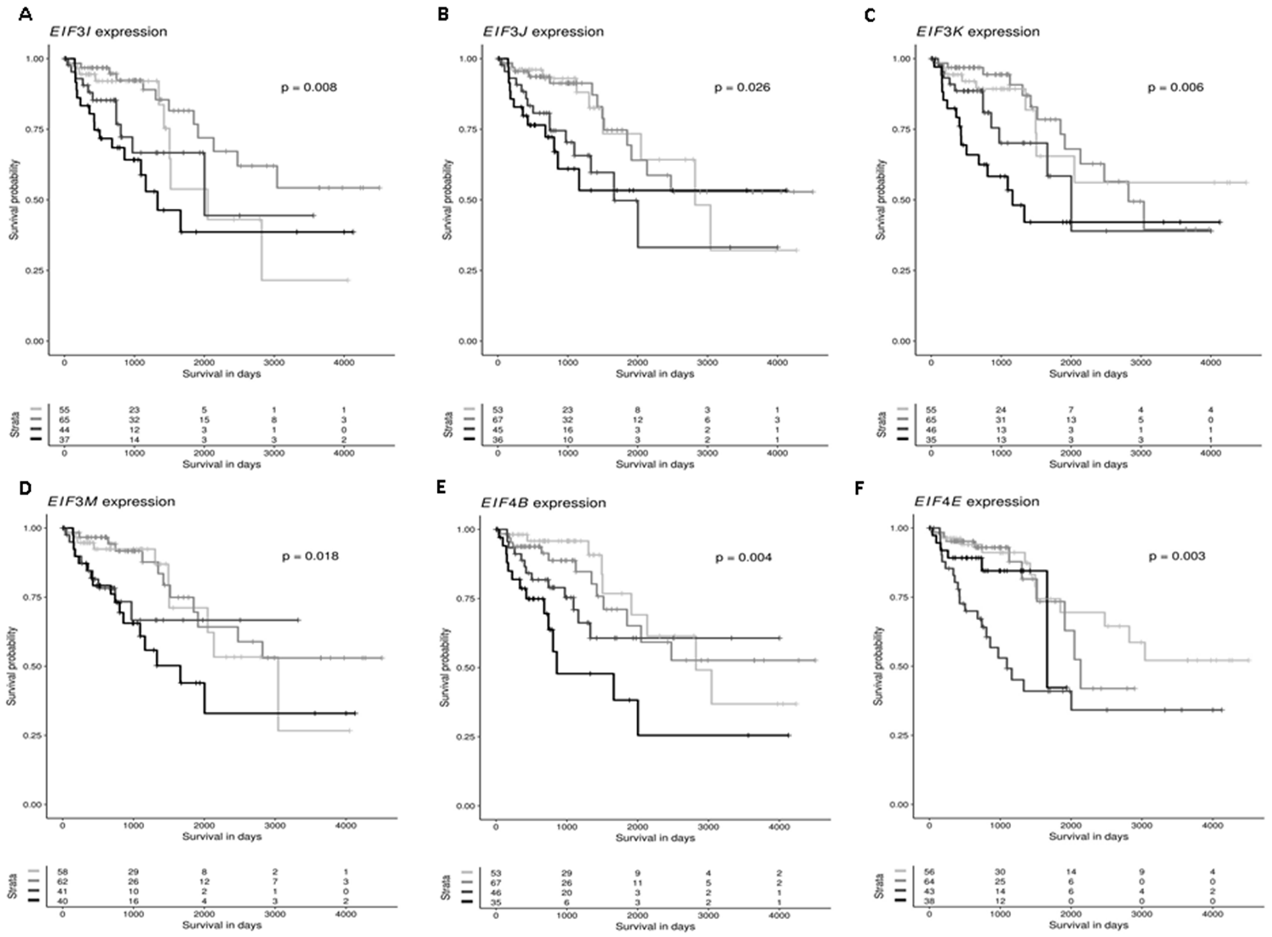

Supplementary Figure 7: eIF3I, eIF3J, eIF3K, eIF3M, eIF4B and eIF4E are clinically relevant candidates in colon carcinoma. Kaplan-Meier curves of eIF3I, eIF3J, eIF3K, eIF3M, eIF4B and eIF4E expression on overall survival for CC. Cases are divided in eIF3I, eIF3J, eIF3K, eIF3M, eIF4B and eIF4E low or high expressers according to whether expression is below or above median and survival is compared using the log-rank test. The light grey lines represent low tumor grade with low gene expression, medium grey lines display low tumor grade with high gene expression, the dark grey lines represent high tumor grade with low gene expression and the black lines high tumor grade with high gene expression. **(A)** eIF3I dataset; **(B)** eIF3J dataset; **(C)** eIF3K dataset; **(D)** eIF3M dataset; **(E)** eIF4B dataset and **(F)** eIF4E dataset.

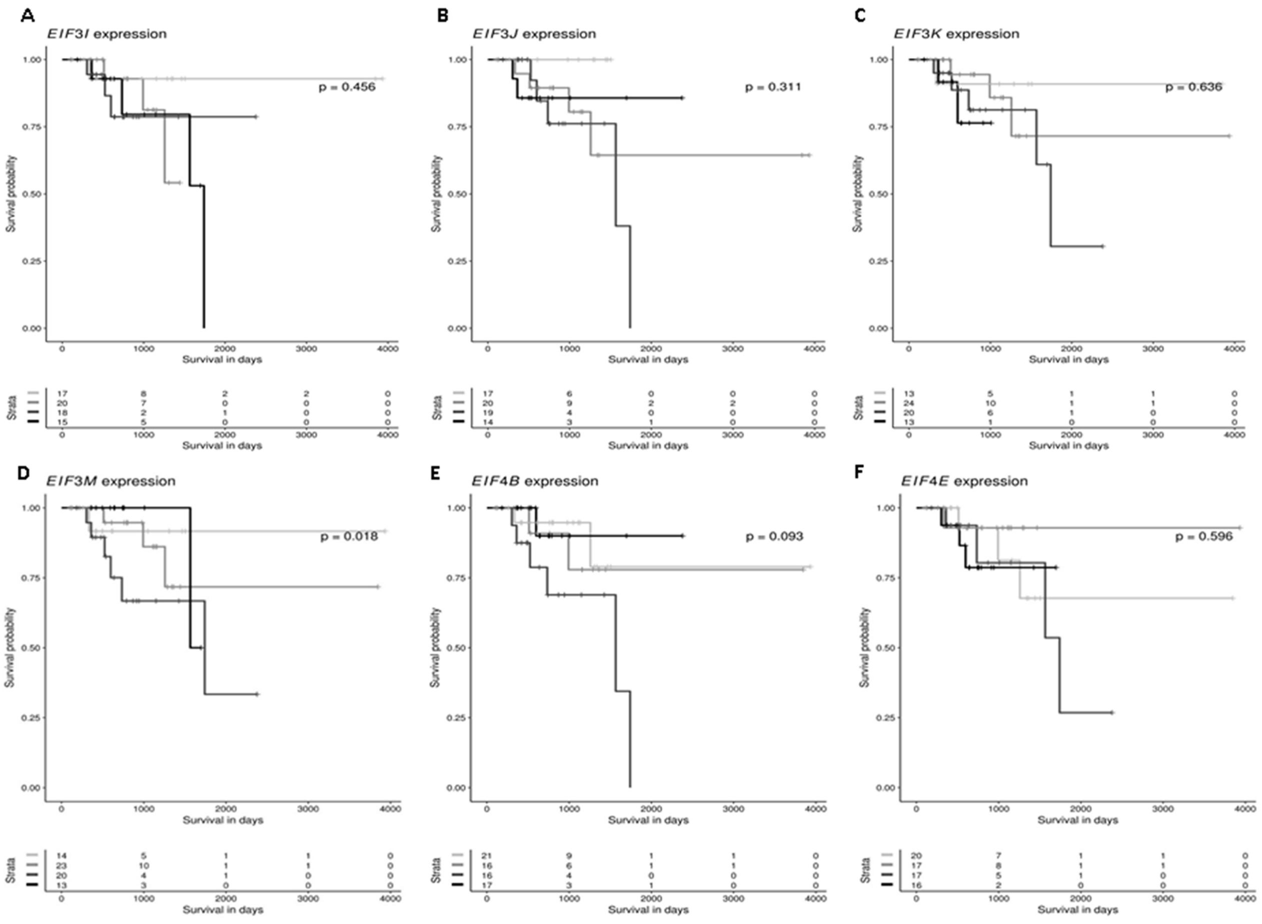

Supplementary Figure 8: eIF3I, eIF3J, eIF3K, eIF3M, eIF4B and eIF4E are clinically relevant candidates in rectum carcinoma. Kaplan-Meier curves of eIF3I, eIF3J, eIF3K, eIF3M, eIF4B and eIF4E expression on overall survival for RC. Cases are divided in eIF3I, eIF3J, eIF3K, eIF3M, eIF4B and eIF4E low or high expressers according to whether expression is below or above median and survival is compared using the log-rank test. The light grey lines represent low tumor grade with low gene expression, medium grey lines display low tumor grade with high gene expression, the dark grey lines represent high tumor grade with low gene expression and the black lines high tumor grade with high gene expression. **(A)** eIF3I dataset; **(B)** eIF3J dataset; **(C)** eIF3K dataset; **(D)** eIF3M dataset; **(E)** eIF4B dataset and **(F)** eIF4E dataset.

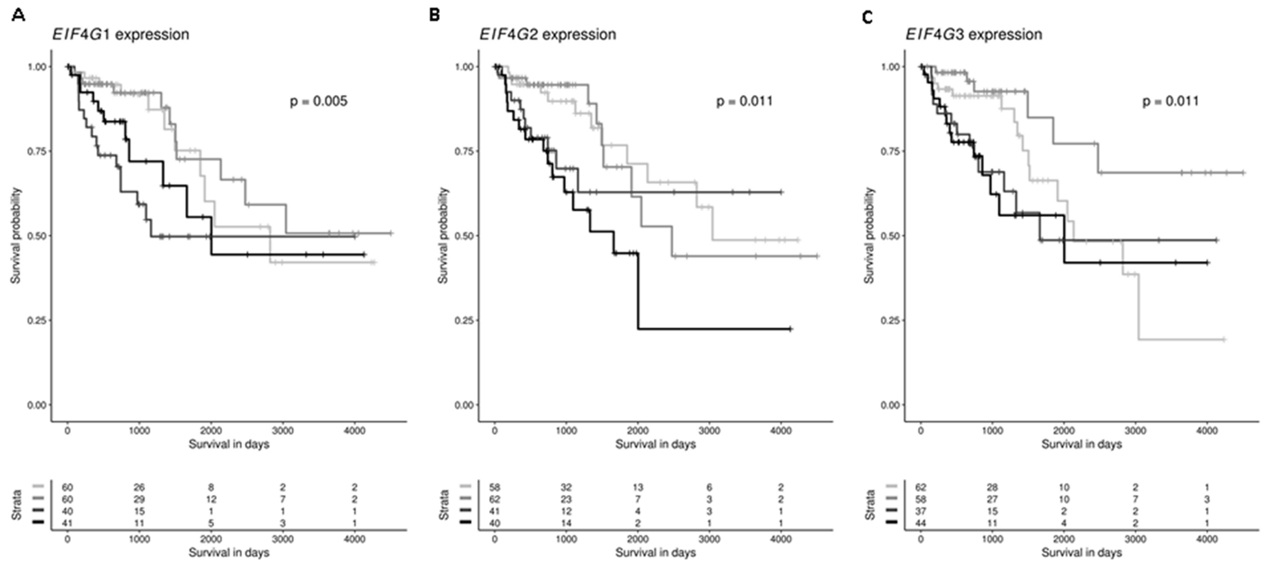

Supplementary Figure 9: eIF4G1, eIF4G2 and eIF4G3 are clinically relevant candidates in colon carcinoma. Kaplan-Meier curves of eIF4G1, eIF4G2 and eIF4G3 expression on overall survival for CC. Cases are divided in eIF4G1, eIF4G2 and eIF4G3 low or high expressers according to whether expression is below or above median and survival is compared using the log-rank test. The light grey lines represent low tumor grade with low gene expression, medium grey lines display low tumor grade with high gene expression, the dark grey lines represent high tumor grade with low gene expression and the black lines high tumor grade with high gene expression. **(A)** eIF4G1 dataset; **(B)** eIF4G2 dataset; **(C)** eIF4G3 dataset.

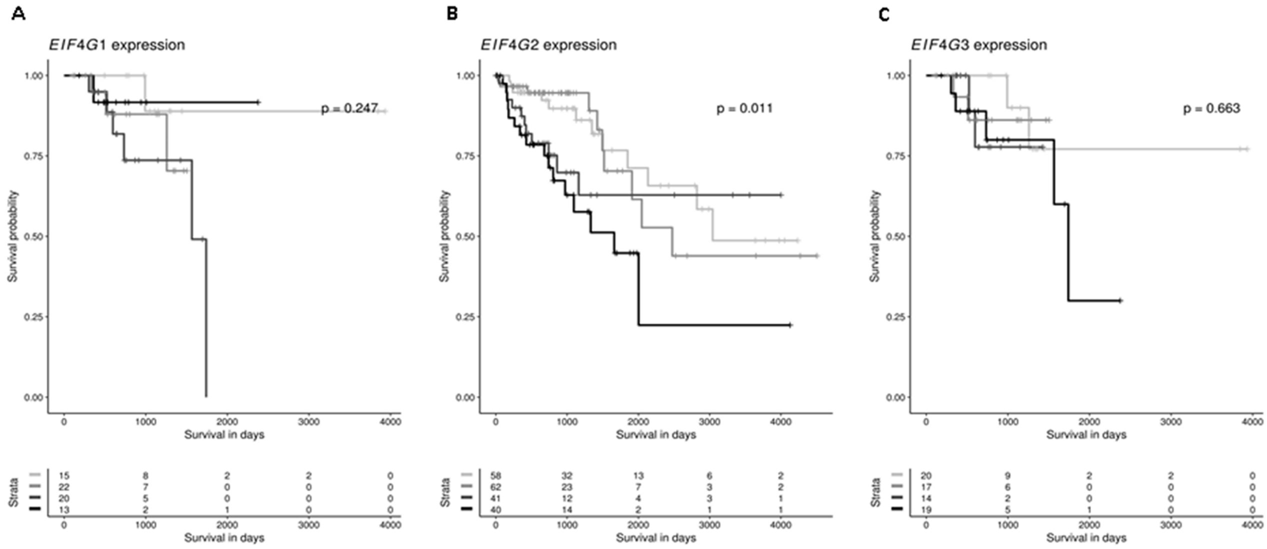

Supplementary Figure 10: eIF4G1, eIF4G2 and eIF4G3 are clinically relevant candidates in rectum carcinoma. Kaplan-Meier curves of eIF4G1, eIF4G2 and eIF4G3 expression on overall survival for RC. Cases are divided in eIF4G1, eIF4G2 and eIF4G3 low or high expressers according to whether expression is below or above median and survival is compared using the log-rank test. The light grey lines represent low tumor grade with low gene expression, medium grey lines display low tumor grade with high gene expression, the dark grey lines represent high tumor grade with low gene expression and the black lines high tumor grade with high gene expression. **(A)** eIF4G1 dataset; **(B)** eIF4G2 dataset; **(C)** eIF4G3 dataset.

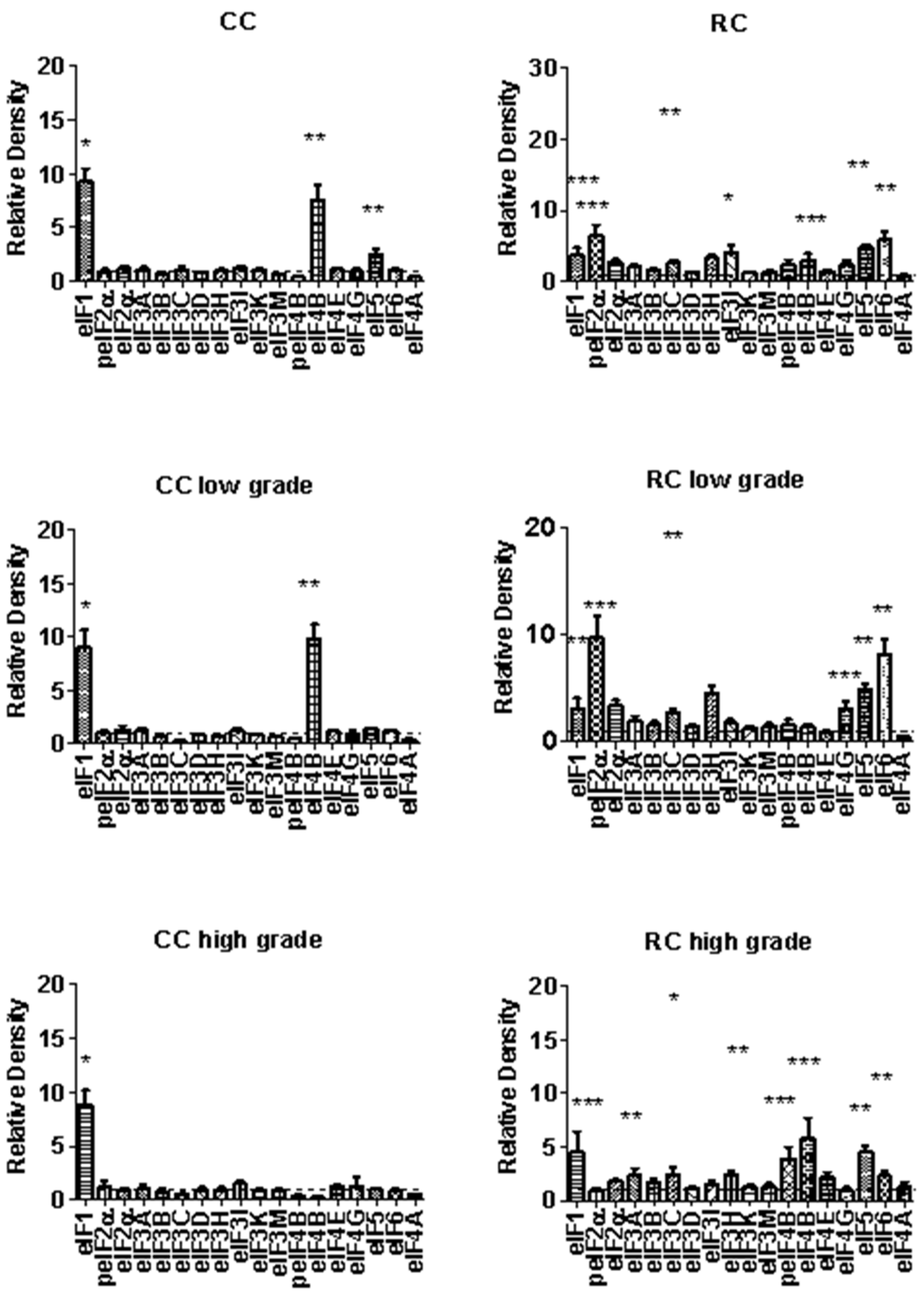

Supplementary Figure 11: eIF expression in level low and high grade colon and rectum carcinomas. Densitometric analyses of immunoblots from various eIF subunits and β-actin in low grade (LG) and high grade (HG) colon carcinomas (CC) and rectum carcinomas (RC) compared to non-neoplastic tissues (NNT). This figure is related to Figure 3. Three independent experiments were carried out. Bars represent mean ± SEM. $^{*}p < 0.05$, $^{**}p < 0.01$, $^{***}p < 0.001$. Statistical analysis: 2-way ANOVA with Bonferroni posttest.

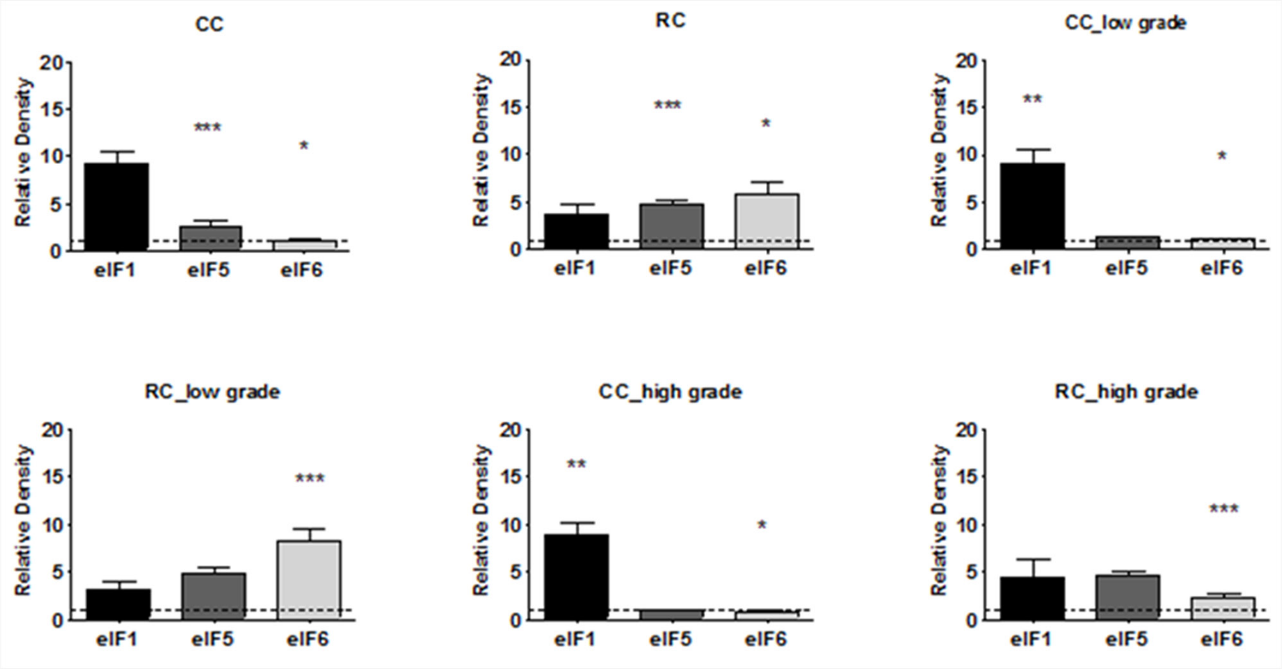

Supplementary Figure 12: eIF1, eIF5 and eIF6 expression levels in low and high grade colon and rectum carcinomas. Densitometric analyses of immunoblots from eIF1, eIF5 and eIF6 and β-actin in low grade (LG) and high grade (HG) colon carcinomas (CC) and rectum carcinomas (RC) compared to non-neoplastic tissues (NNT). This figure is related to Figure 4. Three independent experiments were carried out. Bars represent mean ± SEM. $^{*}p < 0.05$, $^{**}p < 0.01$, $^{***}p < 0.001$. Statistical analysis: 2-way ANOVA with Bonferroni posttest.

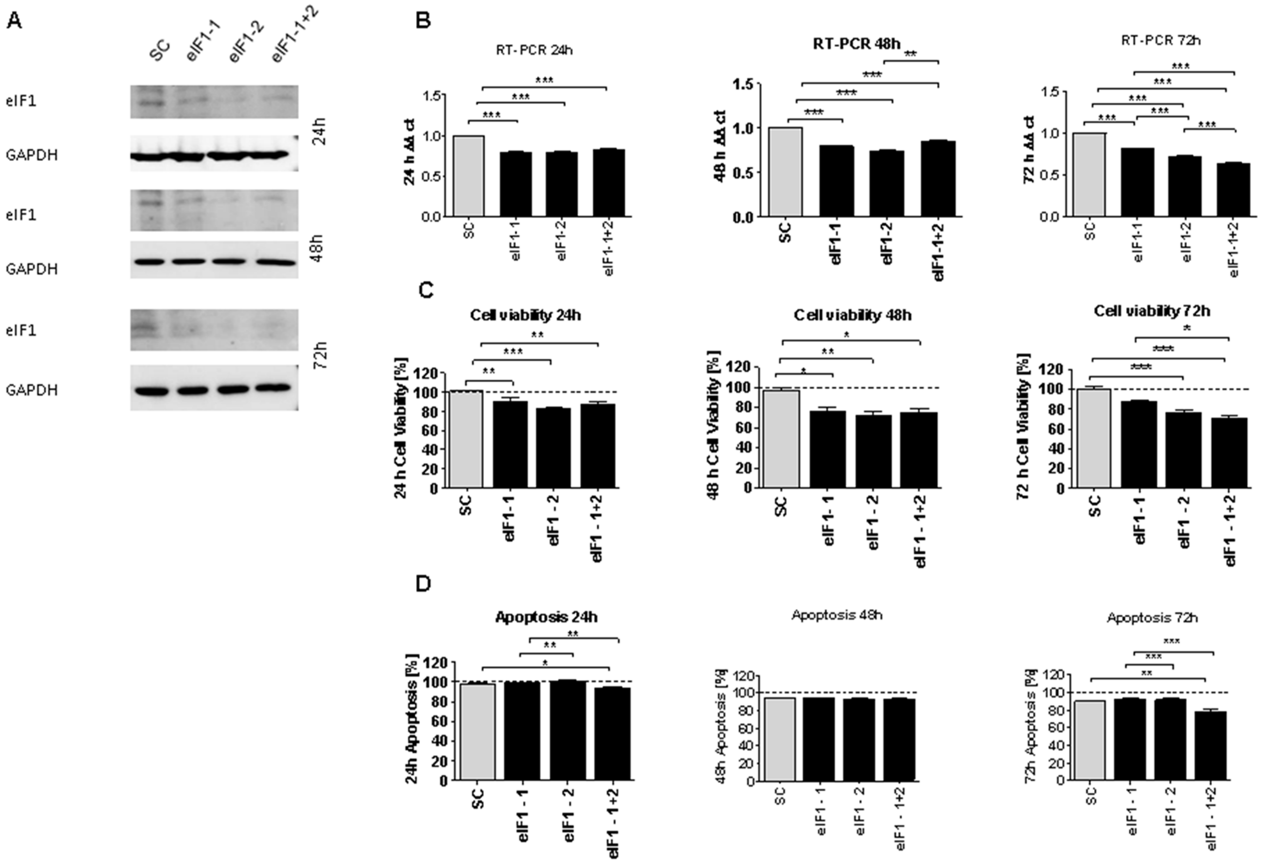

Supplementary Figure 13: *In vitro* characterization of the effect of eIF1 knockdown in HT29 cells. (A) Protein expression of *eIF1* siRNA constructs after 24h, 48h and 72h compared to SC. **(B)** The mRNA expression of *eIF1* in HT29 cells compared to the SC. **(C)** Cell viability after *eIF1* knockdown for 24h, 48h and 72h. **(D)** YO-PRO assay after *eIF1* knockdown for 24h, 48h and 72h in HT29 cells. Three independent experiments were carried out. Bars represent mean ± SEM. $^{*}p < 0.05$, $^{**}p < 0.01$, $^{***}p < 0.001$. Statistical analysis: 2-way ANOVA with Bonferroni posttest.

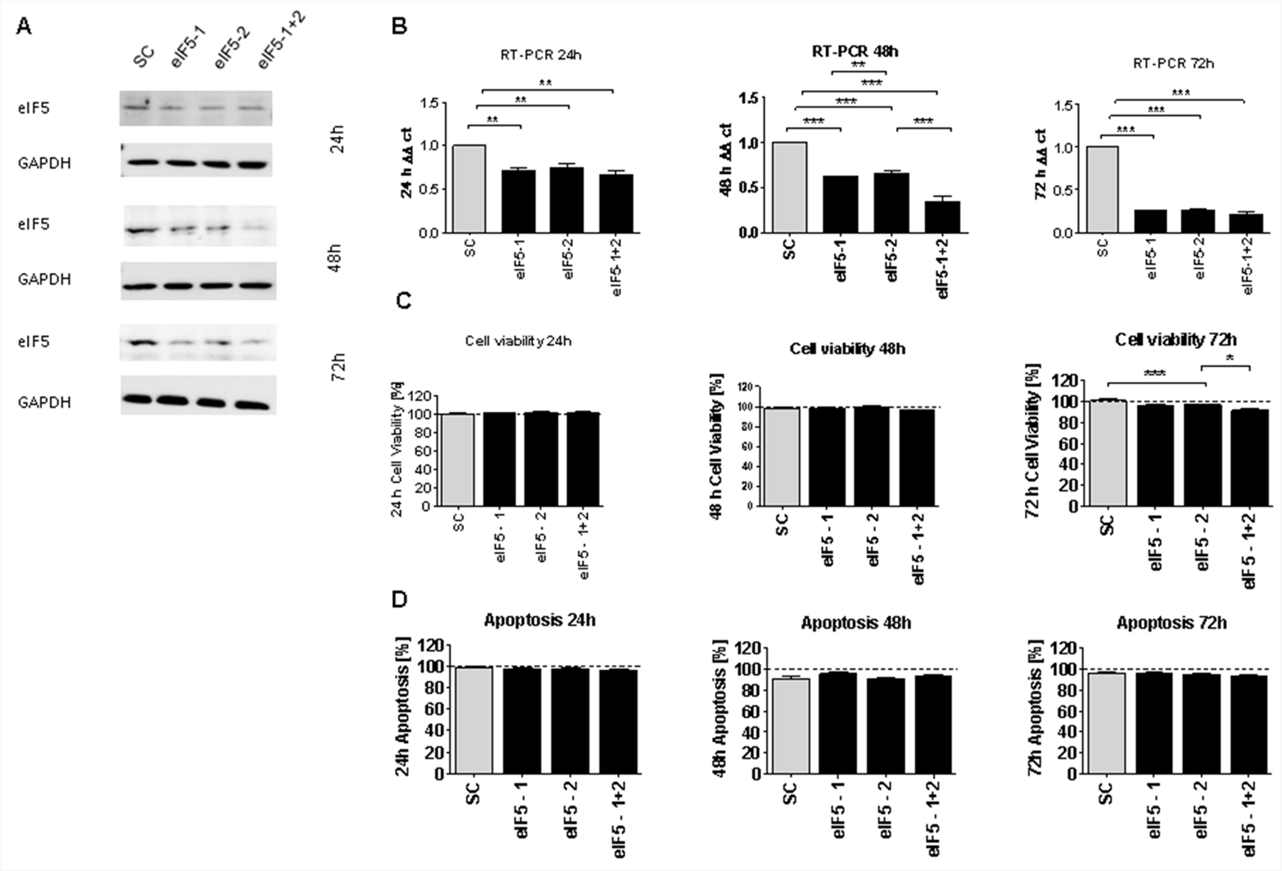

Supplementary Figure 14: *In vitro* characterization of the effect of eIF5 knockdown in HT29 cells. **(A)** Protein expression of *eIF5* siRNA constructs after 24h, 48h and 72h compared to SC. **(B)** The mRNA expression of *eIF5* in HT29 cells compared to SC. **(C)** Cell viability after *eIF5* knockdown for 24h, 48h and 72h. **(D)** YO-PRO assay after *eIF5* knockdown for 24h, 48h and 72h in HT29 cells. Three independent experiments were carried out. Bars represent mean ± SEM. $^{*}p < 0.05$, $^{**}p < 0.01$, $^{***}p < 0.001$. Statistical analysis: 2-way ANOVA with Bonferroni posttest.

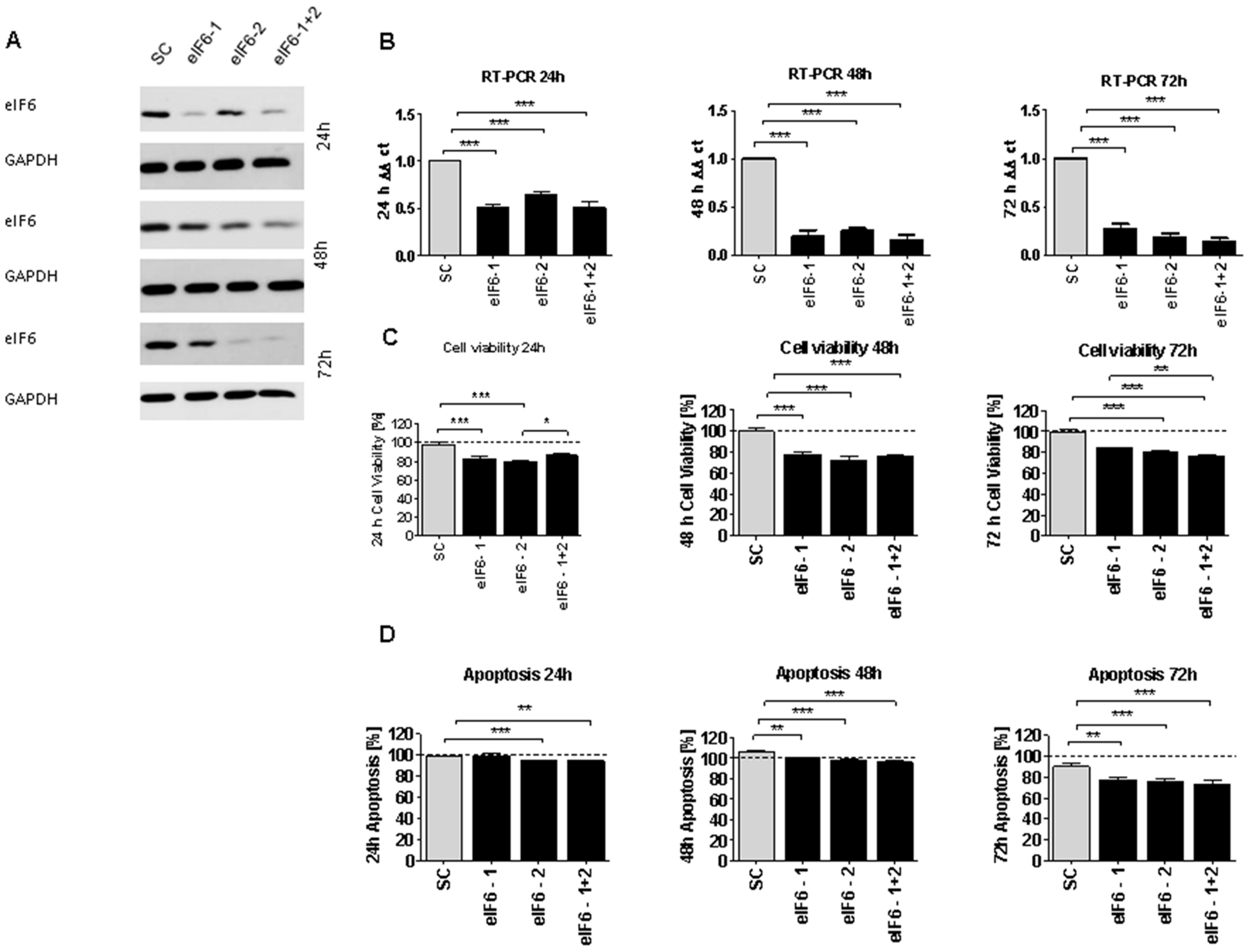

Supplementary Figure 15: *in vitro* characterization of the effect of eIF6 knockdown in HT29 cells. **(A)** Protein expression of *eIF6* siRNA constructs after 24h, 48h and 72h compared to SC. **(B)** The mRNA expression of *eIF6* in HT29 cells compared to SC. **(C)** Cell viability after *eIF6* knockdown for 24h, 48h and 72h. **(D)** YO-PRO assay after *eIF6* knockdown for 24h, 48h and 72h in HT29 cells. Three independent experiments were carried out. Bars represent mean ± SEM. $^{*}p < 0.05$, $^{**}p < 0.01$, $^{***}p < 0.001$. Statistical analysis: 2-way ANOVA with Bonferroni posttest.

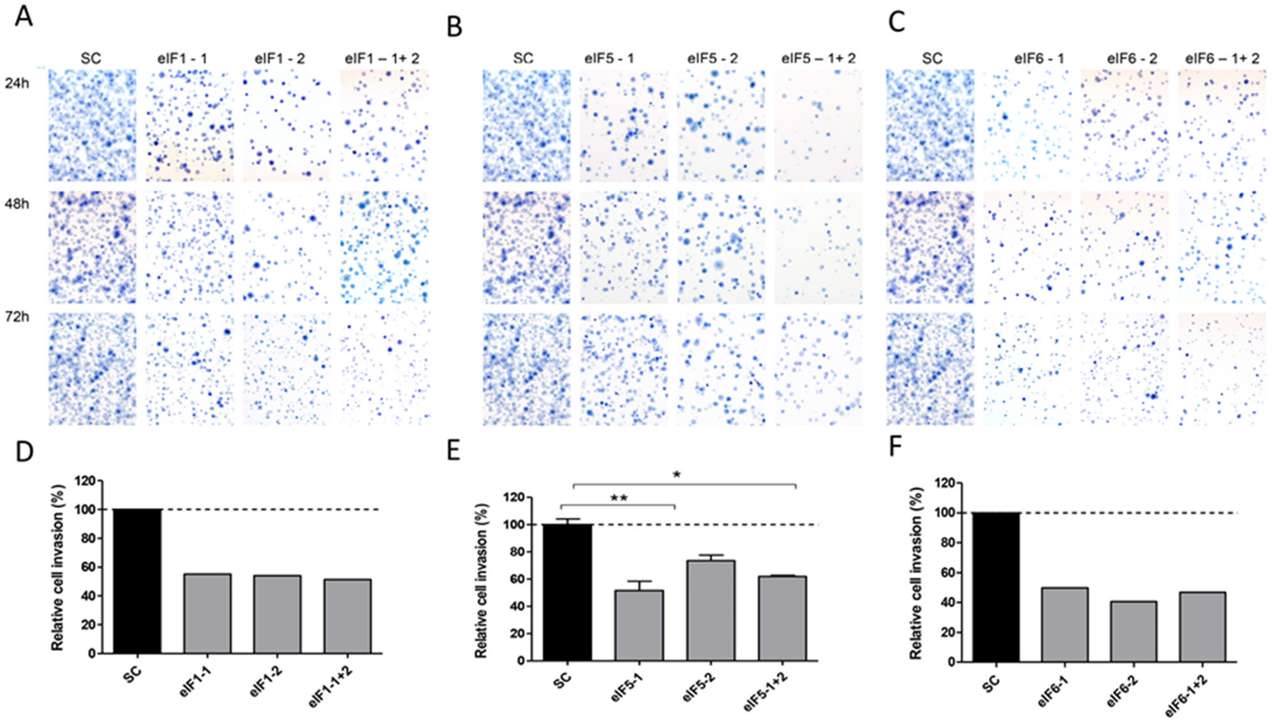

Supplementary Figure 16: *In vitro* characterization of the effect eIF1, eIF5 and eIF6 knockdown in HCT116 cells on invasiveness and clonogenicity. **(A)** Colony formation after eIF1 transfection in HCT116 cells after 21 days. **(B)** Colony formation after si-eIF5 transfection in HCT116 cells after 21 days. **(C)** Colony formation after si-eIF6 transfection in HCT116 cells after 21 days. **(D)** Invasive properties of HCT116 cells after eIF1 knockdown. **(E)** Invasive properties of HCT116 cells after si-eIF5. **(F)** Invasive properties of HCT116 cells after eIF6 knockdown. This figure is related to Figure 4, 5 and 6. Three independent experiments were carried out. Bars represent mean ± SEM. $^{*}p < 0.05$, $^{**}p < 0.01$, $^{***}p < 0.001$. Statistical analysis: 2-way ANOVA with Bonferroni posttest.

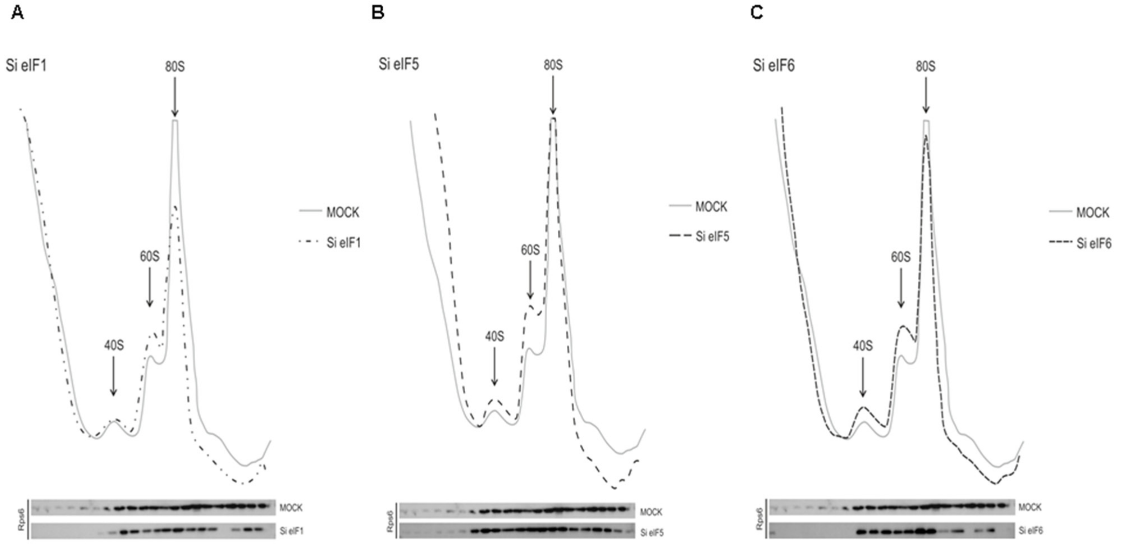

Supplementary Figure 17: Sucrose density gradient centrifugation of lysates from transfected HCT116 cells. Overlays of profiles from MOCK cells (continous lines) and eIF-silenced cells (dashed lines) are shown. Below the profiles, immunoblot analyses of the fractions are displayed. RPS6 was detected as a marker protein to estimate the distribution of the small ribosomal subunit in the gradient. **(A)** eIF1-silenced cells compared to MOCK. **(B)** eIF5-silenced cells compared to MOCK. **(C)** eIF6-silenced cells compared to MOCK.

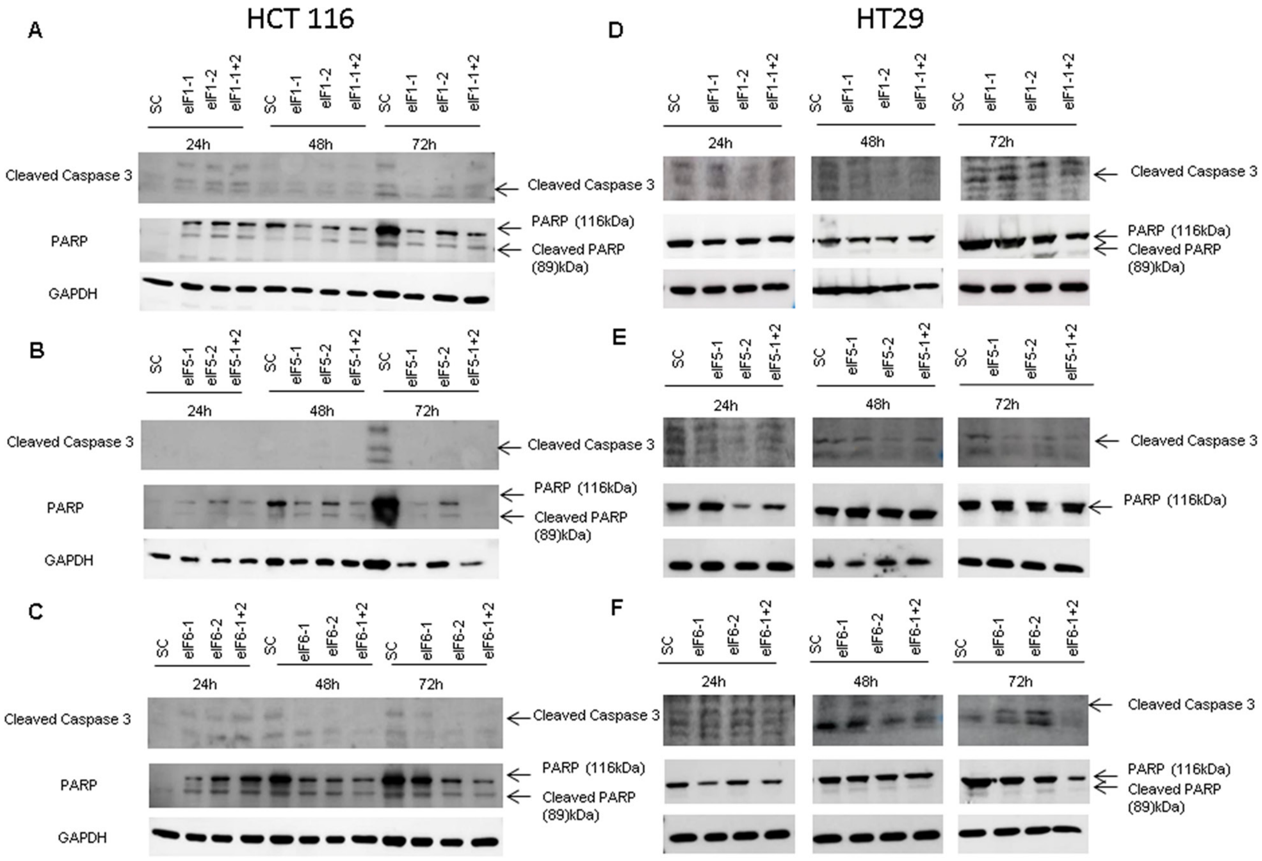

Supplementary Figure 18: *In vitro* characterization of the effect eIF1, eIF5 and eIF6 knockdown in HCT116 cells on apoptosis rate. **(A)** Protein expression of cleaved caspase 3 and PARP in eIF1-transfected HCT116 cells. **(B)** Protein expression of cleaved caspase 3 and PARP in eIF5- silenced HCT116 cells. **(C)** Protein expression of cleaved caspase 3 and PARP in eIF6-silenced HCT116 cells. **(D)** Protein expression of cleaved caspase 3 and PARP in eIF1- silenced HT29 cells. **(E)** Protein expression of cleaved caspase 3 and PARP in eIF5- silenced HT29 cells. **(F)** Protein expression of cleaved caspase 3 and PARP in eIF6- silenced HT29 cells. Three independent experiments were carried out. Bars represent mean ± SEM. $^{*}p < 0.05$, $^{**}p < 0.01$, $^{***}p < 0.001$. Statistical analysis: 2-way ANOVA with Bonferroni posttest.

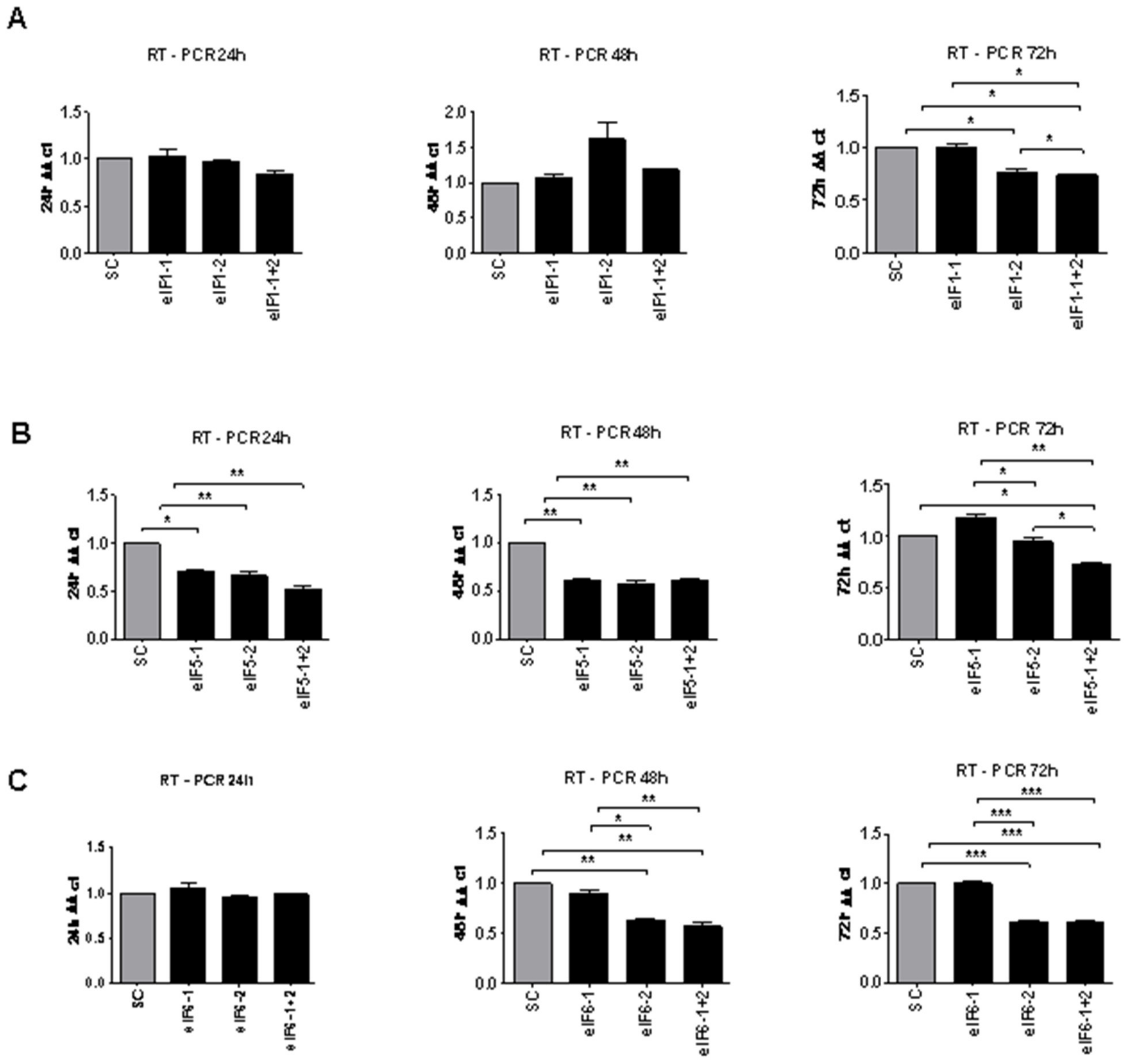

Supplementary Figure 19: *in vitro* characterization of the effect eIF1, eIF5 and eIF6 knockdown in HCT116 cells on PARP rate. **(A)** mRNA expression of PARP in eIF1- HCT116 cells. **(B)** mRNA expression of PARP in eIF5- HCT 116 cells. **(C)** mRNA expression of PARP in eIF6- HCT116 cells. Three independent experiments were carried out. Bars represent mean ± SEM. $^{*}p < 0.05$, $^{**}p < 0.01$, $^{***}p < 0.001$. Statistical analysis: 2-way ANOVA with Bonferroni posttest.

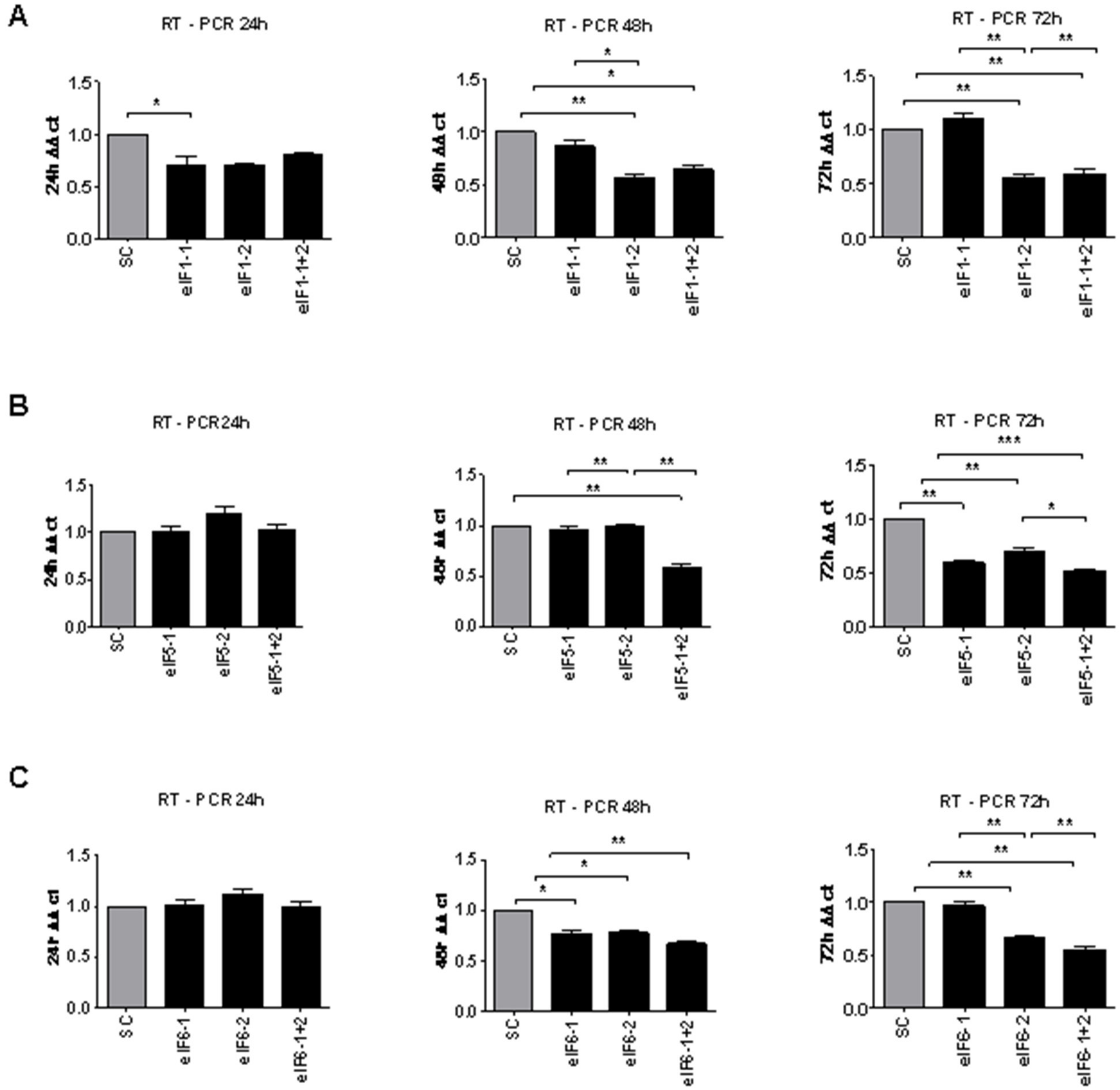

Supplementary Figure 20: *in vitro* characterization of the effect eIF1, eIF5 and eIF6 knockdown in HT29 cells on PARP rate. **(A)** mRNA expression of PARP in eIF1- silenced HT29 cells. **(B)** mRNA expression of PARP in eIF5- silenced HT29 cells. **(C)** mRNA expression of PARP in eIF6- silenced HT29 cells. Three independent experiments were carried out. Bars represent mean ± SEM. $^{*}p < 0.05$, $^{**}p < 0.01$, $^{***}p < 0.001$. Statistical analysis: 2-way ANOVA with Bonferroni posttest.

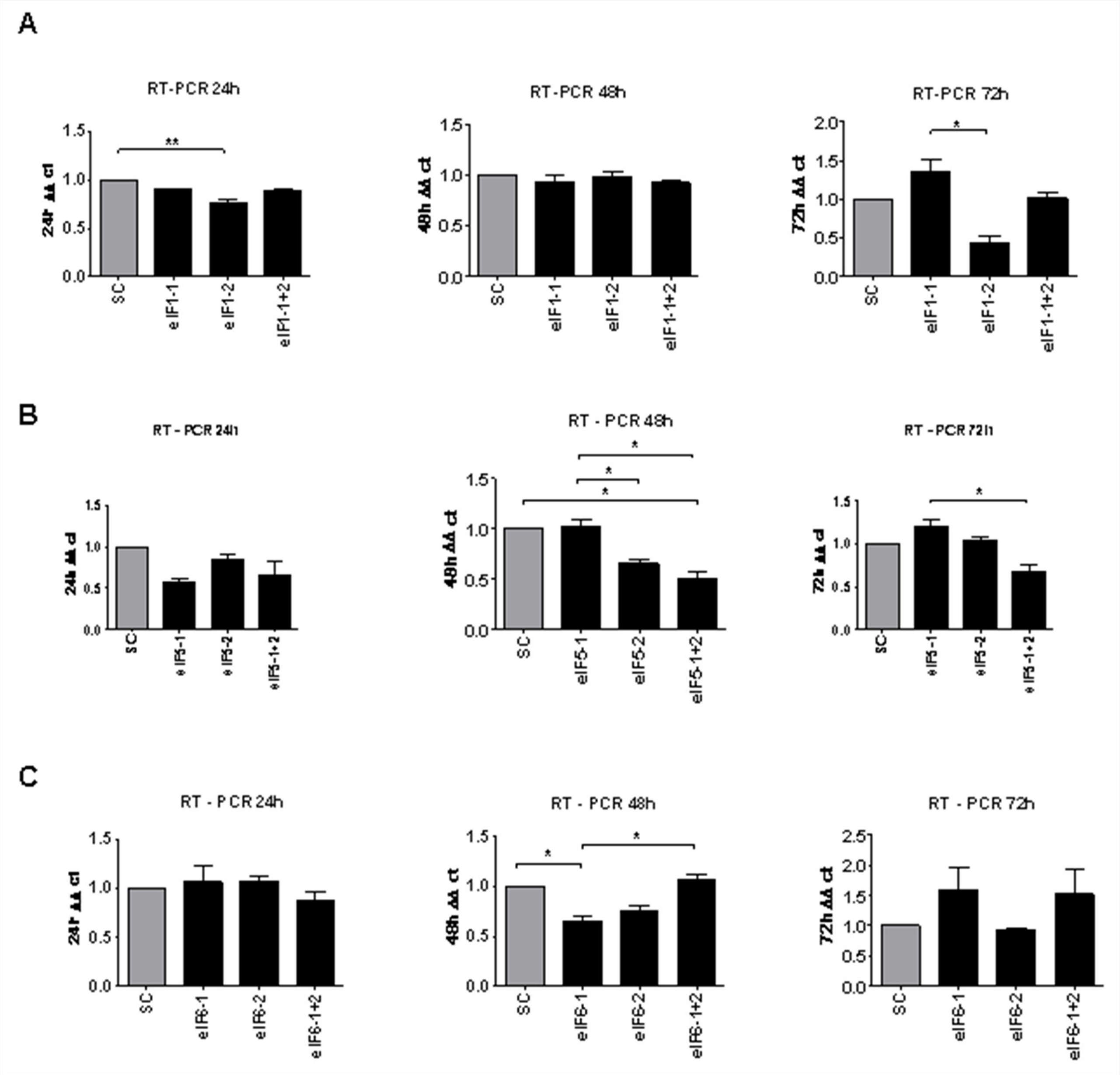

Supplementary Figure 21: ***in vitro*** **characterization of the effect eIF1, eIF5 and eIF6 knockdown in HCT116 cells on caspase 3.** **(A)** mRNA expression of caspase 3 in eIF1- silenced HCT116 cells. **(B)** mRNA expression of caspase 3 in eIF5- silenced d HCT116 cells. **(C)** mRNA expression of caspase 3 in eIF6- silenced HCT116 cells. Three independent experiments were carried out. Bars represent mean ± SEM. $^{*}p < 0.05$, $^{**}p < 0.01$, $^{***}p < 0.001$. Statistical analysis: 2-way ANOVA with Bonferroni posttest.

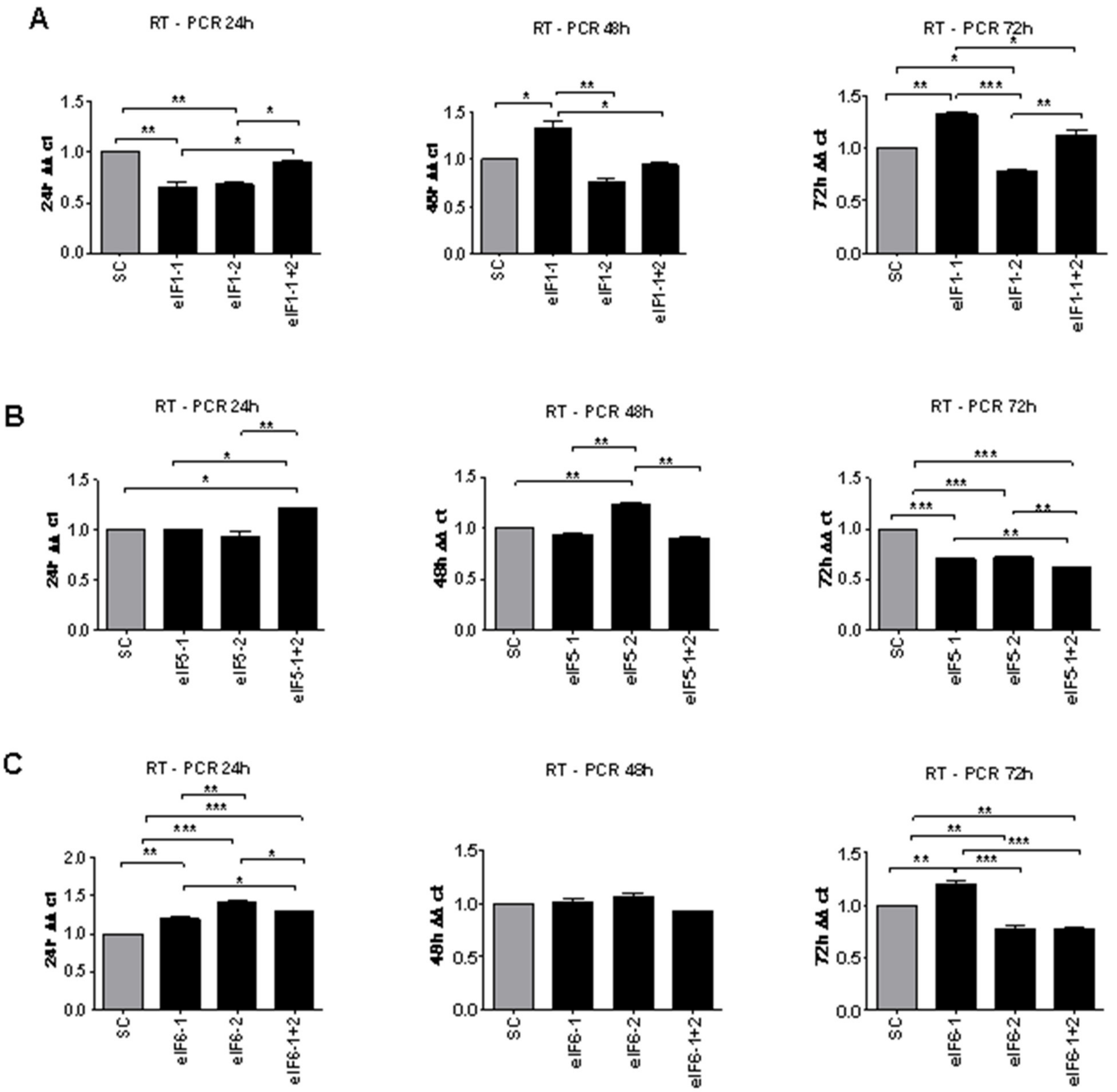

Supplementary Figure 22: *in vitro* characterization of the effect eIF1, eIF5 and eIF6 knockdown in HT29 cells on caspase 3 rate. (A) mRNA expression of caspase 3 in eIF1- silenced HT29 cells. **(B)** mRNA expression of caspase 3 in eIF5- silenced d HT29 cells. **(C)** mRNA expression of caspase 3 in eIF6-transfected HT29 cells. Three independent experiments were carried out. Results are presented as mean ± SEM. $^{*}p < 0.05$, $^{**}p < 0.01$, $^{***}p < 0.001$. Statistical analysis: 2-way ANOVA with Bonferroni posttest.

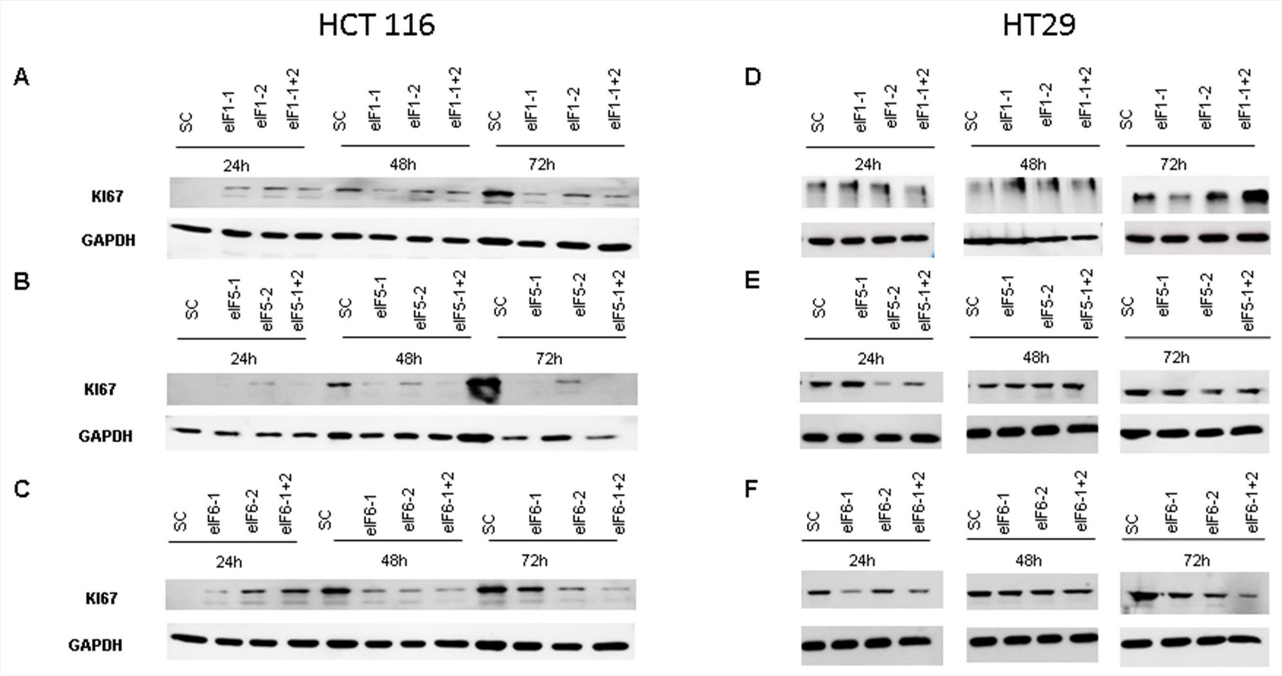

Supplementary Figure 23: *in vitro* characterization of the effect eIF1, eIF5 and eIF6 knockdown in HCT116 and HT29 cells on cell proliferation. **(A)** Protein expression of Ki67 in eIF1- silenced HCT116 cells. **(B)** Protein expression of Ki67 in eIF5-silenced HCT116 cells. **(C)** Protein expression of Ki67 in eIF6- silenced HCT116 cells. **(D)** Protein expression of Ki67 in eIF1-silenced HT29 cells. **(E)** Protein expression of Ki67 in eIF5- silenced HT29 cells. **(F)** Protein expression of Ki67 in eIF6- silenced HT29 cells Three independent experiments were carried out. Bars represent mean ± SEM. $^{*}p < 0.05$, $^{**}p < 0.01$, $^{***}p < 0.001$. Statistical analysis: 2-way ANOVA with Bonferroni posttest.

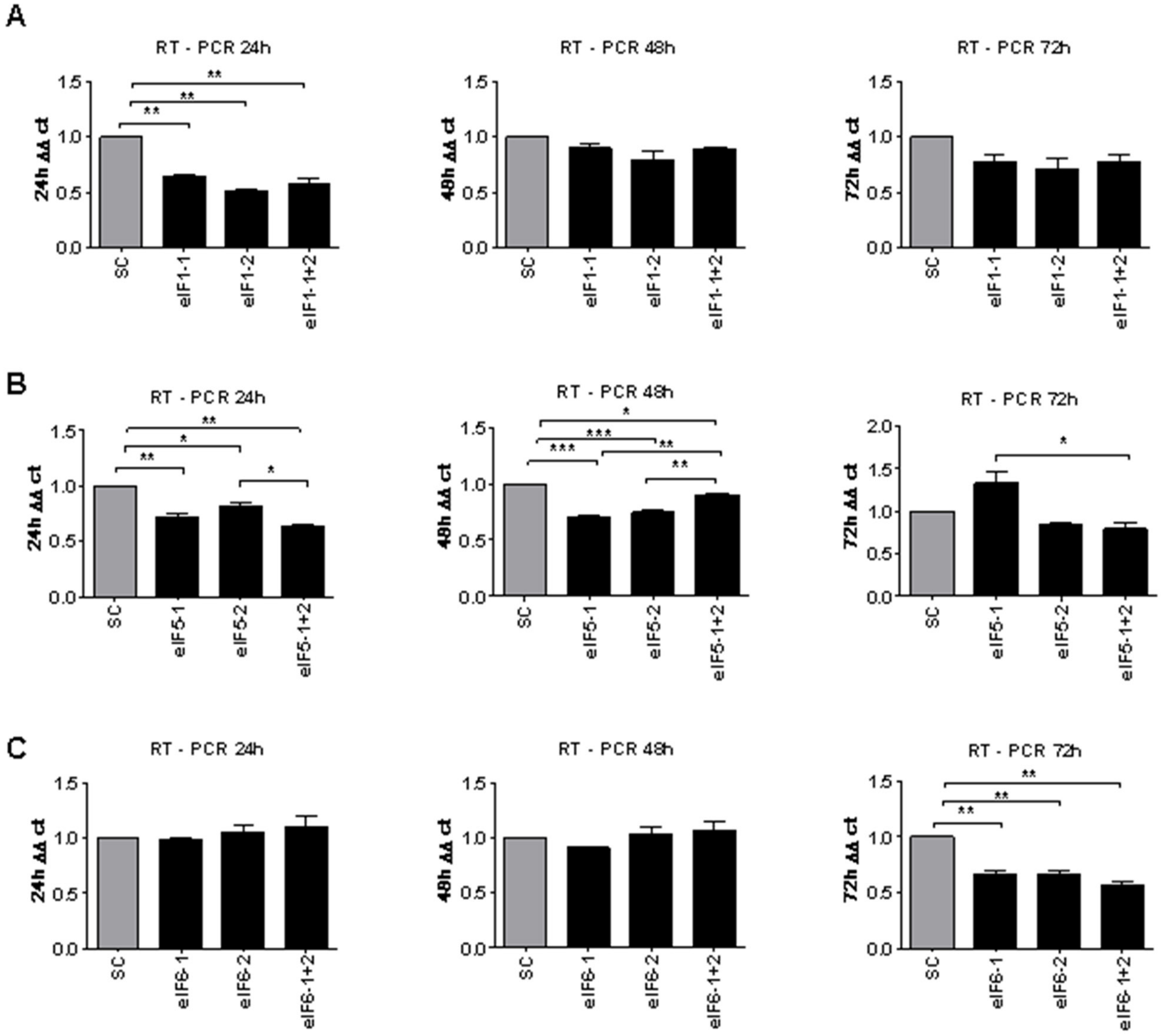

Supplementary Figure 24: *in vitro* characterization of the effect eIF1, eIF5 and eIF6 knockdown in HCT116 cells on Ki67. (A) mRNA expression of Ki67 in eIF1- silenced HCT116 cells. **(B)** mRNA expression of Ki67 in eIF5- silenced HCT116 cells **(C)** mRNA expression of Ki67 in eIF6- silenced HCT116 cells. Three independent experiments were carried out. Bars represent mean ± SEM. $^{*}p < 0.05$, $^{**}p < 0.01$, $^{***}p < 0.001$. Statistical analysis: 2-way ANOVA with Bonferroni posttest.

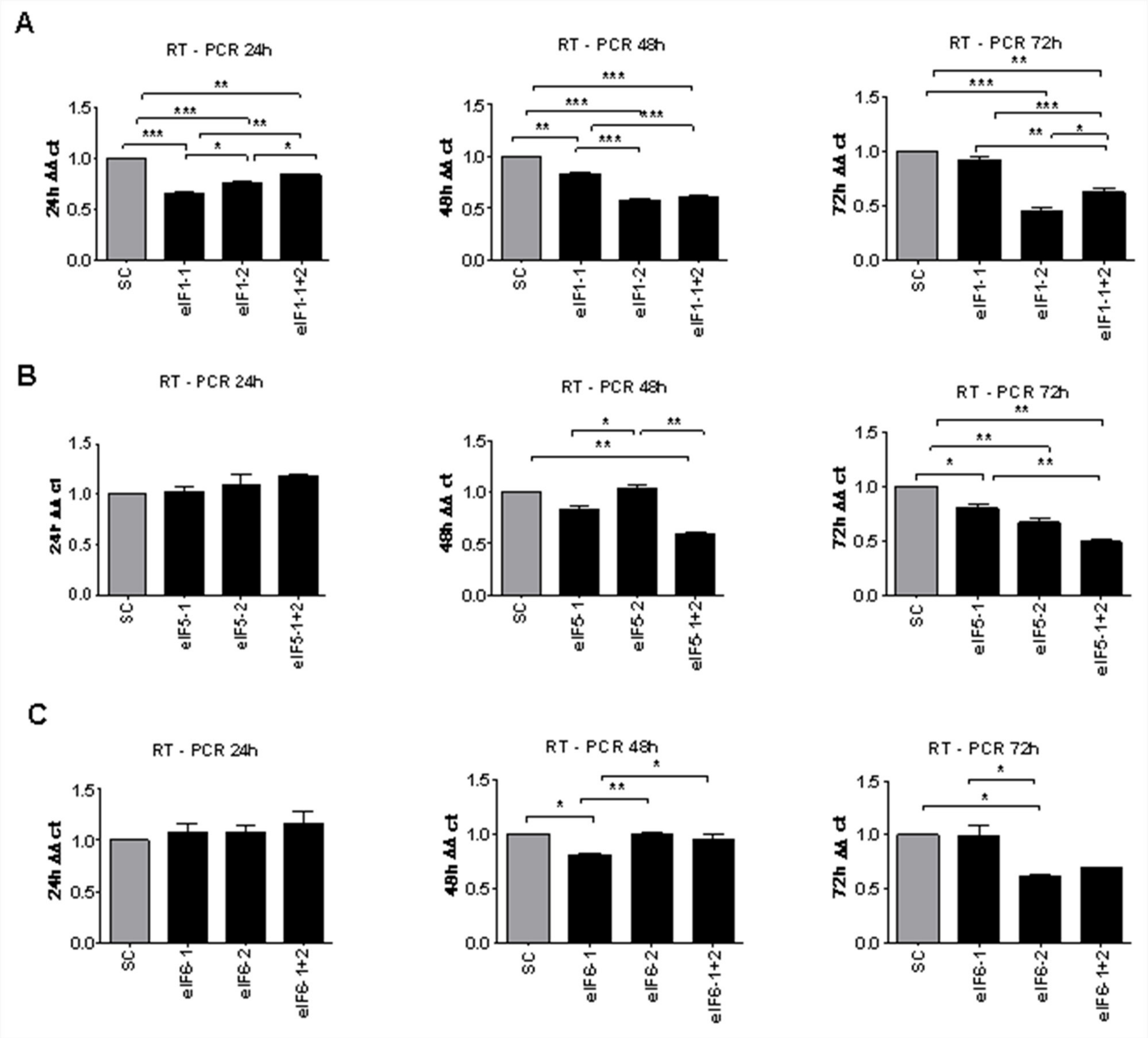

Supplementary Figure 25: *in vitro* characterization of the effect eIF1, eIF5 and eIF6 knockdown in HT29 cells on Ki67. **(A)** mRNA expression of Ki67 in eIF1 silenced HT29 cells. **(B)** mRNA expression of Ki67 in eIF5- silenced HT29 cells **(C)** mRNA expression of Ki67 in eIF6- silenced HT29 cells. Three independent experiments were carried out. Bars represent mean ± SEM. $^{*}p < 0.05$, $^{**}p < 0.01$, $^{***}p < 0.001$. Statistical analysis: 2-way ANOVA with Bonferroni posttest.

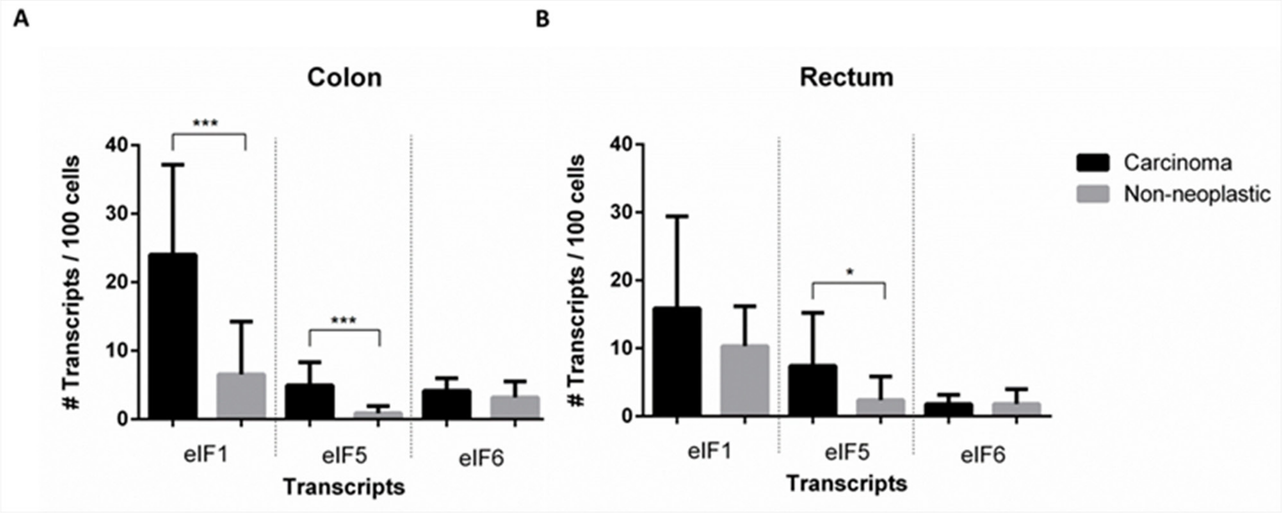

Supplementary Figure 26: Quantification of *in situ* detected signals of eIF1, eIF5 and eIF6 transcripts in colon and rectum carcinoma tissue vs. non-neoplastic tissue. **(A)** mRNA expression level of eIF1, eIF5 and eIF6 in colon carcinoma (CC) compared to non-neoplastic tissue (NNT). **(B)** mRNA expression level of eIF1, eIF5 and eIF6 in rectum carcinoma compared to non-neoplastic tissue. Bars represent mean ± SEM. $^{*}p < 0.05$, $^{**}p < 0.01$, $^{***}p < 0.001$.

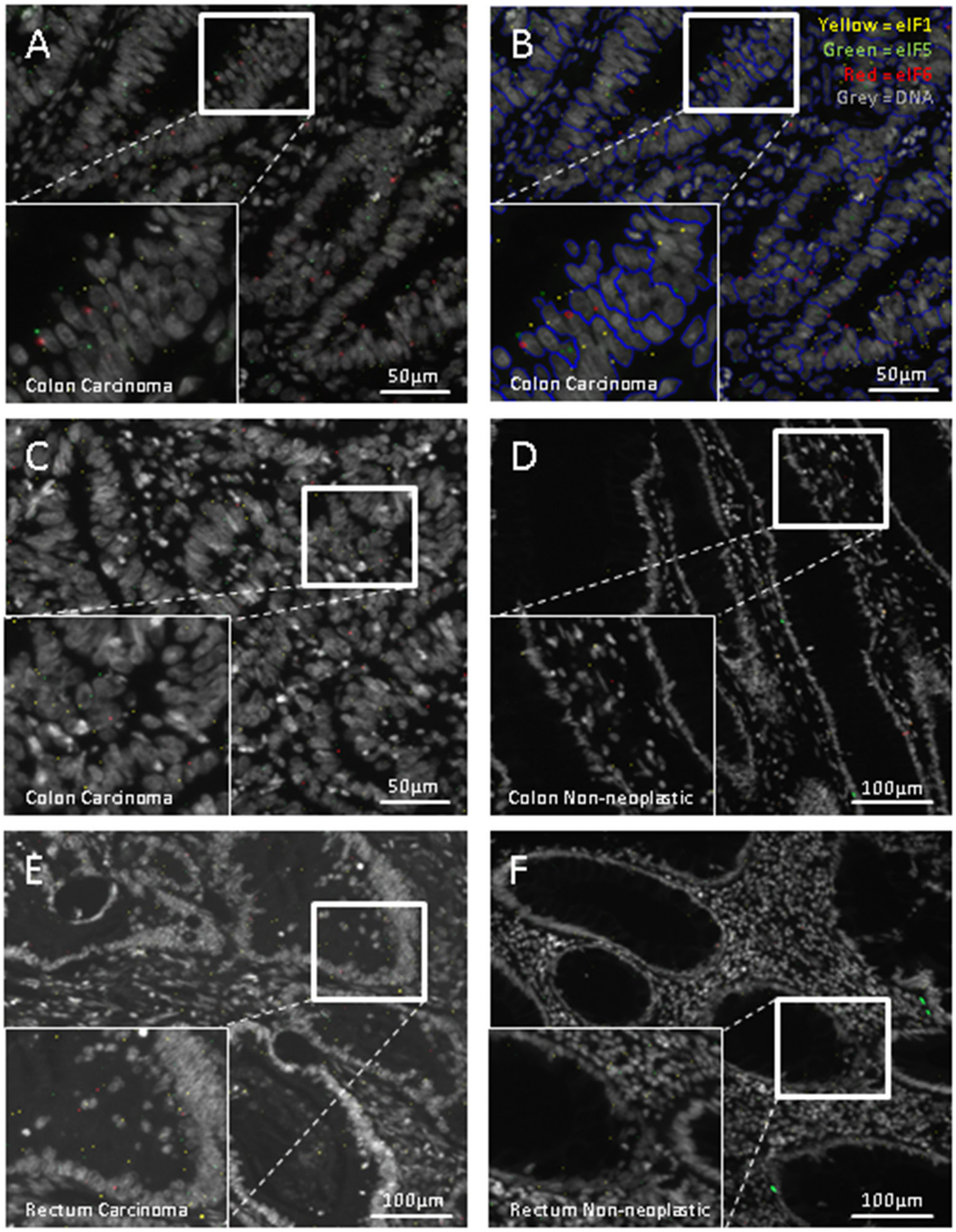

Supplementary Figure 27: *In situ* detection of eIF transcripts in colorectal carcinoma (CRC). mRNA transcripts are visualized by in situ padlock probe technology and can be quantified by counting the number of signals. Each single fluorescent signal corresponds to a certain mRNA transcript according to the color (eIF1 = yellow, eIF6 = green, eIF6 = red). **(A)** A representative image of a colon cancer (CC) sample. For better clarity, a region was magnified. **(B)** Output image of the quantification of each signal by the CellProfiler software. **(C)** The tissue displays small fluorescent signals in rectum carcinoma (RC) samples. **(D)** The tissue displays small fluorescent signals in non-neoplastic tissue (NNT). **(E)** The tissue displays small fluorescent signals in colon carcinoma (CC) samples. **(F)** The tissue displays small fluorescent signals in non-neoplastic tissue (NNT).

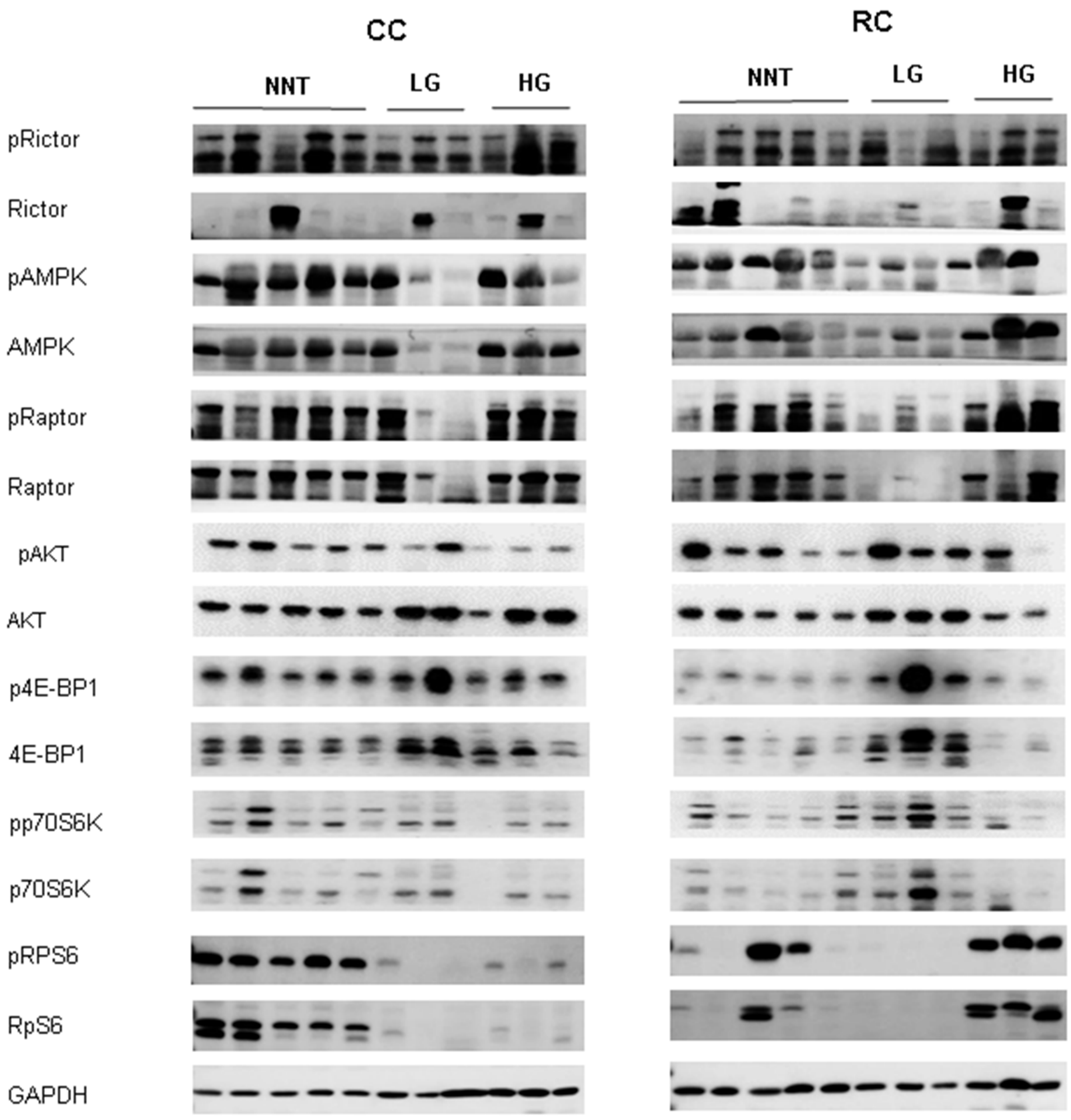

Supplementary Figure 28: PI3K/AKT/mTOR pathway expression in low and high grade colon and rectum carcinomas. Immunoblot analyses from low grade (LG) and high grade (HG) colon carcinomas (CC) and rectum carcinomas (RC) compared to non-neoplastic tissues (NNT). Equal amounts of protein were resolved on SDS PAGE and immunoblotted with pRictor, Rictor, pAMPK, AMPK, pRaptor, Raptor, p4E-BP1, pmTOR, mTOR, pAKT, AKT, pp70S6K, p70S6K, pRPS6, RPS6,p4E-BP1, 4E-BP1 and GAPDH (loading control) antibodies. Three independent experiments were carried out. Bars represent mean ± SEM. $^{*}p < 0.05$, $^{**}p < 0.01$, $^{***}p < 0.001$. Statistical analysis: 2-way ANOVA with Bonferroni posttest.

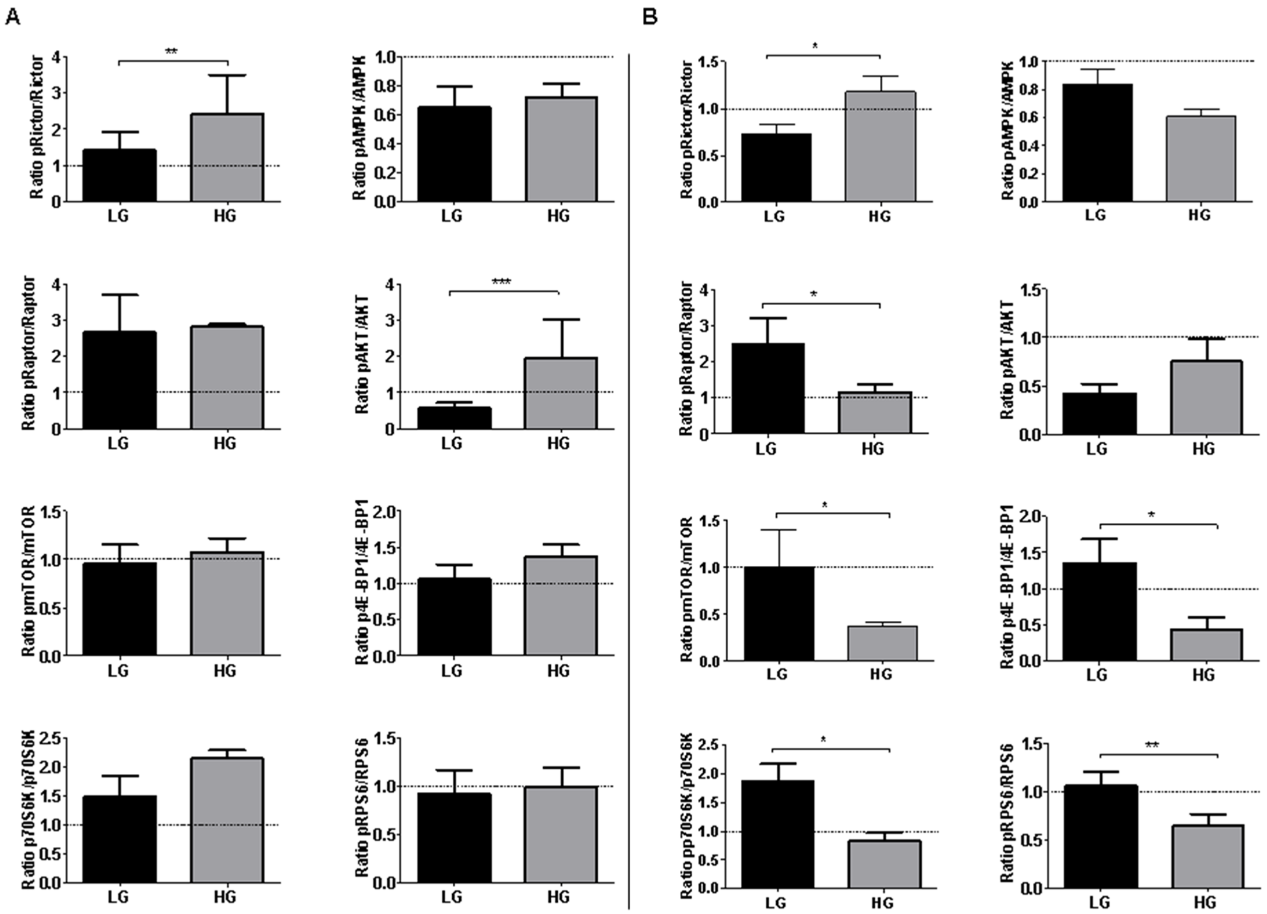

Supplementary Figure 29: mTOR expression levels in low and high grade colon and rectum carcinomas. **(A)** Graphs showing pRictor/ Rictor ratio, pAMPK/ AMPK ratio, pRaptor/ Raptor ratio, pmTOR/ mTOR ratio, pAKT/ AKT ratio, pp70S6K/ p70S6K ratio, pRPS6/ RPS6 ratio and p4E-BP1/4E-BP1 ratio in low grade (LG) and high grade (HG) colon carcinomas (CC), normalized to β-actin. **(B)** Graphs showing pRictor/ Rictor ratio, pAMPK/ AMPK ratio, pRaptor/ Raptor ratio, pmTOR/ mTOR ratio, pAKT/ AKT ratio, pp70S6K/ p70S6K ratio, pRPS6/ RPS6 ratio and p4E-BP1/4E-BP1 ratio in LG and HG RC, normalized to GAPDH and NNT (set as 1). Three independent experiments were carried out. Bars represent mean ± SEM. $^{*}p < 0.05$, $^{**}p < 0.01$, $^{***}p < 0.001$. Statistical analysis: 2-way ANOVA with Bonferroni posttest.

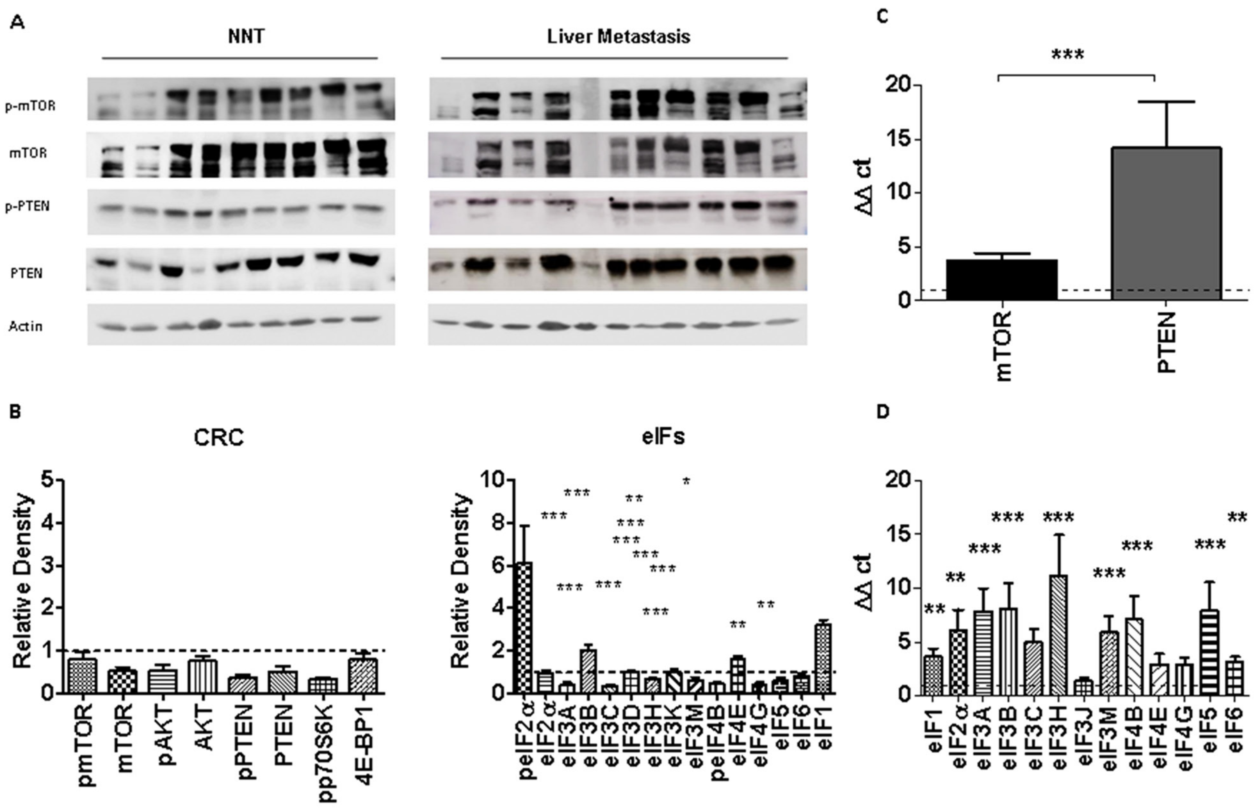

Supplementary Figure 30: PI3K/AKT/mTOR signaling pathway members and eIF subunits in liver metastases of CRC patients. **(A)** Protein expression of mTOR members in CRC Met tissues. **(B)** Densitometric analyses of mTOR members, eIF subunits and β-actin in liver metastases of CRC patient samples compared to non-neoplastic tissues (NNT). **(C)** mRNA expression of mTOR and PTEN in liver metastases of primary CRC patient samples. **(D)** mRNA expression of eIFs in liver metastases of primary CRC patient samples. Three independent experiments were carried out. Bars represent mean ± SEM. $^{*}p < 0.05$, $^{**}p < 0.01$, $^{***}p < 0.001$. Statistical analysis: 2-way ANOVA with Bonferroni posttest.

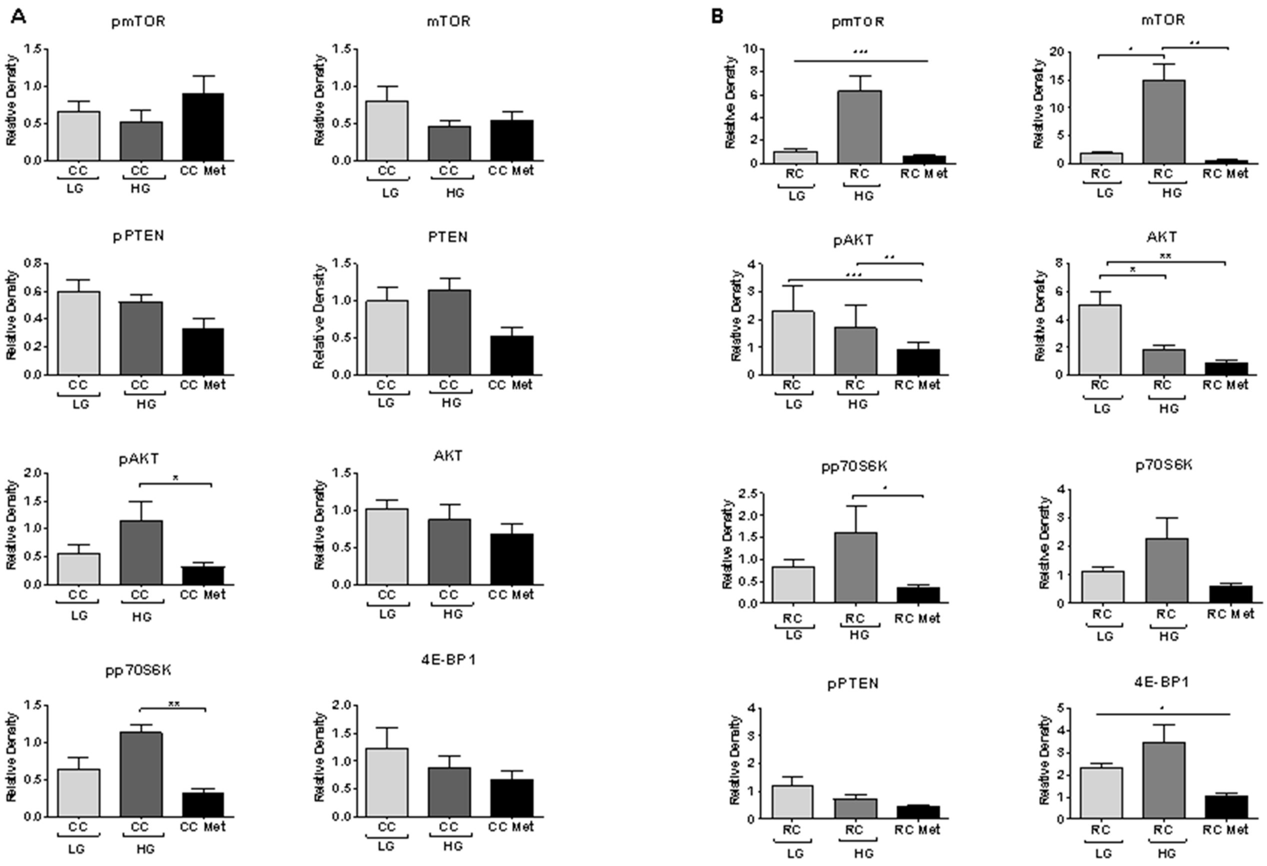

Supplementary Figure 31: PI3K/AKT/mTOR signaling pathway members in liver metastases of CRC patients. **(A)** Densitometric analyses of mTOR members and β-actin in liver metastases of primary CC patient samples compared to primary low (LG) and high grade (HG) colon carcinoma (CC) samples. **(B)** Densitometric analyses of mTOR members and β-actin in liver metastases of primary rectum carcinoma (RC) patient samples compared to primary low and high grade RC patient samples. Three independent experiments were carried out. Bars represent mean ± SEM. $^{*}p < 0.05$, $^{**}p < 0.01$, $^{***}p < 0.001$. Statistical analysis: 2-way ANOVA with Bonferroni posttest.

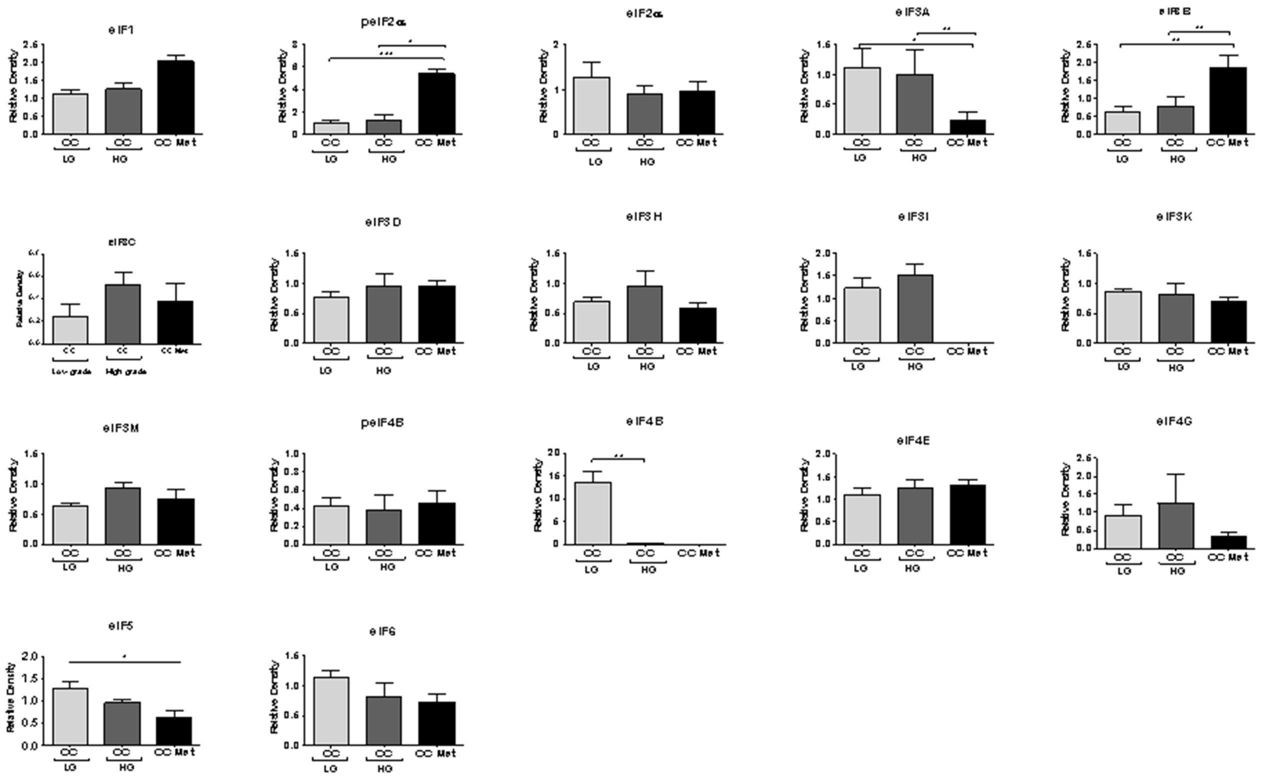

Supplementary Figure 32: eIF expression levels in liver metastases of CC patients. Densitometric analyses of mTOR members and β-actin in liver metastases of primary CC patient samples compared to primary low (LG) and high grade (HG) colon carcinoma (CC) patient samples. Three independent experiments were carried out. Bars represent mean ± SEM. $^{*}p < 0.05$, $^{**}p < 0.01$, $^{***}p < 0.001$. Statistical analysis: 2-way ANOVA with Bonferroni posttest.

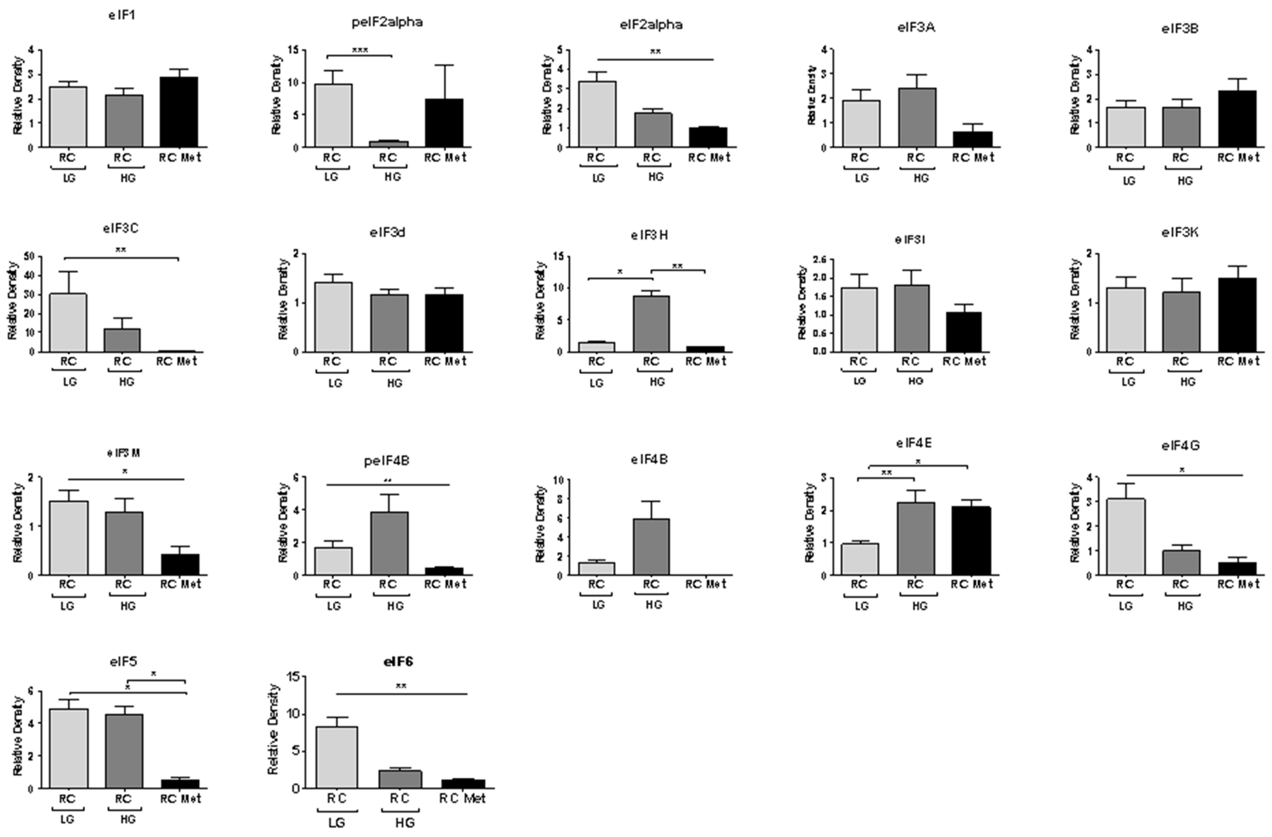

Supplementary Figure 33: eIF expression levels in liver metastases of RC patients. Densitometric analyses of mTOR members and β-actin in liver metastases of primary RC patient samples compared to primary low (LG) and high grade (HG) rectum carcinoma (RC) patient samples. Three independent experiments were carried out. Bars represent mean ± SEM. $^{*}p < 0.05$, $^{**}p < 0.01$, $^{***}p < 0.001$. Statistical analysis: 2-way ANOVA with Bonferroni posttest.

Supplementary Table 1: Protein expression levels of eIF1, eIF5 and eIF6 in CRC, CC, RC, low grade CC and RC and high grade CC and RC. Descriptive statistics and Mann-Whitney-U-Test are performed for CRC, low and high grade CC and RC in comparison to non-neoplastic tissue

Total		N	Average	*SD*	Median	p-value
**eIF1**	Colon	17	7	0.0007	0.0005	0.1879
	Rectum	26	0.0006	0.0008	0.0003	
**eIF5**	Colon	17	12	0.3904	11	<0.0001
	Rectum	26	38	19	40	
**eIF6**	Colon	17	11	0.4222	0.905	0.0171
	Rectum	26	49	49	30	
**Colon**						
**eIF1**	Low	12	0.0004	0.0004	0.0003	0.0114
	High	5	0.0014	0.0008	0.0018	
**eIF5**	Low	12	13	0.447	12	0.3990
	High	5	10	0.1162	11	
**eIF6**	Low	12	12	0.3797	11	0.0268
	High	5	0.7574	0.3991	0.5794	
**Rectum**						
**eIF1**	Low	13	0.0005	0.0003	0.0004	0.1175
	High	13	0.0007	0.0012	0.0001	
**eIF5**	Low	13	46	16	42	0.1062
	High	13	31	20	24	
**eIF6**	Low	13	82	51	80	0.0011
	High	13	16	13	15	

Supplementary Table 2: mRNA expression levels of eIF1, eIF5 and eIF6 in CRC, CC, RC, low grade CC and RC and high grade CC and RC. Descriptive statistics and Mann-Whitney-U-Test are performed for CRC, low and high grade CC and RC in comparison to non-neoplastic tissue

**Total**		**N**	**Average**	***SD***	**Median**	**p-value**
**eIF1**	Colon	13	174	101	174	0.0020
	Rectum	17	74	115	15	
**eIF5**	Colon	13	18	18	11	0.0002
	Rectum	17	75	71	49	
**eIF6**	Colon	13	43	46	36	0.3003
	Rectum	17	54	45	55	
**Colon**						
**eIF1**	Low	10	145	81	148	-
	High	3			236	
**eIF5**	Low	10	20	20	13	-
	High	3			0.545	
**eIF6**	Low	10	47	51	46	-
	High	3			29	
**Rectum**						
**eIF1**	Low	5	2,013	131	139	0.0003
	High	12	16	20	0.8045	
**eIF5**	Low	5	118	102	64	0.1037
	High	12	56	49	46	
**eIF6**	Low	5	32	24	23	0.4421
	High	12	63	49	60	

Supplementary Table 3: Protein and mRNA expression levels of mTOR members and eIF subunits in CRC. Descriptive statistics and Mann-Whitney-U-Test are performed for CRC in comparison to non-neoplastic tissue

		Total number	Average	*SD*	Median	p-value
Protein_pAKT	Colon	17	0.6946	00.623	0.5163	0.1119
	Rectum	26	14.064	14.950	11.296	
Protein_AKT	Colon	13	0.9796	0.4207	0.8389	0.0000
	Rectum	17	33.265	30.880	28.183	
Protein_pmTOR	Colon	17	0.6183	0.4807	0.5463	0.0014
	Rectum	26	31.296	33.950	14.109	
Protein_mTOR	Colon	17	0.7167	0.6463	0.6522	0.0001
	Rectum	26	72.539	83.166	25.192	
RNA_mTOR	Colon	13	20.350	23.903	11.897	0.0982
	Rectum	17	17.328	22.018	0.7100	
Protein_pPTEN	Colon	17	0.5796	0.2657	0.5849	0.0660
	Rectum	26	10.315	0.9812	0.8067	
Protein_PTEN	Colon	17	10.313	0.5764	0.9496	0.4713
	Rectum	26	0.9071	0.4900	0.9066	
RNA_PTEN	Colon	13	30.084	20.898	32.888	0.0161
	Rectum	17	13.337	17.429	0.8232	
Protein_ pp70S6K	Colon	17	0.7894	0.4094	0.6662	0.1037
	Rectum	26	12.269	0.9465	0.8975	
Protein_4E-BP	Colon	17	11.334	11.626	0.7832	0.0000
	Rectum	26	30.889	16.396	27.651	
Protein_peIF2α	Colon	17	10.491	0.9174	0.6412	0.0029
	Rectum	26	53.307	68.526	17.104	
Protein_eIF2α	Colon	17	11.837	10.339	0.8665	0.0002
	Rectum	26	24.463	16.013	20.959	
RNA_eIF2α	Colon	13	44.694	74.809	19.118	0.2674
	Rectum	17	126.346	118.437	114.545	
Protein_eIF3A	Colon	17	10.889	10.250	0.4921	0.0369
	Rectum	26	19.210	15.169	16.351	
RNA_eIF3A	Colon	13	50.126	29.166	44.464	0.9499
	Rectum	17	68.213	71.769	38.581	
Protein_eIF3B	Colon	17	0.6731	0.5245	0.5700	0.0003
	Rectum	26	17.269	11.375	16.128	
RNA_eIF3B	Colon	13	38.328	25.048	32.304	0.0054
	Rectum	17	224.336	208.793	155.204	

(Continued)

		Total number	Average	*SD*	Median	p-value
Protein_eIF3C	Colon	17	0.3141	0.2962	0.2253	0.0000
	Rectum	13	31.740	18.197	30.546	
RNA_eIF3C	Colon	13	194.982	83.986	196.061	0.0226
	Rectum	17	115.276	136.372	60.490	
Protein_eIF3D	Colon	17	0.8200	0.2993	0.7408	0.0007
	Rectum	26	12.805	0.4456	11.745	
Protein_eIF3H	Colon	17	0.7571	0.2787	0.6529	0.0000
	Rectum	26	38.020	34.318	20.753	
RNA_eIF3H	Colon	13	82.621	54.638	59.427	0.0079
	Rectum	17	38.531	39.754	24.317	
Protein_eIF3I	Colon	17	12.947	0.7444	11.025	0.6019
	Rectum	26	16.210	11.814	13.977	
RNA_eIF3I	Colon	13	44.180	71.673	21.774	0.0069
	Rectum	17	218.604	201.068	172.608	
Protein_eIF3K	Colon	17	0.9415	0.2733	0.8740	0.2142
	Rectum	26	12.309	0.7982	13.424	
Protein_eIF3M	Colon	17	0.7000	0.2437	0.6989	0.0010
	Rectum	26	13.673	0.7076	11.531	
RNA_eIF3M	Colon	13	186.658	84.427	200.925	0.0003
	Rectum	17	64.375	56.804	42.973	
Protein_peIF4B	Colon	17	0.4104	0.3452	0.2605	0.0000
	Rectum	26	19.451	19.047	11.889	
Protein_eIF4B	Colon	17	73.464	59.715	85.315	0.0590
	Rectum	26	31.267	36.893	15.518	
RNA_eIF4B	Colon	13	120.954	133.193	47.015	0.6603
	Rectum	17	118.500	115.764	91.398	
Protein_eIF4E	Colon	17	11.977	0.3708	12.283	0.5024
	Rectum	26	14.536	0.8670	11.956	
RNA_eIF4E	Colon	13	0.4702	0.5207	0.2300	0.1373
	Rectum	17	11.533	15.770	0.3894	
Protein_eIF4G	Colon	17	0.9922	11.496	0.4095	0.0238
	Rectum	26	20.349	20.159	14.656	
RNA_eIF4G	Colon	13	27.179	15.887	29.235	0.2018
	Rectum	17	85.873	82.389	85.065	

Supplementary Table 4: Protein and mRNA expression levels of mTOR members and eIF subunits in low and high grade CC. Descriptive statistics and Mann-Whitney-U-Test are performed for low and high grade CC in comparison to non-neoplastic tissue

		Total number	Average	*SD*	Median	p-value
Protein_pAKT	Colon	17	0.6946	00.623	0.5163	0.1119
	Rectum	26	14.064	14.950	11.296	
Protein_AKT	Colon	13	0.9796	0.4207	0.8389	0.0000
	Rectum	17	33.265	30.880	28.183	
Protein_pmTOR	Colon	17	0.6183	0.4807	0.5463	0.0014
	Rectum	26	31.296	33.950	14.109	
Protein_mTOR	Colon	17	0.7167	0.6463	0.6522	0.0001
	Rectum	26	72.539	83.166	25.192	
RNA_mTOR	Colon	13	20.350	23.903	11.897	0.0982
	Rectum	17	17.328	22.018	0.7100	
Protein_pPTEN	Colon	17	0.5796	0.2657	0.5849	0.0660
	Rectum	26	10.315	0.9812	0.8067	
Protein_PTEN	Colon	17	10.313	0.5764	0.9496	0.4713
	Rectum	26	0.9071	0.4900	0.9066	
RNA_PTEN	Colon	13	30.084	20.898	32.888	0.0161
	Rectum	17	13.337	17.429	0.8232	
Protein_ pp70S6K	Colon	17	0.7894	0.4094	0.6662	0.1037
	Rectum	26	12.269	0.9465	0.8975	
Protein_4E-BP	Colon	17	11.334	11.626	0.7832	0.0000
	Rectum	26	30.889	16.396	27.651	
Protein_peIF2α	Colon	17	10.491	0.9174	0.6412	0.0029
	Rectum	26	53.307	68.526	17.104	
Protein_eIF2α	Colon	17	11.837	10.339	0.8665	0.0002
	Rectum	26	24.463	16.013	20.959	
RNA_eIF2α	Colon	13	44.694	74.809	19.118	0.2674
	Rectum	17	126.346	118.437	114.545	
Protein_eIF3A	Colon	17	10.889	10.250	0.4921	0.0369
	Rectum	26	19.210	15.169	16.351	
RNA_eIF3A	Colon	13	50.126	29.166	44.464	0.9499
	Rectum	17	68.213	71.769	38.581	
Protein_eIF3B	Colon	17	0.6731	0.5245	0.5700	0.0003
	Rectum	26	17.269	11.375	16.128	
RNA_eIF3B	Colon	13	38.328	25.048	32.304	0.0054
	Rectum	17	224.336	208.793	155.204	

(Continued)

		Total number	Average	*SD*	Median	p-value
Protein_eIF3C	Colon	17	0.3141	0.2962	0.2253	0.0000
	Rectum	13	31.740	18.197	30.546	
RNA_eIF3C	Colon	13	194.982	83.986	196.061	0.0226
	Rectum	17	115.276	136.372	60.490	
Protein_eIF3D	Colon	17	0.8200	0.2993	0.7408	0.0007
	Rectum	26	12.805	0.4456	11.745	
Protein_eIF3H	Colon	17	0.7571	0.2787	0.6529	0.0000
	Rectum	26	38.020	34.318	20.753	
RNA_eIF3H	Colon	13	82.621	54.638	59.427	0.0079
	Rectum	17	38.531	39.754	24.317	
Protein_eIF3I	Colon	17	12.947	0.7444	11.025	0.6019
	Rectum	26	16.210	11.814	13.977	
RNA_eIF3I	Colon	13	44.180	71.673	21.774	0.0069
	Rectum	17	218.604	201.068	172.608	
Protein_eIF3K	Colon	17	0.9415	0.2733	0.8740	0.2142
	Rectum	26	12.309	0.7982	13.424	
Protein_eIF3M	Colon	17	0.7000	0.2437	0.6989	0.0010
	Rectum	26	13.673	0.7076	11.531	
RNA_eIF3M	Colon	13	186.658	84.427	200.925	0.0003
	Rectum	17	64.375	56.804	42.973	
Protein_peIF4B	Colon	17	0.4104	0.3452	0.2605	0.0000
	Rectum	26	19.451	19.047	11.889	
Protein_eIF4B	Colon	17	73.464	59.715	85.315	0.0590
	Rectum	26	31.267	36.893	15.518	
RNA_eIF4B	Colon	13	120.954	133.193	47.015	0.6603
	Rectum	17	118.500	115.764	91.398	
Protein_eIF4E	Colon	17	11.977	0.3708	12.283	0.5024
	Rectum	26	14.536	0.8670	11.956	
RNA_eIF4E	Colon	13	0.4702	0.5207	0.2300	0.1373
	Rectum	17	11.533	15.770	0.3894	
Protein_eIF4G	Colon	17	0.9922	11.496	0.4095	0.0238
	Rectum	26	20.349	20.159	14.656	
RNA_eIF4G	Colon	13	27.179	15.887	29.235	0.2018
	Rectum	17	85.873	82.389	85.065	

Supplementary Table 5: Protein and mRNA expression levels of mTOR members and eIF subunits in low and high grade RC. Descriptive statistics and Mann-Whitney-U-Test are performed for low and high grade RC in comparison to non-neoplastic tissue

		Total number	Average	*SD*	Median	p-value
Protein_pAKT	Low Grade	13	14.130	10.676	12.955	0.5215
	High Grade	13	13.997	18.753	0.6909	
Protein_AKT	Low Grade	13	50.361	35.770	35.265	0.0001
	High Grade	13	16.169	0.8598	14.037	
Protein_pmTOR	Low Grade	13	10.635	0.7152	12.653	0.0061
	High Grade	13	51.956	37.753	61.388	
Protein_mTOR	Low Grade	13	17.935	12.737	15.284	0.0032
	High Grade	13	127.143	88.245	174.692	
RNA_mTOR	Low Grade	5	31.650	31.248	12.916	0.0911
	High Grade	12	11.360	14.765	0.6973	
Protein_pPTEN	Low Grade	13	11.941	12.877	0.9427	0.7389
	High Grade	13	0.8689	0.5389	0.7480	
Protein_PTEN	Low Grade	13	0.9373	0.5414	0.9100	0.8175
	High Grade	13	0.8769	0.4528	0.8831	
RNA_PTEN	Low Grade	5	16.642	0.5496	15.303	0.0084
	High Grade	12	11.960	20.588	0.7221	
Protein_ pp70S6K	Low Grade	13	0.8526	0.4486	0.8491	0.1303
	High Grade	13	16.013	11.669	17.461	
Protein_4E-BP	Low Grade	13	22.866	0.8852	23.072	0.0111
	High Grade	13	38.912	18.500	37.988	
Protein_peIF2α	Low Grade	13	97.108	74.879	110.145	0.0000
	High Grade	13	0.9506	0.4405	0.8491	
Protein_eIF2α	Low Grade	13	34.079	17.211	32.566	0.0005
	High Grade	13	14.847	0.6138	12.098	
RNA_eIF2α	Low Grade	5	260.098	68.160	240.210	0.0061
	High Grade	12	70.616	84.818	16.283	
Protein_eIF3A	Low Grade	13	19.271	16.060	14.323	0.9795
	High Grade	13	19.149	14.881	20.048	
RNA_eIF3A	Low Grade	5	31.439	17.233	37.452	0.2918
	High Grade	12	83.536	80.704	56.669	
Protein_eIF3B	Low Grade	13	16.478	0.9224	16.048	0.8576
	High Grade	13	18.060	13.533	16.208	
RNA_eIF3B	Low Grade	5	394.974	244.632	507.534	0.0578
	High Grade	12	153.237	151.323	112.718	

(Continued)

		Total number	Average	*SD*	Median	p-value
Protein_eIF3C	Low Grade	13	31.740	18.197	30.546	
	High Grade	0				
RNA_eIF3C	Low Grade	5	246.599	153.994	222.104	0.0153
	High Grade	12	60.558	85.568	27.645	
Protein_eIF3D	Low Grade	13	14.219	0.5211	12.649	0.1439
	High Grade	13	11.392	0.3143	11.213	
Protein_eIF3H	Low Grade	13	14.972	0.6451	13.474	0.0044
	High Grade	13	61.068	35.511	61.523	
RNA_eIF3H	Low Grade	5	35.263	30.538	22.065	0.9161
	High Grade	12	39.892	44.190	26.371	
Protein_eIF3I	Low Grade	13	17.372	13.224	12.122	0.7389
	High Grade	13	15.048	10.629	15.833	
RNA_eIF3I	Low Grade	5	253.201	137.624	255.195	0.3428
	High Grade	12	204.188	226.156	133.988	
Protein_eIF3K	Low Grade	13	13.002	0.8101	14.983	0.6261
	High Grade	13	11.617	0.8128	13.338	
Protein_eIF3M	Low Grade	13	15.224	0.7545	15.437	0.3173
	High Grade	13	12.122	0.6495	11.315	
RNA_eIF3M	Low Grade	5	26.291	11.359	22.553	0.0578
	High Grade	12	80.243	60.930	65.693	
Protein_peIF4B	Low Grade	13	15.172	13.146	10.710	0.2090
	High Grade	13	23.729	23.309	16.186	
Protein_eIF4B	Low Grade	13	13.746	10.301	10.727	0.0171
	High Grade	13	48.789	45.435	25.021	
RNA_eIF4B	Low Grade	5	217.519	115.420	259.078	0.0269
	High Grade	12	77.242	91.295	27.342	
Protein_eIF4E	Low Grade	13	0.9566	0.4172	0.9880	0.0005
	High Grade	13	19.507	0.9256	16.908	
RNA_eIF4E	Low Grade	5	11.134	14.144	0.2553	1,0000
	High Grade	12	11.699	16.996	0.4228	
Protein_eIF4G	Low Grade	13	31.229	23.365	30.263	0.0111
	High Grade	13	0.9470	0.6652	0.7153	
RNA_eIF4G	Low Grade	5	148.122	49.387	132.146	0.0269
	High Grade	12	59.936	80.568	17.762	

Supplementary Table 6: Antibodies for immunohistochemistry

Primary Antibody	Company	Dilution	Second Antibody
eIF4G	Cell Signaling (#2498)	1:25	Rabbit
eIF4E	Cell Signaling (#9742)	1:100	Rabbit
eIF2α (D7D3) XP	Cell Signaling (#5324)	1:2000	Rabbit
eIF3P110 (B-6)	Santa Cruz (sc-74507)	1:250	Mouse
eIF6	Gene Tex (GTX63642)	1:75	Rabbit
eIF3M (V-21)	Santa Cruz (sc-133541)	1:30	Rabbit
eIF1	Sigma Aldrich (HPA043003)	1:50	Rabbit
eIF3A	Cell Signaling (#2538)	1:50	Rabbit
eIF3B (eIF3η D-9)	Santa Cruz (sc-137215)	1:100	Mouse
eIF3H (D9C1) XP	Cell Signaling (#3413)	1:750	Rabbit

Supplementary Table 7: Primary Antibodies for Western blot

Primary Antibody	Company	Dilution	Second Antibody	Phosphorylation site
Phospho-mTOR	Cell Signaling (#5536)	1:1000	Rabbit	Ser2448
mTOR	Cell Signaling (#2983)	1:1000	Rabbit	
Phospho-PTEN	Cell Signaling (#9551)	1:1000	Rabbit	Ser380
PTEN	Cell Signaling (#9559)	1:1000	Rabbit	
Phospho-P70S6K	Cell Signaling (#9204)	1:1000	Rabbit	Thr421/Ser424
P70S6K	Cell Signaling (#9202)	1:1000	Rabbit	
Phospho Akt	Cell Signaling (#4058)	1:1000	Rabbit	Ser473
Akt	Cell Signaling (#9272)	1:1000	Rabbit	
GAPDH	Cell Signaling (#2118)	1:3000	Rabbit	
Phospho 4E-BP1	Cell Signaling (#9456)	1:1000	Rabbit	Ser65
4E-BP1	Cell Signaling (#9452)	1:1000	Rabbit	
Anti-Actin	Sigma (A2103)	1:1000	Rabbit	
Phospho-AMPK	Cell Signaling (#2535)	1:1000	Rabbit	Thr172
AMPK	Cell Signaling (#5832)	1:1000	Rabbit	
Phospho-Rictor	Cell Signaling (#3806)	1:1000	Rabbit	Thr1135
Rictor	Cell Signaling (#2114)	1:1000	Rabbit	
Phospho-Raptor	Cell Signaling (#2083)	1:1000	Rabbit	Ser792
Raptor	Cell Signaling (#2280)	1:1000	Rabbit	Ser722/Ser792
eIF1	Sigma (HPA043003)	1:500	Rabbit	
Phospho-eIF2α (Ser51)(D9G8)	Cell Signaling (#3398)	1:1000	Rabid	Ser51
eIF2α (D7D3) XP	Cell Signaling (#5324)	1:1000	Rabbit	
eIF3A	Cell Signaling (#2538)	1:1000	Rabbit	
eIF3β (A-8) = eIF3I	Santa Cruz (sc-374155)	1:1000	Mouse	
eIF3C	Cell Signaling (#2068)	1:1000	Rabbit	
eIF3H (D9C1) XP	Cell Signaling (#3413)	1:1000	Rabbit	
eIF3J	Cell Signaling (#3261)	1:1000	Rabbit	
eIF3K (2313C2a)	Santa Cruz (sc-81262)	1:1000	Mouse	
eIF3M (V-21)	Santa Cruz (sc-133541)	1:500	Rabbit	
eIF3B = eIF3η D-9	Santa Cruz (sc-137215)	1:1000	Mouse	
eIF3P110 (B-6)	Santa Cruz (sc-74507)	1:500	Mouse	
eIF3θ (H-300)	Santa Cruz (sc-30149)	1:1000	Rabbit	
eIF3ζ (H-300) = eIF3D	Santa Cruz (sc-28856)	1:1000	Rabbit	
PhosphoeIF4B (Ser406)	Cell Signaling (#5399)	1.1000	Rabbit	Ser406

(Continued)

Primary Antibody	Company	Dilution	Second Antibody	Phosphorylation site
eIF4B	Cell Signaling (#3592)	1.1000	Rabbit	
eIF4E	Cell Signaling (#9742)	1:1000	Rabbit	
eIF4G	Cell Signaling (#2498)	1:1000	Rabbit	
eIF5	GeneTex (GTX114923)	1:500	Rabbit	
eIF6	Gene Tex (GTX63642)	1:1000	Rabbit	
PARP	Cell Signaling (#9542)	1:1000	Rabbit	
Cleaved Caspase-3	Cell Signaling (#9664)	1:1000	Rabbit	
Ki67	DAKO (#7240)	1:1000	Mouse	

Supplementary Table 8: Primer sequences for qRT-PCR

Gene	Primer Pair	Sequence (5'-3')	Length	Tm [°C]
**mTOR**	Fwd	ATGCTTGGAACCGGACCTG	19	60
	Rev	TCTTGACTCATCTCTCGGAGTT	22	58
**PTEN**	Fwd	TGGATTCGACTTAGACTTGACCT	23	58
	Rev	GGTGGGTTATGGTCTTCAAAAGG	23	59
**eIF2α**	Fwd	TGGTGAATGTCAGATCCATTGC	22	60
	Rev	TAGAACGGATACGCCTTCTGG	21	61
**eIF3A**	Fwd	GCCGGAAAATGCCCTCAAAC	20	62
	Rev	TGGTTCGTGTATCTTTTGCCAT	22	60
**eIF3B**	Fwd	GGACCCGACCGACTTGAGA	19	63
	Rev	TTGACCCGGAATGTGTGCTG	20	63
**eIF3J**	Fwd	GTCAAGGATAACTGGGATGACG	22	60
	Rev	CGAGGTCTGACTCTTCCTGTAA	22	61
**eIF4B**	Fwd	CCTCCCAGTCCACTCGAGCTG	21	65
	Rev	GCTTGGGTGTCTCTCCCGAGG	21	65
**eIF4G1**	Fwd	CCCGAAAAGAACCACGCAAG	20	62
	Rev	TTCCCCTCGATCCTTATCAGC	21	61
**eIF5**	Fwd	AGCGTGTCAGACCAGTTCTAT	21	61
	Rev	CTGTCTTGATTCCATTGCCTTTG	23	60
**eIF6**	Fwd	CCGCGTGCGGAGCTTGTAT	19	61
	Rev	CGCCCTCGAACACACTGTAGAAGT	24	64
**eIF4E**	Fwd	GACCTGACCTCCCGCGGACAA	21	65
	Rev	TGCCCATCTGTTCTGTAGGGGATG	24	64
**eIF3M**	Fwd	TCAGAAGAGAACTCGGAAGGTG	22	60
	Rev	ACCACACTGTTCATCACACTTT	22	57
**eIF3H**	Fwd	TCGGGAGATTCAGCCGTGA	19	59
	Rev	CTCCGCATCATTTCCATCTGATA	23	59
**eIF3C**	Fwd	TTTGGGCCTCGTGCTTCGTGG	21	64
	Rev	TCGCTCAGCAAACAATGGCTGTTTG	24	63
**eIF1**	Fwd	GAAACGGCAGGAAGACCCTTA	21	60
	Rev	CGGATGCTCAATTACAGTACCAT	23	59
**Ki67**	Fwd	AGACGCCTGGTTACTATCAAAG	19	60
	Rev	GGAAGCTGGATACGGATGTCA	22	58
**PARP**	Fwd	CGGTGACTTATCCTGTGGTCC	23	58
	Rev	ACATCCCGACAGAAAGGCAC	23	59
**Caspase 3**	Fwd	ATGGAAGCGAATCAATGGACTC	22	60
	Rev	CTGTACCAGACCCGAGATGTCA	21	61
